# A novel super-enhancer-driven lncRNA LINC00973 governs head and neck squamous cell carcinoma progression through EN2

**DOI:** 10.1038/s41419-025-08380-8

**Published:** 2025-12-19

**Authors:** An Wang, Pengfei Diao, Na Xiao, Yuxiang Wei, Yaping Wu, Yanling Wang, Xuejing Wang, Enshi Yan, Hongbing Jiang, Jin Li, Jie Cheng

**Affiliations:** 1 Department of Oral and Maxillofacial Surgery, The Affiliated Stomatological Hospital, Nanjing Medical University, Nanjing, Jiangsu China; 2 State Key Laboratory Cultivation Base of Research, Prevention and Treatment for Oral Diseases, Nanjing Medical University, Nanjing, Jiangsu China; 3 Jiangsu Province Engineering Research Center of Stomatological Translational Medicine, Nanjing Medical University, Nanjing, Jiangsu China

**Keywords:** Head and neck cancer, Oncogenes, Prognostic markers, Epigenetics, Long non-coding RNAs

## Abstract

Long noncoding RNAs (lncRNAs) have emerged as pivotal regulators driving cancer development and potential therapeutic targets across multiple human malignancies. However, their mechanistic roles during head and neck squamous cell carcinoma (HNSCC) initiation and progression remain incompletely elucidated. Here, we identified a novel oncogenic lncRNA, LINC00973, which was aberrantly upregulated in clinical samples and associated with aggressive clinicopathological features and adverse clinical outcomes. LINC00973 promoted cell proliferation, migration, and invasion and inhibited cell apoptosis and senescence in vitro, and induced tumor growth and lymph node metastasis in vivo. Mechanistically, LINC00973 functioned as a molecular sponge for tumor-suppressive miR-6756-3p, consequently stabilizing transcription factor Engrailed-2 (EN2) mRNA and activating NOTCH pathway to promote HNSCC progression. Integrative epigenomics/transcriptomics analyses coupled with molecular assays revealed a proximal super-enhancer (SE) within the LINC00973 locus, which recruited Activator Protein 1 (AP-1)/FOS Like 1 (FOSL1), BRD4, and EP300, thereby collectively activating its transcription. CRISPR interference assay identified four functional enhancer elements of LINC00973-SE and H3K27ac HiChIP data analysis suggested enhancer-promoter contacts, collectively contributing to LINC00973 transcription. Clinically, the abundance of LINC00973, miR-6756-3p, EN2, and NOTCH1 in HNSCC samples was correlated and significantly associated with patients’ survival. Collectively, our findings revealed a hitherto uncharacterized SE-driven LINC00973-miR-6756-3p-EN2 regulatory axis to facilitate HNSCC progression and highlighted LINC00973 as a promising prognostic biomarker and therapeutic target with considerable translational potential.

## Introduction

Head and neck squamous cell carcinoma (HNSCC), the sixth most prevalent malignancy worldwide, imposes a substantial socioeconomic burden and has profound impacts on patients’ mortality, morbidity, and life quality [1]. Despite remarkable advancements in multimodal treatments for HNSCC, including radical surgery, radiotherapy, molecularly targeted therapy, and immunotherapy, the long-term outcomes remain marginally improved [2]. Thus, it’s urgently needed to comprehensively unravel the molecular mechanisms underlying HNSCC tumorigenesis and identify promising targets for therapeutic intervention.

Long noncoding RNAs (lncRNAs), the transcripts exceeding 200 nucleotides with limited protein-coding potential, have been established as potent regulators involved in a myriad of cellular processes, including cell proliferation, apoptosis, migration, and invasion [3]. Large high-throughput genome-wide profiling assays have demonstrated that lncRNAs are aberrantly expressed in a cell/tissue-specific manner across human cancers, including HNSCC [4]. Moreover, accumulating evidence has established the pivotal regulatory roles of lncRNAs in tumor development through interactions with chromatin, DNA, regulatory proteins, and a variety of cellular RNA species [4]. Notably, they usually function as the competing endogenous RNAs (ceRNAs) or RNA sponges via interacting with miRNAs to fine-tune oncogene expression and, in turn, drive tumor initiation and progression [5, 6]. For example, lncRNA JPX decoyed miR-193b-3p to prevent PLAU mRNA degeneration, thereby upregulating PLAU and increasing epithelial–macrophage interaction to modulate malignant cell behaviors in HNSCC [7]. LncRNA LINC00173 adsorbed oncogenic miR-1275 to stabilize PROCA1 mRNA, which activated ZFP36L2-BCL2 to induce acquired cisplatin resistance in lung adenocarcinoma [8]. These findings highlight lncRNAs as key contributors to tumorigenesis and potential therapeutic targets for cancer therapy. LINC00973, a newly characterized lncRNA located at 3q12.1, has garnered growing interest due to its critical involvement in human malignancies. Markedly increased LINC00937 is significantly associated with unfavorable clinical outcomes in clear-cell renal cell carcinoma and non-small cell lung cancer [9, 10]. Furthermore, these prior studies suggested LINC00973 as a putative pro-tumorigenic noncoding RNA to modulate cancer cell proliferation and immune escape via acting as ceRNA [11]. However, the expression profile, biological roles, and relevant regulatory mechanisms of LINC00973 in HNSCC remain largely unexplored.

Super-enhancers (SEs), a specific class of cis-regulatory elements, are commonly defined as large clusters of enhancers spanning 10–50 kb of genomic DNA and characterized by extensive enrichments of H3K27ac, H3K4me1, and transcriptional co-factors (such as BRD4 and EP300), along with highly accessible chromatin [12]. They have been demonstrated as robust activators in modulating transcriptional programs implicated in various pathophysiological processes, particularly in tumorigenesis [13, 14]. A line of evidence has revealed that SE-driven transcriptional activation of oncogenic lncRNAs fuels tumor aggressive phenotypes and shapes the immune microenvironment to promote disease progression [15]. For example, epigenomic profiling of various types of squamous cell carcinomas revealed that SOX2/TP63 cooperatively and lineage-specifically regulated lncRNA CCAT1 expression through activation of its SEs and promoter to substantially promote cellular growth via activating MEK/ERK1/2 and PI3K/AKT signaling pathways [16]. Similar SE-driven lncRNA overexpression has been detected across multiple human cancers [17–19]. These findings collectively support SE-driven lncRNA dysregulation as a novel epigenetic hallmark of cancer, thus opening up new avenues to accelerate prognostic biomarker development and inform therapeutic interventions.

Herein, we identify LINC00973 as a novel SE-driven oncogenic lncRNA in HNSCC by integrative bioinformatics, epigenomics/transcriptomics profiling, cellular and molecular assays, and preclinical models. Mechanistically, AP-1/FOSL1 binds with LINC00973-SE and recruits BRD4, EP300, and RNA Pol II to activate its transcription; LINC00973 functions as a molecular sponge for miR-6756-3p, thereby activating its downstream target EN2 and NOTCH signaling to drive HNSCC progression. Our findings from preclinical models and clinical specimens further underscore LINC00973 as a promising prognostic biomarker and therapeutic target for HNSCC.

## Materials/subjects and methods

### Data acquisition

Pan-cancer (including HNSCC) transcriptomic data (TPM-normalized) and clinical information of HNSCC patients retrieved from The Cancer Genome Atlas (TCGA, RRID: SCR_003193). Additional bulk transcriptomic data from independent HNSCC cohorts (GSE41613, GSE42743, GSE65858, and GSE186775) as well as HNSCC single-cell transcriptomic matrices (GSE234933) were obtained from the Gene Expression Omnibus (GEO, RRID: SCR_005012). Chromatin immunoprecipitation sequencing (ChIP-seq) and in situ Hi-C followed by chromatin immunoprecipitation (HiChIP) for histone modification H3K27ac, Hi-C for OSCC samples, and FaDu cells, transcriptional coactivators (BRD4, EP300) and transcription factor (TF) (FOSL1), and transposase accessible chromatin with high-throughput sequencing (ATAC-seq) data were collected from either publicly available GEO repositories or generated in our previous studies [20]. Detailed accession numbers for all external datasets were provided in Supplementary Table S1.

### Cell culture, transfection, and chemicals

Cell lines including Cal27 (RRID: CVCL_1107) and HEK293T (RRID: CVCL_0063) were purchased from American Type Culture Collection (ATCC, USA). HN30 and human oral keratinocyte (HOK) were sourced from the State Key Laboratory Cultivation Base of Research, Prevention and Treatment for Oral Diseases, Nanjing Medical University. HN4 (RRID: CVCL_8127) and HN6 (RRID: CVCL_1107) were kindly gifted by Prof. Wantao Chen (Shanghai Jiaotong University). All cell lines were authenticated by short tandem repeat (STR) profiling. All cells were cultured in DMEM/F12 or DMEM (Invitrogen) with 10% FBS (Gibco, Thermo, USA) under conditions of constant temperature (37 °C) and humidity (5% CO_2_). Regular *mycoplasma* testing was performed throughout the study. Epigenetic inhibitors JQ1 (HY-13030) and NEO2734 (HY-136938) were purchased from MedChemExpress (USA), dissolved in DMSO, and administered at indicated concentrations. Lipofectamine 2000 (Thermo, USA) was utilized to perform cell transfection according to the manufacturer’s protocols.

### Plasmid, small interference RNAs (siRNAs), short hairpin RNAs (shRNAs), lentivirus, and miRNA constructs

Small interference RNAs (siRNAs) targeting human LINC00973, FOSL1, and EN2 were designed and synthesized by GenePharma (China). The most effective siRNA against LINC00973 (named as si-1) was subsequently subcloned into GV248 lentiviral vector (hU6-MCS-Ubiquitin-EGFP-IRES-puromycin) to generate a stable knockdown construct (named as sh-1). Cal27 and HN6 cells were infected with the sh-1 lentivirus in the presence of 6 μg/ml Polybrene and subsequently selected with 5 μg/ml puromycin for one week. Knockdown efficiency was validated by qRT-PCR and western blot. The detailed sequences of siRNA/shRNA were listed in Supplementary Table S2.

The N-3×Flag-EN2 plasmid was generated by subcloning human EN2 cDNA into the pcDNA3.1 vector. The LINC00973-MS2-12× plasmid was constructed by inserting human LINC00973 cDNA into the pSL-MS2-12× vector. The MS2-GFP plasmid was commercially obtained from Unibio (China). To create the LINC00973-SE enhancer fragment luciferase reporter plasmid, the core region of the E2 enhancer and non-binding region (E-NC) were cloned into the pGL3-Basic vector (named as pGL3-Linc00973-E2-luc and pGL3-Linc00973-E-NC-luc). The NOTCH1-specific pNOTCH1-TA-Luc luciferase reporter plasmid was purchased from Beyotime (China). The wild-type LINC00973 [LINC00973(WT)] and the wild-type 3’ untranslated region (UTR) of EN2 [EN2(WT)], along with their mutant counterparts harboring mutations in the potential miR-6756-3p binding sites [LINC00973(MT) and EN2(MT)], were cloned into the pmirGLO vector and sourced from GenePharma (China).

### Total/subcellular RNA extraction, quantitative reverse transcription-quantitative PCR (qRT-PCR)

Total RNA was isolated from cultured cells or clinical specimens following the established protocols from our previous study [21]. For mRNA detection, reverse transcription was performed using PrimeScript RT Master Mix Kit (RR036, TaKaRa, Japan), followed by qRT-PCR using ChamQ Universal SYBR qPCR Master Mix (Q312, Vazyme, China) on a Roche LightCycler 96 system (USA). For miRNA analysis, cDNA was synthesized using the miRNA 1st Strand cDNA Synthesis Kit (by stem-loop) (MR101, Vazyme, China), and qRT-PCR was carried out with miRNA Unimodal SYBR qPCR Master Mix (MQ102, Vazyme, China). Relative expression levels were calculated using the 2^−ΔΔCT^ method, with 18S RNA and U6 serving as endogenous controls for mRNA and miRNA, respectively. PARIS™ Kit (AM1921, Ambion, USA) was utilized to extract the cytoplasmic and nuclear RNA. Following extraction, the subcellular localization of LINC00973 was analyzed by qRT-PCR, with GAPDH and U6 serving as cytoplasmic and nuclear reference controls, respectively. The detailed primers for qRT-PCR were listed in Supplementary Table S3.

### Bulk RNA sequencing (RNA-seq)

For library construction, 3 μg of high-quality total RNA per sample was processed using NEBNext Ultra II RNA Library Prep Kit for Illumina (E7770, NEB, USA) according to the manufacturer’s protocol. Cluster generation was conducted on a cBot Cluster Generation System using TruSeq PE Cluster Kit v3-cBot-HS (Illumina, USA) according to the manufacturer’s guidelines. The prepared libraries were sequenced on an Illumina NovaSeq 6000 platform to generate 150 bp paired-end reads.

### Chromatin immunoprecipitation PCR (ChIP-qPCR)

For ChIP-qPCR assays, EZ-ChIP™ Chromatin Immunoprecipitation Kit (17–371, Millipore, USA) was employed according to the manufacturer’s instructions. Briefly, cells were crosslinked with 1% formaldehyde for 12 min, lysed to extract chromatin, and sonicated to shear DNA. Then, immunoprecipitation was performed overnight using ChIP-grade antibodies against H3K27ac (1:50, #8173, Cell signaling, USA), BRD4 (1:50, #13440, Cell signaling, USA), EP300 (1:50, #86377, Cell signaling, USA), FOSL1 (1:50, #5281, Cell signaling, USA) or normal mouse IgG (1:50, #68860, Cell signaling, USA). Detailed ChIP-qPCR primer sequences were listed in Supplementary Table S4.

### RNA immunoprecipitation (RIP)

RIP experiments were conducted by using the PureBinding® RNA Immunoprecipitation Kit (P0101, Geneseed, China) following the manufacturer’s protocol. For AGO2-RIP assays, 1 × 10^7^ cells were harvested and lysed in RIP lysis buffer supplemented with protease and RNase inhibitors. Cell lysates were incubated with anti-AGO2 antibody (0.5 μg, 67934-1-Ig, Proteintech, China) at 4 °C overnight with gentle rotation. Immunocomplexes were captured by pre-washed Protein A/G beads for 2 h at 4 °C. Co-precipitated LINC00973 RNA transcripts were extracted and quantified by qRT-PCR. For MS2-RIP assays, pSL-LINC00973-MS2-12× and MS2-GFP plasmids were co-transfected into Cal27, HN6, or HEK293T cells. Forty-eight hours post-transfection, cells were harvested and lysed under non-denaturing conditions. Immunoprecipitation was performed using an anti-GFP antibody (0.5 μg per sample, 50430-2-AP, Proteintech, China) to pull down the MS2-GFP fusion protein together with interacted LINC00973-MS2-12× transcripts and miRNAs. Then, RNA was isolated from the immunoprecipitates, and the relative enrichment of miR-6756-3p was assessed by qRT-PCR.

### Dual-luciferase reporter assay

Cal27 and HN6 were co-transfected pmirGLO-LINC00973, pmirGLO-LINC00973-mut and pmirGLO-EN2/pmirGLO-EN2-mut plasmids with/without miR-6756-3p mimics exposure for 48 h. Luciferase activity was measured using the Dual Luciferase Reporter Gene Assay Kit (G06001, GenePharma, China). Similar procedures were employed for pGL3-Linc00973-E2-luc and pNOTCH1-TA-Luc reporter assays.

### Fluorescence in situ hybridization (FISH) assay

The detailed protocol of the FISH assay has been described previously [21]. In brief, Cal27 and HN6 cells grown on coverslips were fixed with 4% paraformaldehyde for 10 min at room temperature, followed by permeabilization with 0.5% Triton X-100 for 15 min and pre-hybridized at 37 °C for 30 min. For detection the subcellular location of LINC00973, cells were hybridized with human LINC00973-specific Cy3-labeled FISH probe mixes (designed and synthesized by RiboBio, China) in hybridization buffer at 37 °C overnight. After sequential washes with saline sodium citrate buffer of decreasing concentrations, nuclei were counterstained with DAPI. Subcellular localization of LINC00973 was visualized and photographed using a laser scanning confocal microscope (LSM710, Zeiss, Germany).

### Western blot and co-immunoprecipitation (co-IP)

For Western blot, cells were lysed by Western & IP lysis buffer (#P0013, Beyotime, China) supplemented with a protease inhibitor cocktail at 4 °C. Protein concentration was determined using the Bradford method. Protein was separated on SDS-PAGE gels and transferred to PVDF membranes (Bio-Rad, China). Next, membranes were blocked with 5% non-fat dry milk and incubated overnight at 4 °C with the indicated primary antibodies: EN2 (1:500, CSB-PA007660LA01HU, Cusabio, China), N-cadherin (1:2000, #22018-1-AP, Proteintech, China), E-cadherin (1:20000, #20874-1-AP, Proteintech, China), Vimentin (1:20000, #10366-1-AP, Proteintech, China), GAPDH (1:50000, #60004-1-Ig, Proteintech, China), FOSL1 (1:1000, #5281, Cell signaling, USA), NOTCH1 (1:1000, #20687-1-AP, Proteintech, China), BRD4 (1:1000, #13440, Cell signaling, USA), HEY1 (1:1000, #19929-1-AP, Proteintech, China), N1ICD (1:1000, #4147, Cell signaling, USA) or Rpb1 (1:1000, #14958, Cell signaling, USA). After washing, membranes were incubated with HRP-conjugated secondary antibodies: Goat Anti-Mouse IgG (#RS0001, Immunoway, USA) or Goat Anti-Rabbit IgG (#RS0002, Immunoway, USA). Protein bands were visualized using Tanon High-sig ECL Western Blotting Substrate (180-5001, Tanon, China).

For co-IP assays, cell lysates were incubated with either FOSL1 (1:50, #5281, Cell signaling, USA) or mouse IgG (1:200, #68860, Cell signaling, USA) overnight at 4 °C. Protein-antibody complexes were captured with pre-balanced Protein A/G beads for 2 h at 4 °C. Immunoprecipitated proteins were measured by Western blot.

### Cell viability, migration, and invasion assays

Cell viability, cell migration, and invasion assays were performed as previously described [20]. In brief, cell viability was assessed using the Cell Counting Kit-8 (CCK-8, #A311, Vazyme, China) assay. Prior to these migration and invasion assays, cells were treated with DMEM/F12 media without fetal bovine serum (FBS) for 24 h to minimize the potential effects of cell proliferation on cell migration/invasion. Cell migration was evaluated by wound healing assays, while cell invasion was determined using Costar Transwell™ Permeable Supports (8.0-μm pore size, #3422, Corning, USA) coated with Basement Membrane Extract (#BME001, R&D, USA).

### EN2 mRNA stability detection

To evaluate EN2 mRNA stability, LINC00973 knockdown or control cells were seeded in 6-well plates and treated with actinomycin D (5 μg/mL, S8964, Selleck, USA) to inhibit transcription. Cells were harvested at 0, 2, 4, 6, and 8 h post-treatment. Total RNA was extracted, and EN2 mRNA levels were quantified by qRT-PCR.

### In vivo orthotopic xenograft model and patient-derived xenografts (PDX) model

The orthotopic floor-of-mouth xenograft model was employed to evaluate tumor growth and cervical lymph node metastasis of HNSCC in vivo, as previously described [22, 23]. Female BALB/c nude mice (6–8 weeks old) were maintained under specific pathogen-free (SPF) conditions with controlled temperature, humidity, and 12-h light/dark cycles and received tap water and forage ad libitum. All animal procedures were conducted in accordance with institutional guidelines and approved by the Institutional Animal Care and Use Committee (IACUC) of Nanjing Medical University (approve number: IACUC-2103011). Mice were randomly divided (*n* ≥ 6 per group), and LINC00973-knockdown (sh-1) with/without EN2 overexpression HN6 cells (1 × 10^5^ cells in 50 μl DMEM/Matrigel mixture per injection) were injected submucosally into the floor of the mouth using a 1-ml tuberculin syringe with a 30-gauge hypodermic needle. After 14 days, mice were humanely euthanized, and tumors as well as cervical lymph nodes were harvested for size measurement, histopathological, and immunohistochemical analysis to evaluate in vivo tumor growth and lymph node metastasis. The orthotopic tumor volumes were calculated as width^2^ × length/2. The number of lymph nodes with/without metastasis was measured by hematoxylin and eosin (H&E) and immunohistochemical staining. HNSCC PDX establishment and JQ1 or NEO2734 treatment were performed as we previously reported [24].

### Histological and immunohistochemical (IHC) analyses

H&E and IHC staining were performed according to standard protocols. For H&E staining, formalin-fixed paraffin-embedded tissues were sectioned at 4 μm thickness sections. Next, tissue sections were deparaffinized, rehydrated through graded alcohols, stained with hematoxylin for nuclear visualization, counterstained with eosin for cytoplasmic detail, and mounted with coverslips.

For IHC staining, deparaffinized sections underwent microwave-mediated antigen retrieval in citrate buffer, followed by treatment with 3% H_2_O_2_. After blocking with QuickBlock Blocking Buffer (#P0220, Beyotime, China), sections were incubated overnight at 4 °C with primary antibodies against CK5/6 (#MAB-0744, Maixin, China), Ki67 (#MAB-0672, Maixin, China), EN2 (1:100, #sc-293311, Santa Cruz, USA), FOSL1 (1:1000, #5281, Cell signaling, USA) or NOTCH1 (1:500, #20687-1-AP, Proteintech, China). Subsequently, sections were incubated with appropriate HRP-conjugated secondary antibodies, visualized using DAB, and counterstained with hematoxylin. Staining intensity was evaluated independently by two senior pathologists blinded to experimental conditions.

### Bulk RNA-seq data alignment

Raw RNA-seq reads were quality-trimmed and aligned to the human genome (hg19) using HISAT2 (version 2.2.1, RRID: SCR_015530). Aligned fragments were assigned to genes using the “featureCounts” command from the subread Python package (version 2.0.6, RRID: SCR_009803).

### ChIP-seq and Hi-ChIP data analysis, SE identification, and SE-associate gene annotation

ChIP-seq data alignment, SE identification and SE-associate gene annotation were conducted as previously described [12, 20]. For analysis of ChIP-seq data, raw fastq reads were aligned to the human reference genome (hg19) using the Bowtie2 algorithm (version 2.5.2, RRID: SCR_016368). The output BAM files were then sorted and indexed with SAMtools (version 1.18, RRID: SCR_002105), followed by duplicate read removal using Picard tools (version 3.0, RRID: SCR_006525). Peak calling was performed using MACS2 (version 2.2.9, RRID: SCR_013291) with a *q* value cutoff of 0.01.

SEs were identified using the custom-modified version of Rank Ordering of Super-Enhancers (ROSE, RRID: SCR_017390) algorithm (https://bitbucket.org/young_computation/rose/src/master/) with default parameters (stitching distance: 12.5 kb) [12]. The stitched H3K27ac peaks were ranked based on signal intensity, and SEs were defined as regions above the inflection point in the ranked distribution curve. For annotation the SE-associate gene, *ROSE_geneMapper.py* subcommand from the ROSE algorithm was utilized.

HiC-Pro was utilized to analyse HiChIP data and generate interaction matrices [25]. To generate anchor points for downstream looping analysis, outputs from HiC-Pro were used as inputs for peak calling in HiChIP-Peaks [26]. To ensure loops were called from similar background enhancers, peaks from HiChIP-Peaks were concatenated into a single file and used as anchor point inputs for loop calling via hichipper [27]. HiChIP loop visualization was performed using DNAlandscapeR (https://molpath.shinyapps.io/dnalandscaper/).

### Bulk/single-cell transcriptome data analyses

Differentially expressed lncRNAs were identified using the limma R package (version 3.60.2, RRID: SCR_010943). To assess prognostic significance, univariate and multivariate Cox regression analyses were performed with the survival R package (version 3.6.4, RRID: SCR_021137). Kaplan–Meier survival analyses were conducted to evaluate the association between LINC00973 expression and clinical outcomes, including overall survival (OS), disease-specific survival (DSS), and progression-free interval (PFI). Optimal cutoff values for LINC00973 expression were determined using the “surv_cutpoint” function (survminer R package, version 0.4.9, RRID: SCR_021094). Functional enrichment analysis was conducted through Kyoto Encyclopedia of Genes and Genomes (KEGG) pathway analysis and Gene Set Enrichment Analysis (GSEA) (clusterProfiler R package, version 4.12.0, RRID: SCR_016884). Additionally, Gene Set Variation Analysis (GSVA) was performed using the GSVA R package (version 1.52.2, RRID: SCR_021058), with gene sets obtained from the c2.cp.kegg.v7.4.symbols.gmt KEGG pathway set in MSigDB database.

For performing single-cell RNA-seq (scRNA-seq) analysis, the expression matrices were processed using the Seurat R package (version 3.1.0, RRID: SCR_016341). After quality filtering, data were normalized, scaled and visualized using standard Seurat workflows. Dimensionality reduction was performed via principal component analysis (PCA) and uniform manifold approximation and projection. Cell populations were manually annotated based on canonical marker genes.

### CRISPR-mediated enhancer repression

Cal27 and HN6 cells were transfected with pLV[Exp]-CBh-dCas9-KRAB-MeCP2:T2A:Hygro (VectorBuilder, China) lentivirus plasmid for 12 h, recovered for 72 h and underwent selection with hygromycin B (200 μg/mL, HY-B0490, MedChemExpress) for another 7 days [28]. sgRNAs were designed to target E1-E4 on the GPP CRISPick webtool (https://portals.broadinstitute.org/gppx/crispick/public) and then synthesized by GenScript (China). The detailed sgRNA sequences used were listed in Supplementary Table S5. A final concentration of 200 nM sgRNAs was electroporated into Cal27/HN6-dCas9-KRAB cells via BTX GEMINI System (BTX Technologies, USA). Forty-eight hours later, these cells were collected for qRT-PCR to detect expression changes of LINC00973.

### Motif analyses

Putative TF binding sites within the LINC00973-SE region were identified through de novo motif discovery using HOMER software (version 4.10, RRID: SCR_010881). The analysis employed the *findMotifsGenome.pl* script with standard parameters (-size 200 -len 8, 10, 12). Motif matrices were compared against the comprehensive JASPAR 2022 CORE vertebrates database. Results were sorted by statistical significance (Benjamini-Hochberg adjusted *P* values) and represented as consensus binding sequence logos.

### In-house HNSCC cohort and primary sample collection

Fifty paired HNSCC and adjacent nontumor mucosal tissues were collected from patients receiving radical surgical resection at the Department of Oral and Maxillofacial Surgery, Affiliated Stomatological Hospital, Nanjing Medical University between April 2016 to December 2021. Inclusion criteria required: 1. histologically confirmed primary HNSCC diagnosis verified by two independent senior pathologists; 2. no prior treatment received. Tissue specimens were immediately snap-frozen in liquid nitrogen and stored at −80 °C until use. Clinicopathological information, including longitudinal follow-up outcomes, age, gender, TNM staging, smoking history, and alcohol consumption, was documented. The study protocol was conducted in accordance with the Declaration of Helsinki, with written informed consent obtained from all participants. The whole study was approved by the Clinical Research Ethics Committee of Affiliated Stomatological Hospital of Nanjing Medical University (approve number: PJ2016-021-001 and PJ2021-030-001).

### Statistical analyses

All quantitative results are presented as mean ± SD deviation from at least three independent experiments. The comparative analyses were executed in GraphPad Prism 9.0 (RRID: SCR_002798), utilizing Student’s *t* test for pairwise comparisons or one-way ANOVA for multi-group evaluations as statistically warranted. Contingency analyses of LINC00973 expression patterns against clinicopathological variables employed either χ² test or Fisher’s exact probability test. Survival was compared via Kaplan–Meier method with log-rank test. The thresholds for statistical significance were established at ^#^*P* ≥ 0.05, *P < 0.05 and **P < 0.01.

## Results

### LINC00973 is aberrantly upregulated and associates with unfavorable clinical outcomes in HNSCC

**Fig. 1:**
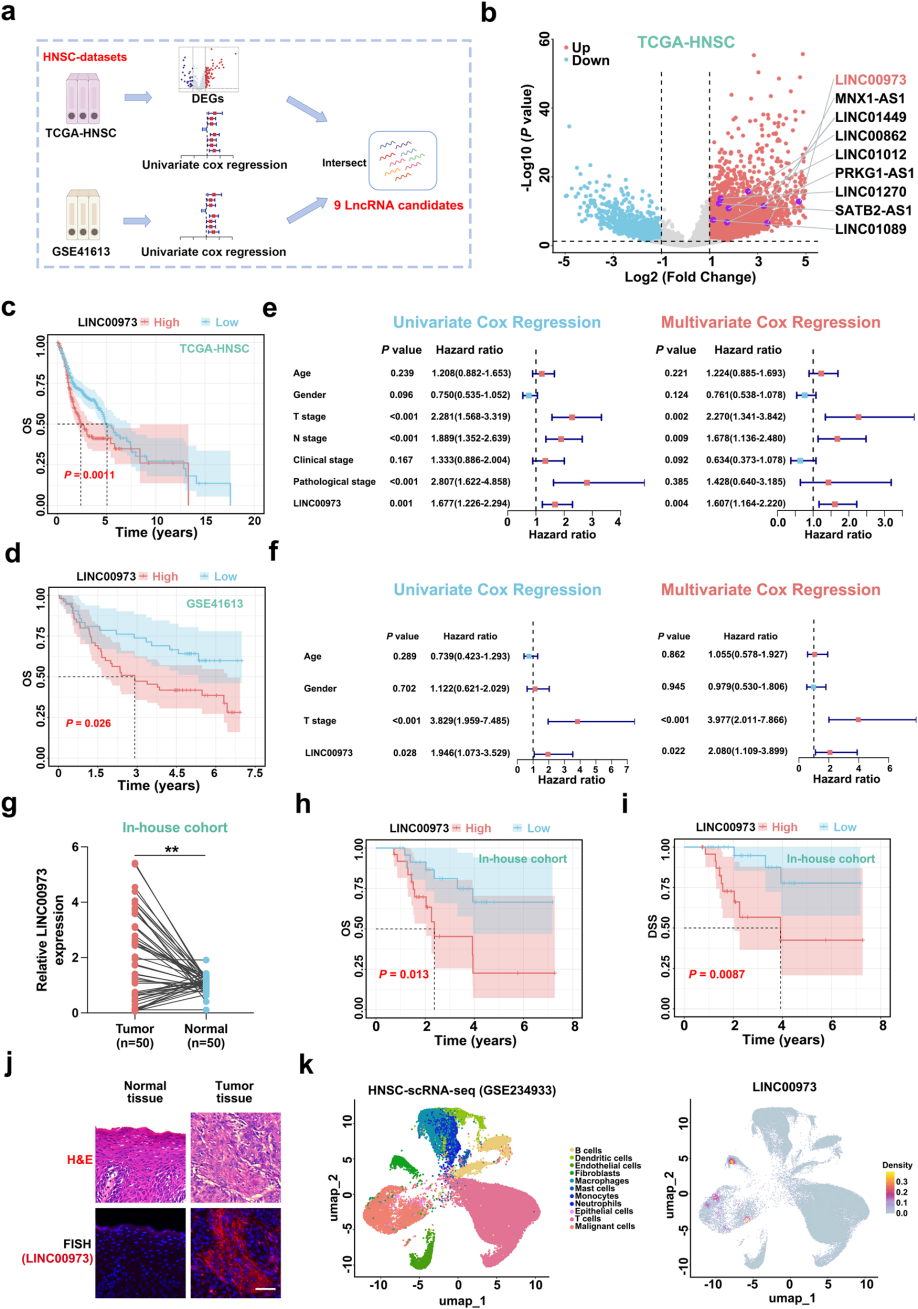
LINC00973 is aberrantly upregulated and associates with unfavorable clinical outcomes in HNSCC. **a** Schematic diagram depicted the experimental workflow design to identify lncRNA candidates with prognostication in HNSCC. **b** Volcano plot displayed the differentially expressed lncRNAs profiling in the TCGA-HNSC datasets, and nine upregulated lncRNA candidates with significant prognostic values were marked respectively. **c**, **d** Survival analyses for overall survival (OS) in subgroups with high or low LINC00973 abundance were determined and illustrated by Kaplan–Meier plots in TCGA-HNSC and GSE41613 datasets. The *surv_cutpoint* function in “survminer” R package were utilized to calculate the best cutoff point. Log-rank test. **e**, **f** LINC00973 RNA levels were significantly correlated with patients’ survival in TCGA-HNSC and GSE41613 cohort by both univariate and multivariate Cox regression analyses. **g** The expressions of LINC00973 in 50 HNSCC samples and paired normal counterparts were assessed by qRT-PCR and compared. Paired Wilcoxon rank-sum test. **P < 0.01 **h**, **i** Kaplan–Meier plots of OS and disease-specific survival (DSS) in subgroups with high/low LINC00973 expression in our in-house cohort (median value of LINC00973 as cutoff). Log-rank test. **j** Representative photographs of FISH assay incorporated with H&E staining in our paired samples. The FISH probes of LINC00973 were labeled with Cy3 (red), and nuclei were stained with DAPI (blue). Scale bar: 50 μm. **k** Data analysis and cell type annotations of HNSCC scRNA-seq dataset (GSE234933) identified 11 main cell types (left panel). Followed uniform manifold approximation and projection (UMAP) displayed a preferential LINC00973 expression in malignant epithelial cells (right panel).

Given the pro-tumorigenic roles of lncRNAs during tumor progression, we first leveraged differentially expression analysis in TCGA-HNSC dataset to characterize the aberrant lncRNA landscape and dysregulated lncRNA candidates. As shown in Fig. 1a, b, we identified 4369 lncRNAs (Log_2_(Fold change)≥1, *P* < 0.05) overexpressed in HNSCC samples. To prioritize lncRNAs with prognostic significance, we performed univariate Cox regression analysis on these upregulated lncRNAs across two independent HNSCC datasets (TCGA-HNSC and GSE41613). This approach yielded 568 prognostic lncRNAs in the TCGA-HNSC dataset and 210 in GSE41613, with 9 overlapping prognostic lncRNAs (Fig. 1b). Notably, LINC00973 emerged as the top candidate with marked upregulation in HNSCC. The aberrant overexpression of LINC00973 was detected across multiple non-HNSCC cancer types in TCGA-Pancer dataset (Supplementary Fig. S1a). In addition, we exploited two independent prediction algorithms, Coding Potential Calculator 2 and LncFinder, to confirm the noncoding nature of LINC00973 based on the RNA sequence intrinsic features (Supplementary Fig. S1b, c) [29, 30]. Furthermore, survival analyses indicated that elevated LINC00973 expression positively correlated with reduced OS, DSS, and PFI across multiple cohorts (Fig. 1c, d and Supplementary Fig. S2a, b). Univariate and multivariate Cox regression analyses confirmed LINC00973 abundance as an independent prognostic factor in HNSCC after adjustments of multiple clinicopathological parameters (Fig. 1e, f and Supplementary Fig. S2c, d). Consistently, abnormal upregulation of LINC00973 was detected in our in-house cohort samples (including 50 paired HNSCC and adjacent nontumor tissues). Patients with elevated LINC00973 expression experienced worse OS, DSS, and higher clinical stages (Fig. 1g–i and Table 1).

**Table 1:** LINC00973 expression and their associations with clinicopathological parameters in 50 patients with HNSCC.

Clinical parameter	Quantity	LINC00973 expression		*P* value
		High	Low	
Age				0.57
<60	23	10	13	
≥60	27	15	12	
Smoking				0.76
No	34	16	18	
Yes	16	9	7	
Alcohol use				1.00
No	38	19	19	
Yes	12	6	6	
Gender				0.25
Male	29	17	12	
Female	21	8	13	
T stage				0.24
I–II	31	13	18	
III–IV	19	12	7	
N stage				0.37
N0	32	14	18	
N+	18	11	7	
Clinical stage				**0.04**
I–II	20	6	14	
III–IV	30	19	11	

The number in bold indicate statistical significance with *P* values less than 0.05. Fisher’s exact test.

Differences were considered statistically (bold values) significant at *P* < 0.05.

To characterize the cellular distribution of LINC00973 within the HNSCC tumor ecosystem, we performed RNA FISH staining on clinical specimens and revealed predominant LINC00973 expression within cancer cells in samples (Fig. 1j). In line with this, single-cell transcriptome data analysis of HNSCC samples (GSE234933) confirmed preferential enrichment of LINC00973 transcript in malignant epithelial cells (Fig. 1k). Collectively, these data reveal that LINC00973 represents a putative pro-oncogenic lncRNA with prognostic significance in HNSCC, thus warranting further in-depth explorations of its biological functions and mechanisms of action.

### LINC00973 drives HNSCC malignant phenotypes in vitro and vivo

**Fig. 2:**
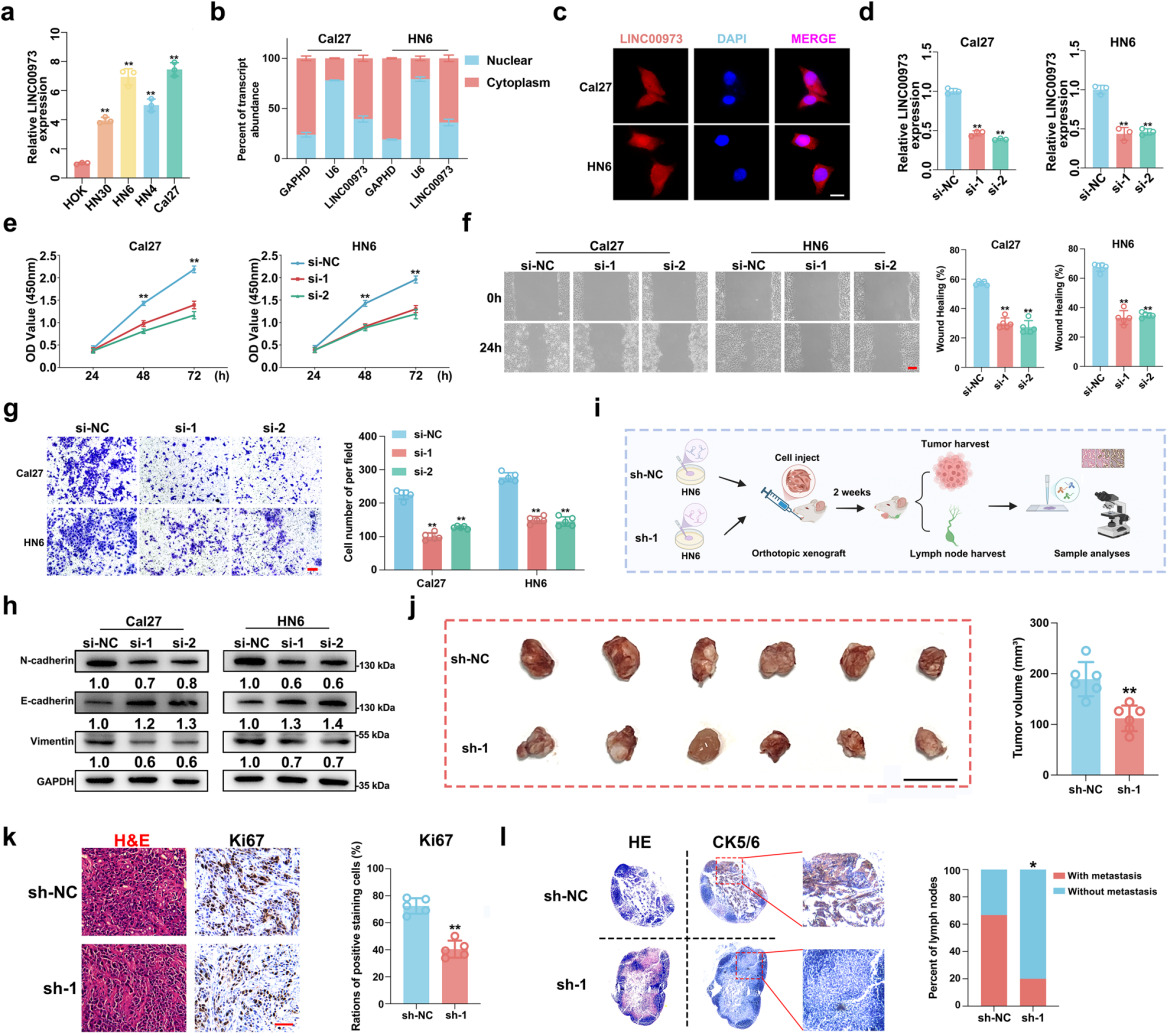
LINC00973 drives HNSCC malignant phenotypes in vitro and vivo*.* **a** The relative expression of LINC00973 in normal human oral keratinocytes (HOK) cell and HNSCC cell lines were measured by qRT-PCR. **b** Subcellular RNA fraction and followed qRT-PCR assays were conducted to determine the localization of LINC00973 within Cal27 and HN6 cells. GAPDH and U6 served as the internal control of cytoplasm and nuclear, respectively. **c** RNA FISH assays showed LINC00973 mainly localized in cytoplasm in Cal27 and HN6 cells. Scale bar: 10 μm. **d** The knockdown efficiency of LINC00973 was determined by qRT-PCR in Cal27 and HN6 cells transfected si-LINC00973-1 (si-1) and si-LINC00973-2 (si-2). Cell proliferation, migration, and invasion was evaluated by CCK-8 (**e**), wound-healing (**f**), and Transwell invasion assays (**g**) in LINC00973-sliencing Cal27 and HN6 cells. Scale bar: 50 μm. **h** The protein abundance of indicated migration and invasion related markers was detected in LINC00973-deficient cells. **i** Schematic description of experimental procedures for the orthotopic floor-of-mouth xenograft model. **j** Representative image of the tumor masses with stable LINC00973 knockdown/control HN6 cells and the final volume of samples were recorded. Scale bar: 1 cm. **k** Representative IHC staining of Ki67 in xenograft tumors (left panel) and their quantification data were shown (right panel). Scale bar: 50 μm. **l** Representative H&E staining and IHC staining of CK5/6 in lymph nodes (left panel) from orthotopic tumor-bearing mice. The percentage of lymph nodes metastasis were shown and compared (right panel). Fisher’s exact test. Data shown here are mean ± SD from three independent experiments, **P < 0.01, Student’s *t* or one-way ANOVA test.

Next, we set out to determine the biological roles of LINC00973 in HNSCC by siRNA-mediated loss-of-function assays. Initially, we measured the LINC00973 levels across a panel of HNSCC cell lines and found Cal27 and HN6 with the highest baseline expression (Fig. 2a). Subcellular RNA extraction followed qRT-PCR and FISH analyses both indicated the predominant cytoplasmic localization of LINC00973 in Cal27 and HN6 cell lines (Fig. 2b, c). Then, we designed and synthesized two independent siRNAs targeting human LINC00973 to measure phenotypic changes after LINC00973 knockdown. As shown in Fig. 2d–g and Supplementary Fig. S3, LINC00973 depletion significantly suppressed cell proliferation, migration and invasion and induced cell apoptosis, cell cycle arrest and senescence in vitro. In contrast, enforced LINC00973 overexpression markedly induced cell proliferation, migration and invasion in vitro, well consistent with those findings from LINC00973 knockdown (Supplementary Fig. S4). Expression changes of selected markers like N-cadherin, E-cadherin and Vimentin partially supported the impaired migrative and invasive capacities upon LINC00973 silencing (Fig. 2h). To validate these findings in vivo, we established a HNSCC orthotopic xenograft model using HN6 cells with stable LINC00973 knockdown (sh-1). Two weeks post-transplantation, both primary tumors and cervical lymph nodes were harvested for analyses (Fig. 2i). Tumor masses derived from HN6-sh-1 cells exhibited significantly reduced volumes and Ki67 expression, indicative of impaired cell proliferation and tumor growth in vivo (Fig. 2j, k). In particular, animals bearing with HN6-sh-1 cells had substantially lower rates of cervical lymph node metastasis as measured by CK5/6 immunostaining in those harvested nodes (Fig. 2l). Complementarily, we performed differentially expression analysis in TCGA-HNSC samples stratified by top and bottom 100 samples with LINC00973 expression (designated as LINC00973^high^ or LINC00973^low^). GSEA analyses based on differentially expression genes (DEGs) revealed that LINC00973^high^ samples were significantly enriched in cancer-related pathways, including NOTCH signaling and epithelial-mesenchymal transition (EMT) (Supplementary Fig. S5). In aggregate, our results strongly favor that LINC00973 functions as a putative oncogene via promoting cell proliferation, migration and invasion, thus collectively facilitated HNSCC progression.

### EN2 is identified as the key downstream effector of LINC00973 to facilitate HNSCC tumorigenesis

**Fig. 3:**
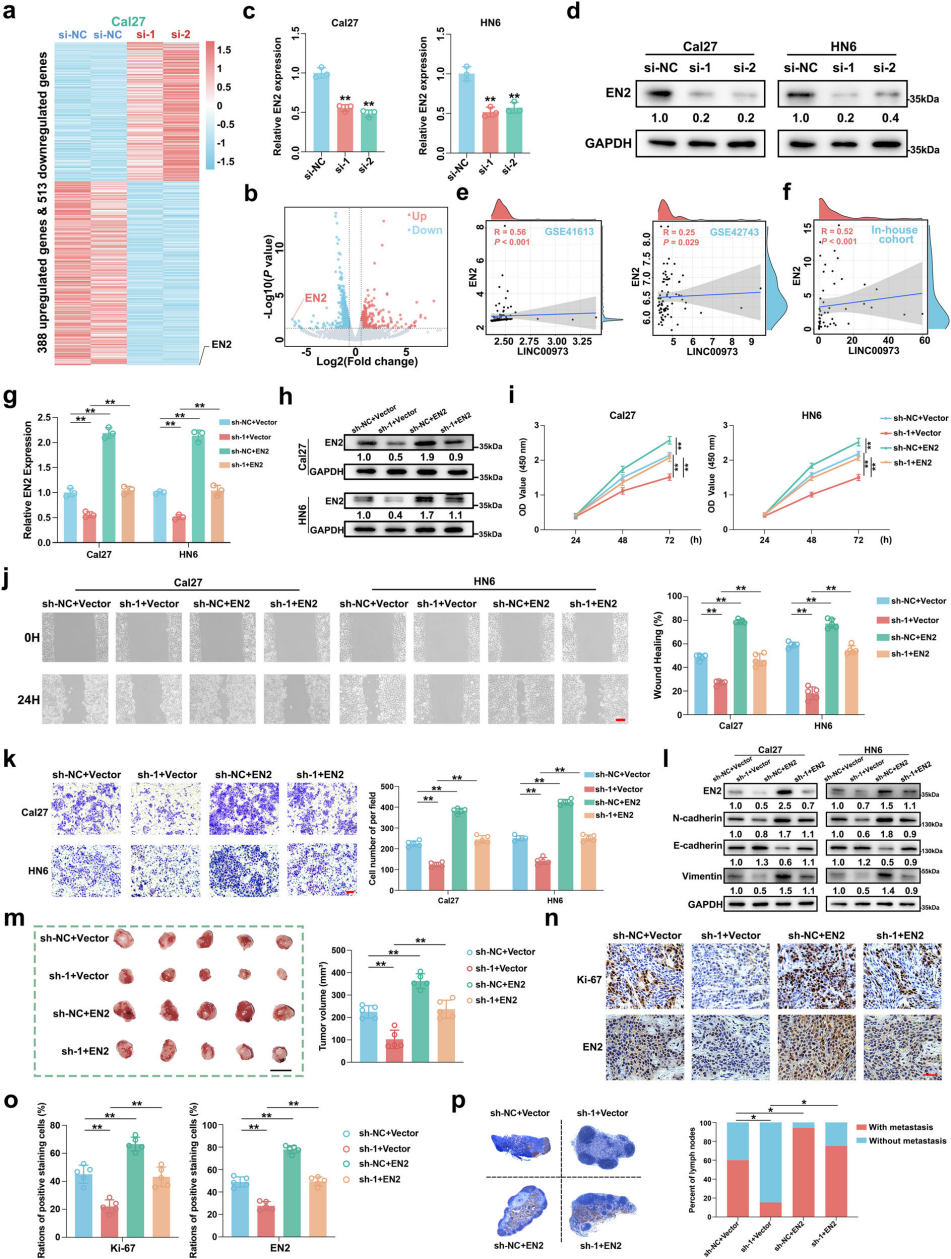
EN2 is identified as the key downstream effector of LINC00973 to facilitate HNSCC tumorigenesis. Heatmap (**a**) and volcano plot (**b**) displayed the expression profile of DEGs following LINC00973 knockdown or control cells. The mRNA (**c**) and protein (**d**) expression of EN2 were measured in Cal27 and HN6 cells with/without EN2 knockdown. **e**, **f** The correlations between EN2 and LINC00973 RNA expression were assessed in the public HNSCC datasets (GSE41613 and GSE42743) and our in-house samples. Spearman’s correlation. The mRNA (**g**) and protein (**h**) abundance of EN2 were measured in Cal27 and HN6 cells under four conditions. Cell proliferation, migration and invasion were significantly reduced following LINC00973 knockdown but restored by ectopic EN2 overexpression as gauged by CCK-8 (**i**), wound healing (**j**) and Transwell invasion assays (**k**). Scale bar: 50 μm. **l** The migration/invasion-related markers were determined by western blot in Cal27 and HN6 cells with/without LINC00973 knockdown coupled with/without EN2 overexpression. **m** 1 × 10^5^ HN6 cells with LINC00973 or/and EN2 manipulations were injected submucosally into the floor of the mouth. Fourteen days post-injection, tumor masses and cervical lymph nodes were harvested. Representative tumor masses images and estimated tumor volumes were showed. Scale bar: 1 cm. Representative IHC staining of Ki67 and EN2 in xenograft tumors (**n**) and their quantification data were shown (**o**). Scale bar: 50 μm. **p** Representative IHC staining of CK5/6 in lymph nodes (left panel) from orthotopic tumor-bearing mice among four indicated groups were displayed. The percentage of lymph nodes metastasis were shown and compared (right panel). Fisher’s exact test. Data were presented as mean ± SD, **P < 0.01, Student’s *t* test.

Next, we sought to identify potential downstream effectors regulated by LINC00973 by RNA-seq following LINC00973 knockdown in HNSCC cells. A total number of 901 altered genes (388 upregulated, 513 downregulated; *P* < 0.05, |Log_2_(Fold change)| > 0.585) were detected after LINC00973 knockdown, which were significantly enriched in cancer-associated pathways, including PI3K-Akt and MAPK pathways (Fig. 3a and Supplementary Fig. S6). Notably, EN2 emerged as the most downregulated gene following LINC00973 silencing (Log_2_(Fold change) = −6.2, Fig. 3b). Multiple lines of evidence have indicated that EN2 functions as a putative oncogene across various cancer types, including bladder, breast, and colorectal cancers [31–33]. Thus, we selected EN2 as a potential LINC00973 downstream target for further experimental validation. Initial bulk pan-cancer transcriptome data analyses have revealed elevated EN2 expression in diverse cancer contexts, including HNSCC, and subsequent HNSCC scRNA-seq data re-analyses unveiled its predominant expression in malignant epithelial cells (Supplementary Fig. S7a–c). In addition, EN2 transcriptional abundance was significantly increased in our HNSCC samples and correlated with inferior OS and DSS (Supplementary Fig. S7d, e). DEG and GSEA analyses in the TCGA-HNSC dataset stratified by EN2 expression indicated that EN2^high^ samples were enriched in multiple oncogenic categories, including JAK-STAT3 signaling and E2F targets (Supplementary Fig. S7f). Both qRT-PCR and western blot analyses confirmed that LINC00973 knockdown substantially reduced EN2 expression at both mRNA and protein levels (Fig. 3c, d). Importantly, we found significant positive associations between LINC00973 and EN2 expression across multiple datasets (GSE41613 and GSE42743) and our in-house cohort (Fig. 3e, f).

To verify whether EN2 functioned as a key downstream effector of LINC00973 in HNSCC, we next performed rescue experiments by overexpressing EN2 in LINC00973-depleted cells (Fig. 3g, h). Ectopic EN2 overexpression effectively abrogated LINC00973 knockdown-mediated inhibitory effects on cell proliferation, migration, and invasion in vitro (Fig. 3i–l). Noticeably, enforced EN2 overexpression potently attenuated the impaired tumor growth induced by LINC00973 knockdown and enhanced cancer lymph node metastasis in the orthotopic xenograft model (Fig. 3m–p). Taken together, these findings strongly establish EN2 as a critical downstream effector of LINC00973 to fuel HNSCC progression.

### LINC00973 adsorbs miR-6756-3p to attenuate EN2 mRNA degeneration in HNSCC

**Fig. 4:**
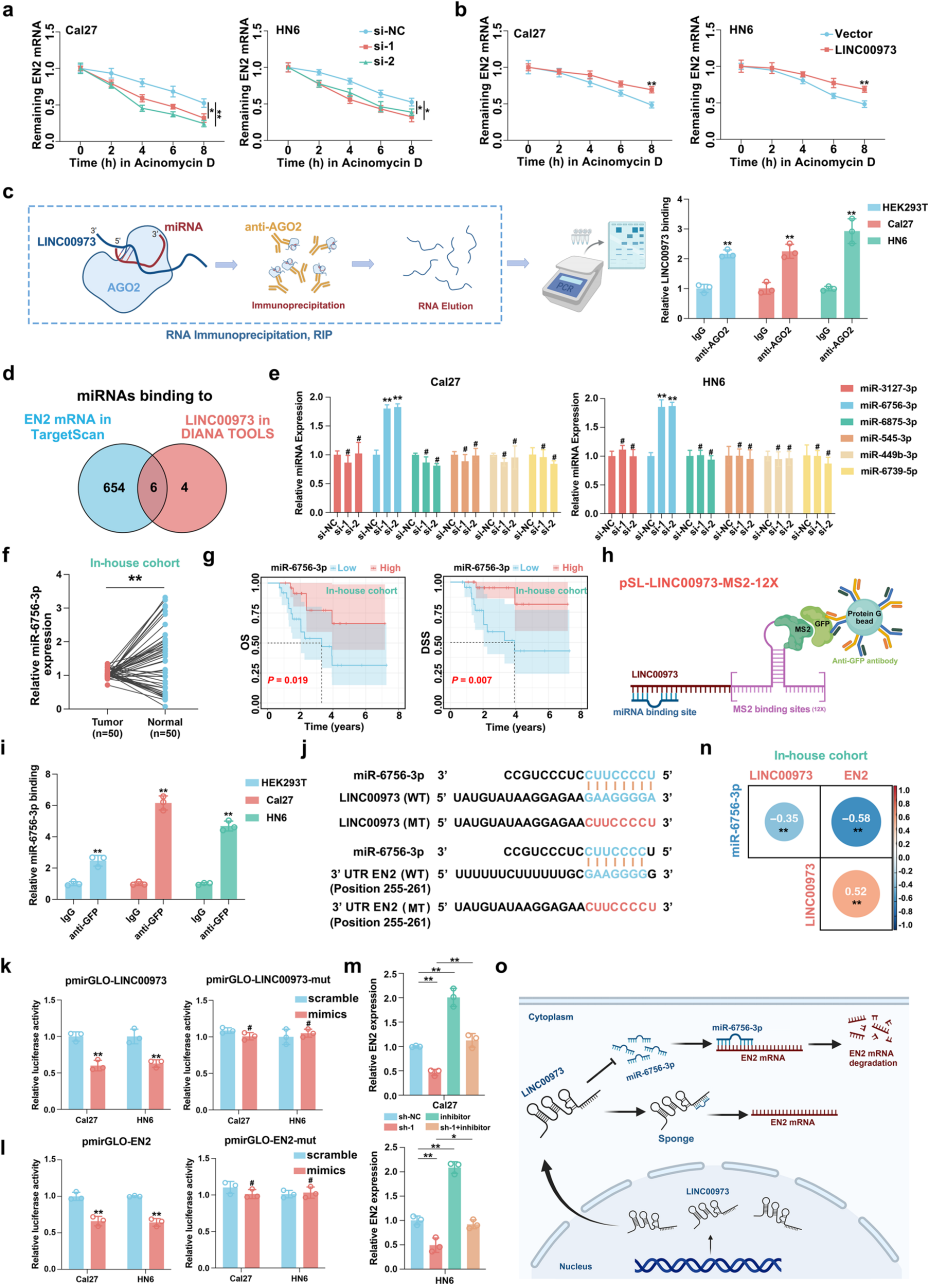
LINC00973 adsorbs miR-6756-3p to attenuate EN2 mRNA degeneration in HNSCC. **a**, **b** The half-life of EN2 mRNA were determined by qRT-PCR in LINC00973 knockdown or overexpressed Cal27 and HN6 cells with time-course Actinomycin D exposure. **c** Experimental design of AGO2-RIP was depicted by schematic diagram (left panel). Endogenous LINC00973 was assessed by qRT-PCR in cell lyses immunoprecipitated with either AGO2 antibody or mouse IgG (right panel). **d**, **e** The putative miRNA candidates with high EN2 mRNA binding affinity were computed by TargetScan webtool (https://www.targetscan.org/vert_80/), while LINC00973 sponged miRNAs (top10) were predicted by DIANA tool kits (http://diana.imis.athena-innovation.gr/DianaTools/index.php). Six overlapped miRNA candidates were identified (**d**). Only miR-6756-3p expression were shown with significant upregulation followed by LINC00973 silencing in both Cal27 and HN6 cells (**e**). **f** The expression of miR-6756-3p in 50 HNSCC clinical samples and paired normal counterparts were determined by qRT-PCR. Paired Wilcoxon rank-sum test. **g** Kaplan-Meier analyses of OS and DSS stratified by median miR-6756-3p expression in 50 in-house samples Log-rank test. **h**, **i** Schematic diagram depicted the experimental design of MS2-RIP assays to detect the direct binding between LINC00973 and miR-6756-3p in HNSCC (**h**). The LINC00973-MS2-GFP complex was pulled down by anti-GFP antibody and the interacted miR-6756-3p were assessed by qRT-PCR in Cal27, HN6, and HEK293T cells (**i**). **j** The sequence alignment displayed the binding sequence between miR-6756-3p and LINC00973 [LINC00973 (WT)] or 3’UTR of EN2 [3’UTR EN2 (WT)]. The miR-6756-3p binding site mutated LINC00973 and EN2 transcripts [LINC00973 (MT) or 3’UTR EN2 (MT)] were showed below. **k**, **l** Cal27 and HN6 cells transfected with LINC00973/3’UTR EN2 wild type or miR-6756-3p binding site mutated pmirGLO luciferase reporter vectors and treated with miR-6756-3p or control mimics. The luciferase activated was measured. **m** The expression levels of EN2 were measured by qRT-PCR in Cal27 and HN6 treated with sh-LINC00973-1 lentivirus or in combination with miR-6756-3p inhibitor (inhibitor). **n** The correlations between EN2, LINC00973, and miR-6756-3p expression in HNSCC clinical samples were estimated by using Spearman’s correlation. **o** Schematic illustration of the regulatory model of LINC00973 decoys miR-6756-3p to stabilize EN2 mRNA. Data were presented as mean ± SD, ^#^*P* ≥ 0.05, *P < 0.05, **P < 0.01, Student’s *t* test.

Mounting evidence has established that the subcellular distribution of lncRNAs largely governs their biological activities under diverse contexts [34]. Given the predominant cytoplasmic localization of LINC00973 and its positive regulation of EN2 in HNSCC cells, we hypothesized that LINC00973 might competitively bind miRNAs to prevent EN2 mRNA decay [10, 11]. Initially, we measured EN2 mRNA half-life following LINC00973 depletion or overexpression in vitro and confirmed that LINC00973 was capable to stabilize EN2 mRNA (Fig. 4a, b). Next, we performed AGO2-RIP assays and found that LINC00973 was successfully pulled down by anti-AGO2 antibody but not control IgG in Cal27, HN6 and HEK293T cells, strongly suggesting its potential role as miRNA sponge (Fig. 4c). Then we leveraged two algorithms TargetScan and DIANA TOOLS to screen miRNAs that interacted with both LINC00973 and EN2 mRNA and found 6 overlapped miRNA candidates (Fig. 4d). However, only miR-6756-3p exhibited significant upregulation following LINC00973 silencing (Fig. 4e). Reduced miR-6756-3p expression was detected and predicted inferior OS and DSS in samples from our in-house cohort (Fig. 4f, g**)**. To validate the direct interaction between LINC00973 and miR-6756-3p, we performed MS2-based RIP assays using cells co-transfected with MS2-GFP fusion protein and LINC00973 tagged with 12 × MS2 binding sequences plasmids. As shown in Fig. 4h, i, these results indicated that miR-6756-3p directly bound with LINC00973 in these three cell lines examined.

Next, we sought to explore the molecular interplay between miR-6756-3p and LINC00973/EN2 in detail. Two complementary approaches to manipulate endogenous miR-6756-3p levels by mimics or inhibitor oligonucleotides and determine the resulting changes of EN2 mRNA were exploited. As expected, introductions of miR-6756-3p mimics potently repressed EN2 mRNA expression and reduced its half-life, while the inhibitor executed the opposite effects (Supplementary Fig. S8). To experimentally validate their direct interactions, we constructed luciferase reporters containing wild-type or mutated binding sites for miR-6756-3p within LINC00973 and EN2 3’UTR sequences (Fig. 4j). Luciferase assays indicated that miR-6756-3p significantly suppressed the luciferase activities of wild-type LINC00973 and EN2 reporters, and had negligible effects on reporters with mutated binding sites (Fig. 4k, l). Moreover, miR-6756-3p inhibition effectively rescued EN2 downregulation induced by LINC00973 knockdown, thus at least in part confirming miR-6756-3p as the key mediator involved in LINC00973 regulatory effects on EN2 (Fig. 4m). Importantly, the miR-6756-3p expression was markedly negatively correlated with LINC00973 and EN2 abundance in clinical specimens from our in-house cohort (Fig. 4n). Moreover, we performed rescue experiments by inhibiting miR-6756-3p in LINC00973-depleted cells (Supplementary Figure S9a). miR-6756-3p inhibition effectively abrogated LINC00973 knockdown-mediated inhibitory effects on cell proliferation, migration, and invasion in vitro (Supplementary Fig. S9b-d). Taken together, we conclude that LINC00973 functions as a ceRNA by competitively binding miR-6756-3p, which in turn releases EN2 mRNA from miRNA-mediated degeneration and promotes tumor progression in HNSCC (Fig. 4o).

### AP-1/FOSL1 promotes SE-mediated LINC00973 transcription in HNSCC

**Fig. 5:**
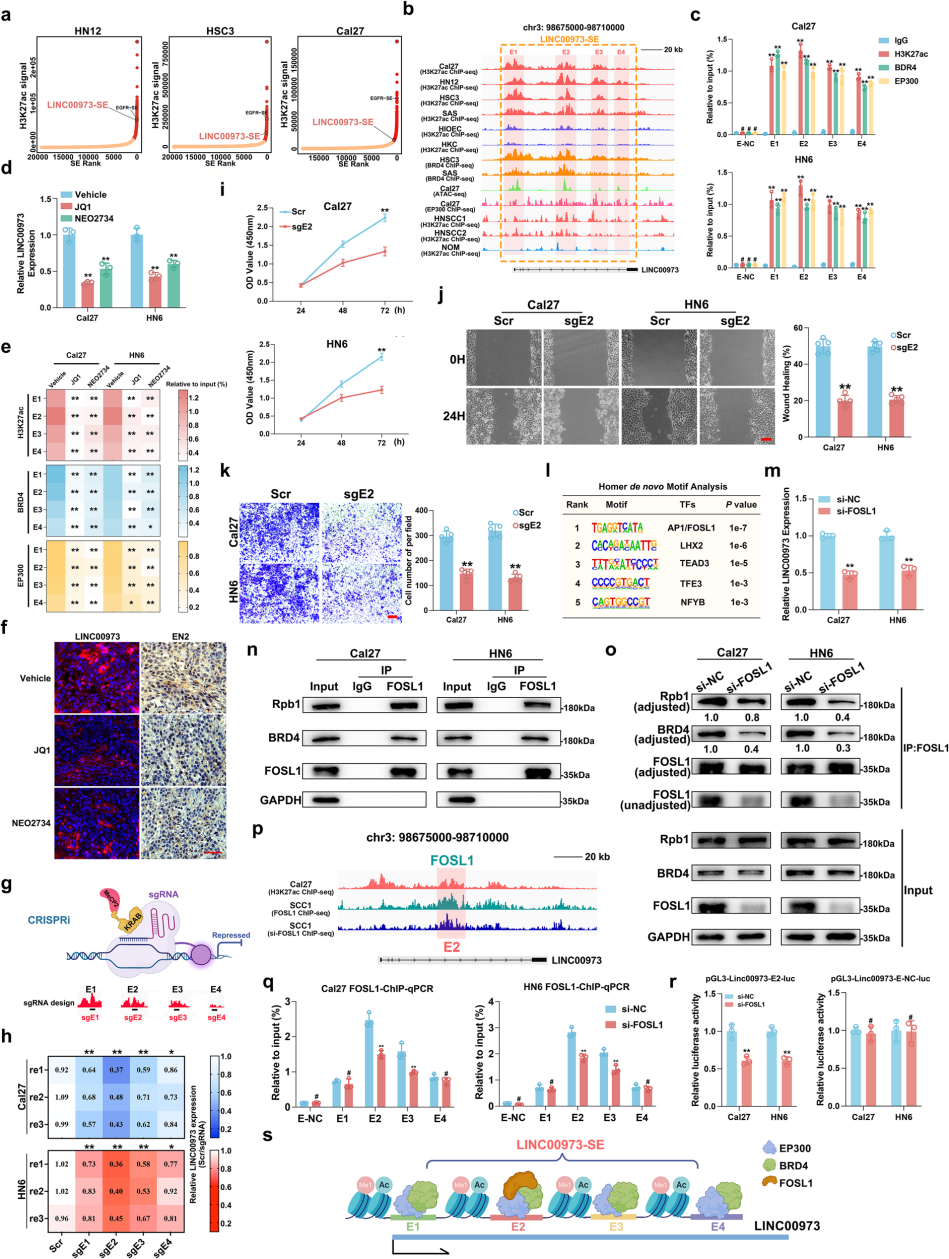
AP-1/FOSL1 promotes SE-mediated LINC00973 transcription in HNSCC. **a** SEs were calculated and ranked by H3K27ac signal using ROSE algorithm. And a proximal SE within LINC00973 gene locus were identified in 3 HNSCC cell lines (Cal27, HN12, and HSC3). **b** Genomic tracks plot displayed the H3K27ac enrichment (including 6 cell lines, 2 primary HNSCC and 1 human normal oral mucosa (NOM) tissues), BRD4, and EP300 binding and chromatin accessibility at the LINC00973-SE region. Four individual enhancer fragments within LINC00973-SE were observed and referred as E1-E4. **c** The H3K27ac, BRD4 and EP300 enrichment on E1-E4 or E-NC were measured by ChIP-qPCR in Cal27 and HN6 cells. **d**, **e** The expression changes of LINC00973 were measured by qRT-PCR and the binding changes of H3K27ac, BRD4, and EP300 on E1-E4 were determined by ChIP-qPCR in Cal27 and HN6 cells treated with JQ1 (1 μM, 12 h) or NEO2734 (1 μM, 12 h). **f** Representative FISH staining of LINC00973 and IHC staining of EN2 in PDX samples treated with vehicle, JQ1 or NEO2734. **g** Schematic diagram showing the procedure of E1-E4 repression by dCas9-KRAB-MeCP2 system. **h** LINC00973 expression in Cal27-dCas9 and HN6-dCas9 cells with repressed LINC00973 enhancer activity were assessed by qRT-PCR. Cell proliferation, migration and invasion were significantly suppressed following sgE2 treatment as gauged by CCK-8 (**i**), wound healing (**j**) and Transwell invasion assays (**k**). Scale bar: 50 μm. **l** De novo motif discovery at LINC00973-SE regions via HOMER algorithm. **m** LINC00973 expression was detected by qRT-PCR in Cal27 and HN6 cell with/without FOSL1 silencing. **n**, **o** co-IP assays revealed the endogenous FOSL1 protein interacted with Rpb1 (RNA pol II subunit) and BRD4 in both Cal27 and HN6 cells (**i**), while FOSL1-depletion disrupted these interactions (**j**). **p** The FOSL1 binding enrichments at E2 region in SCC1 with/without FOSL1 knockdown were displayed by track plot. **q** FOSL1 binding on E1-E4 or E-NC regions in Cal27 and HN6 transfected with si-FOSL1 were measured by ChIP-qPCR. **r** The core DNA sequences of E2 or E-NC were subcloned into pGL3 vectors and the luciferase activities were detected in Cal27 and HN6 cells transfected with si-FOSL1 or control siRNAs. **s** Schematic model of LINC00973 transcription regulation: *cis*-activation by LINC00973-SE and *trans*-regulation by AP-1/FOSL1. Data were presented as mean ± SD, ^#^*P* ≥ 0.05,*P < 0.05, **P < 0.01, Student’s *t* test.

Having demonstrated that LINC00973-miR-6756-3p-EN2 regulatory axis in HNSCC, we sought to figure out the mechanisms underlying LINC00973 overexpression. Recent reports have provided interesting clues to support SE-driven transcriptional programs underlying lncRNA expression and function across multiple cancers [16, 17, 35]. Inspired by this, we initially interrogated the GeneHancer database and found several SEs within the LINC00973 gene locus, suggesting LINC00973 might be a SE-associated gene [36]. Next, we curated and reanalyzed the publicly available H3K27ac ChIP-seq datasets from HNSCC cell lines for SE identification around LINC00973 locus. As shown in Fig. 5a, b, a proximal SE at chr3:98678906-98697335 was identified across 3 HNSCC cells (hereafter designated LINC00973-SE). This SE structure was consistently identified in multiple cell lines and specimens, but absent in non-tumor epithelial cell lines or normal tissues. Consistently, prominent BRD4/EP300 occupancy and increased chromatin accessibility were observed in LINC00973-SE, consolidating this SE identification (Fig. 5b). Re-analyses of H3K27ac HiChIP and Hi-C datasets from cell lines and primary samples, datasets revealed frequent enhancer-promoter contacts between LINC00973 promoter and LINC00973-SE in HNSCC (Supplementary Fig. S10). Moreover, four enhancer fragments spanning LINC00973-SE were identified and designated as E1-E4. High densities of H3K27ac, BRD4 and EP300 enrichments were detected by ChIP-qPCR across E1-E4 elements but not the control region (E-NC) in both Cal27 and HN6 cells (Fig. 5c). Then we employed BRD4 inhibitor JQ1 and dual p300/CBP and BET bromodomain inhibitor NEO2734 to perturb SE function and found that both chemicals exposure significantly downregulated LINC00973 expression and recoiled the H3K27ac enrichments, BRD4/EP300 binding among E1-E4 (Fig. 5d, e) [20, 37, 38]. In line with this, we developed HNSCC patient-derived xenografts (PDX) model and retrieved samples from animals receiving JQ1 or NEO2734 intraperitoneal injection. As anticipated, both monotherapies substantially inhibited tumor growth (Supplementary Fig. S11a, b) [24]. Subsequent FISH and IHC analyses revealed marked downregulation of LINC00973 and EN2 after SE pharmacological inhibition in vivo (Fig. 5f and Supplementary Fig. S11c). Next, we utilized the improved CRISPR interference (CRISPRi) system (CRISPR-dCas9-KRAB-MeCP2) to clarify the contributions of individual enhancers (E1-4) in activating LINC00973 transcription (Fig. 5g) [28]. Individual repression of E1, 2, 3, 4 by CRISPRi resulted in a remarkable decrease of LINC00973 expression (Fig. 5h). Among them, single E2 repression had the most potent inhibitory effect on LINC00973 expression and we subsequently focused on E2 to explore its repression on cell phenotype. As shown in Fig. 5i–k, E2 repression significantly impaired cell proliferation, migration and invasion in vitro. Collectively, our results confirmed that E1-E4, E2 in particular, were essential to drive LINC00973 expression and HNSCC progression.

Next, we conducted de novo motif analysis to explore the TFs, which might bind to LINC00973-SE and trigger its transcription. As illustrated in Fig. 5l, AP-1/FOSL1 emerged as the predominant TF among these candidates. To experimentally validate this, we employed siRNA-based FOSL1 silencing approach and found remarkable LINC00973 reduction upon FOSL1 depletion in vitro (Fig. 5m and Supplementary Fig. S12). Noticeably, protein co-IP assays confirmed that FOSL1 protein co-bound with BRD4 and RNA Pol II in both Cal27 and HN6 cells, and FOSL1 silencing markedly disrupted these interactions (Fig. 5n, o). To assess the binding dynamics of FOSL1 on LINC00973-SE, we reanalyzed FOSL1 ChIP-seq data from SCC1 cells with/without FOSL1 silencing [39]. As displayed in Fig. 5p, a significant cluster of FOSL1 peaks occurred in the E2 region and FOSL1 silencing dramatically attenuated this enrichment. We further performed ChIP-qPCR and luciferase reporter assays and confirmed that FOSL1 preferentially bound to E2 and subsequently drove LINC00973 transcription (Fig. 5q, r). Complementarily, the RNA abundance of EN2 and miR-6756-3p was notably downregulated following FOSL1-sliencing (Supplementary Fig. S13). Together, these results strongly suggested that FOSL1 bound LINC00973-SE and recruited BRD4 and RNA Pol II to activate LINC00973 transcription in HNSCC (Fig. 5s).

### EN2 activates the NOTCH signaling pathway to promote HNSCC progression

**Fig. 6:**
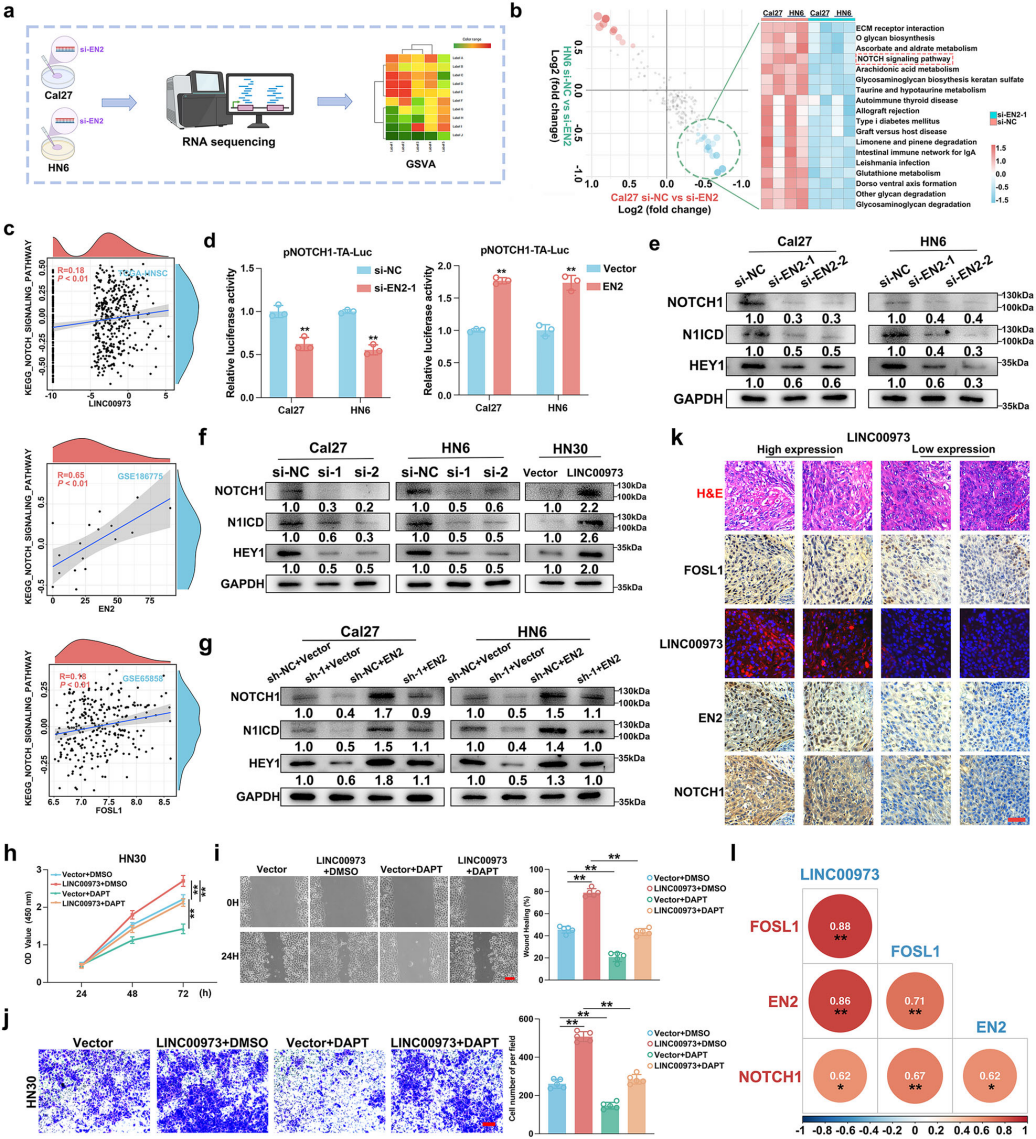
EN2 activates NOTCH signaling pathway to promote HNSCC progression. **a** Schematic diagram outlining the experimental design to identify and characterize downstream signaling pathways modulated by EN2 in HNSCC. **b** Differential analyses of KEGG pathways were performed in Cal27 and HN6 cells with/without EN2 silencing and the results were represented as scatter plot (left panel) and heatmap (right panel). KEGG pathway scores were calculated by GSVA method. **c** Correlation analyses between LINC00973 expression and KEGG_NOTCH_SIGNALING_PATHWAY scores (GSVA method) in TCGA-HNSC, GSE186775 and GSE65858 datasets. **d** The transcription activity of NOTCH1, the core member of NOTCH pathway, was measured by NOTCH1 luciferase reporter assays in Cal27 and HN6 with EN2 manipulations. **e** The protein abundance of NOTCH1, N1ICD and HEY1 were determined by western blot in Cal27 and HN6 cells with EN2 knockdown. **f** The endogenous NOTCH1, N1ICD and HEY1 protein expression was detected in Cal27 and HN6 cells followed by LINC00973 siRNAs or HN30 cells with LINC00973 overexpression, respectively. **g** NOTCH1, N1ICD and HEY1 were determined by western blot in Cal27 and HN6 cells with/without LINC00973 knockdown coupled with/withoutEN2 overexpression. Cell proliferation, migration and invasion were significantly increased following LINC00973 overexpression but reduced by DAPT treatment as gauged by CCK-8 (**h**), wound healing (**i**) and Transwell invasion assays (**j**).Scale bar: 50 μm. **k** Representative H&E, IHC staining of FOSL1, EN2, and NOTCH1 as well as FISH staining of LINC00973, in serial sections of the same HNSCC clinical samples were shown. Scale bar: 50 μm. **l** The correlation among FOSL1, EN2 and NOTCH1 protein expression (IHC quantifications data) and LINC00973 RNA abundance (FISH signal intensity) were analyzed by using Spearman’s correlation. Data were presented as mean ± SD, **P < 0.01, Student’s *t* test.

Finally, to briefly explore the mechanisms by which EN2 promoted HNSCC progression, we performed RNA-seq in Cal27 and HN6 cells following EN2 silencing. Hundreds of genes with altered expression were identified and these DEGs were overrepresented in multiple oncogenic signaling (Fig. 6a). Notably, 18 pathways were consistently downregulated in both cell lines upon EN2 silencing, including NOTCH signaling pathway (Fig. 6b). Additionally, significant positive associations were found between NOTCH pathway scores and LINC00973/EN2/FOSL1 expression in bulk transcriptomics datasets (Fig. 6c). Next, we measured NOTCH1, N1ICD and HEY1 expression and performed a NOTCH1 luciferase reporter assay as a proxy readout for NOTCH pathway activity and found that EN2 positively modulated these markers protein levels and NOTCH1 transcriptional activity in vitro (Fig. 6d, e). Moreover, LINC00973 positively modulated NOTCH1, N1ICD and HEY1 levels in vitro and EN2 overexpression effectively abrogated LINC00973 knockdown-mediated inhibitory effects (Fig. 6f, g). Next, we utilized a NOTCH pathway inhibitor DAPT and found that DAPT effectively abrogated LINC00973 overexpression-induced effects on cell proliferation, migration, and invasion in vitro (Fig. 6h–j). To extend these findings to clinical context, we performed IHC staining for FOSL1, EN2 and NOTCH1 and LINC00973 FISH assays in HNSCC samples. Of note, significant positive correlations among these markers were detected (Fig. 6k, l). Collectively, these findings delineate that EN2 mediate the tumorigenic roles of LINC00973 to facilitate HNSCC progression, probably via NOTCH pathway activation.

## Discussion

**Fig. 7:**
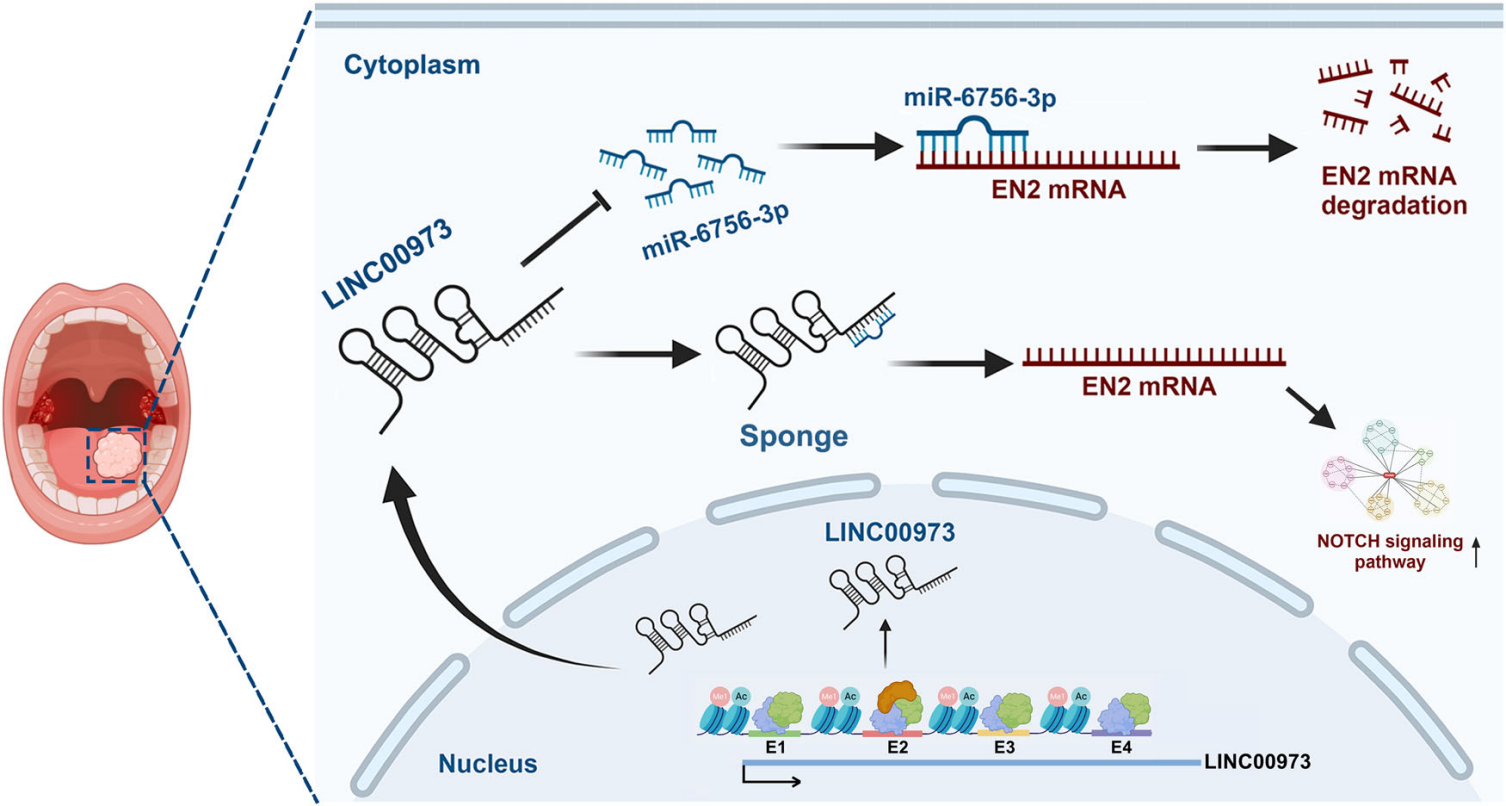
Schematic illustration of SE-driven LINC00973-miR-6756-3p-EN2-NOTCH regulatory axis to facilitate malignant behavior of HNSCC. The proximal SE in LINC00973 locus *cis*-activates LINC00973 transcription via recruiting AP-1/FOSL1, BRD4 and EP300; the cytoplasmically localized LINC00973 sponges miR-6756-3p to increase the mRNA half-life of EN2; EN2 activates downstream NOTCH signaling to modulate the aggressiveness of HNSCC.

The past decade has witnessed substantial efforts and remarkable progresses in characterizing the lncRNA landscape in various cancers and establishing putative pro-tumorigenic or tumor-suppressor roles of selected lncRNAs during cancer initiation and progression. LncRNA usually serves as molecular sponge to miRNA and in turn relieves miRNA-cognate targets to drive cancer overgrowth, metastatic spread and therapeutic resistance across multiple cancers [5, 6]. Here, we identify LINC00973 as a novel oncogenic lncRNA with prognostic significance and establish the AP-1/FOSL1-activated SE-driven LINC00973-miR-6756-3p-EN2 regulatory axis to facilitate HNSCC progression by integrative bioinformatics data analyses, epigenomics-transcriptomics profiling, genetic manipulations, multiple preclinical models and clinical patient cohorts (Fig. 7). These findings shed light on intricate crosstalk between SE, lncRNA, as well as miRNA to cooperatively drive HNSCC progression.

### The expression patterns and clinical significance of LINC00973-miR-6756-3p-EN2 axis in HNSCC

Several lines of evidence have shown that LINC00973 is abnormally upregulated in a few human cancers and associated with tumor aggressiveness, tumor immune escape and unfavorable prognosis [9, 11, 40]. Its overexpression significantly correlated with tumor size, lymphatic metastasis, tumor stages, and poor outcomes in patients with clear-cell renal cell carcinoma [9]. Here, we found that LINC00973 was remarkably elevated and associated with aggressive clinicopathological features and poor prognosis in HNSCC samples, which was consistently supported by bioinformatics analyses of multiple datasets and in-house samples. Importantly, LINC00973 expression status was identified as an independent risk factor to predict patient survival, thus underscoring LINC00973 with prognostic relevance in HNSCC. On the other hand, previous reports have indicated that EN2 has been identified as a putative oncogene and prognostic biomarker under diverse tumor contexts, including colorectal cancer [33], ovarian cancer [41], prostate cancer [42] and breast cancer [31]. These results collectively suggested EN2 as a common prognostic predictor irrespective of cancer types. Consistent with this, our results reconfirmed EN2/miR-6756-3p as promising prognostic biomarkers for HNSCC. Of great interest and importance, EN2 and miR-6756-3p are readily detected in urine and plasma in patients with prostate or ovarian cancer, respectively [41, 42]. Moreover, salivary miRNA miR-423-5p has been identified as a promising diagnostic and prognostic biomarker in oral squamous cell carcinoma [43]. We speculated that salivary or circulating EN2/miR-6756-3p might be promising non-invasive biomarkers with translational potential for HNSCC, which deserves further exploration.

### LINC00973 functions as a ceRNA to sponge miR-6756-3p and stabilize EN2 mRNA

Previous reports have uncovered the pro-tumorigenic roles of LINC00973 through promoting cell proliferation and immune escape in several malignancies [9, 11, 44]. In line with this, our scRNA-seq dataset reanalysis and FISH assays revealed that LINC00973 was highly expressed in malignant epithelial cells in HNSCC, suggesting its critical involvement in modulating aggressive epithelial phenotypes. Moreover, loss-of-function assays and preclinical orthotopic models provided ample evidence that LINC00973 enhanced cell proliferation, migration and invasion both in vivo and vitro. Thus, these results establish LINC00973 as a putative pro-tumorigenic lncRNA to fuel HNSCC progression.

Prior studies offered clues to support that LINC00973 might serve as a ceRNA to modulate oncogenic gene expression through sponging specific miRNAs in cancer [9, 11]. Consistent with this, our results indicated that LINC00973 competitively adsorbed miR-6756-3p to protect EN2 mRNA from endogenous miRNA-mediated degradation in HNSCC as reflected by the following evidences. Firstly, the predominant cytoplasmic localization of LINC00973 in HNSCC malignant epithelial cells largely determined its roles at a post-transcriptional or translational level. Next, computational prediction followed by RIP assays strongly supported that LINC00973 bound with AGO2-miR-6756-3p complex in HNSCC cells. Lastly, our miRNA mimics/inhibitors treatments, rescue experiments and luciferase reporter assays established EN2 as a direct post-transcriptional target of miR-6756-3p and confirmed miR-6756-3p as the key mediator of LINC00973 to exert its oncogenic effects in HNSCC. Collectively, our findings extend the mechanistic understanding of LINC00973-mediated ceRNA network during tumor progression. However, we cannot rule out the possibility that other miRNAs beyond miR-6756-3p might also contribute to LINC00973 pro-tumorigenic effects in HNSCC, which warrants further investigations.

### AP1/FOSL1 *trans*-activates LINC00973 transcription via binding its SE

To date, the upstream regulatory mechanism responsible for LINC00973 transcription in cancer remain underexplored. The only available report came from Shao et al., who reported that β-catenin/TCF/LEF complex bound to the TBE2 region on LINC00973 promoter to activate its transcription in non-small cell lung carcinoma [11]. Interestingly, recent reports have pointed to SE as a novel regulatory element to drive multiple lncRNA overexpression through aberrant TFs recruitment and hijacking transcriptional machinery among several human cancers [16]. In keeping with this, we identified a previously unreported SE at the LINC00973 locus and revealed that LINC00973-SE activated LINC00973 transcription potentially via enhancer-promoter interaction in HNSCC, as evidenced by H3K27ac ChIP-seq, Hi-ChIP/Hi-C data re-analyses, gene expression upon SE-targeting chemicals, as well as dCas9-KRAB CRISPRi. However, given the unique nature of lncRNA and the genomic location of this LINC00973-SE at the gene body, much work is needed to further pinpoint the detailed mechanisms underlying SE-driven LINC00973 transcription in HNSCC [45]. Furthermore, we found prominent AP-1/FOSL1 motif enrichment within LINC00973-SE and experimentally validated FOSL1 preferentially bound to LINC00973-SE (especial E2 in this SE) and recruited BRD4, EP300 and Pol II to activate its transcription. This is well consistent with FOSL1 as a key oncogenic TF to promote aggressive behaviors in HNSCC, primarily via preferentially binding with SE to facilitate oncogene transcription [46]. Previous studies have revealed that FOSL1 and BRD4 tend to be assembled in SE via phase-separated condensates to drive SE-associated gene expression [47, 48]. Our co-IP and ChIP-qPCR results confirmed interactions between BRD4 and FOSL1 proteins and their marked enrichments at LINC00973-SE, providing support for their cooperative function to promote LINC00973 transcription. We reasoned that FOSL1 and BRD4 might synergistically activate LINC00973 transcription via phase separation, which warranted further experimental validation. Together, we conclude that FOSL1 activates LINC00973 transcription via binding its SE region, thus suggesting another layer of regulation involved in LINC00973 upregulation in cancer. Moreover, results from these preclinical models suggest that targeting SE by chemical compounds to disrupt aberrant transcription of these SE-driven oncogenic genes or non-coding RNAs might be a novel, viable therapeutic strategy against HNSCC.

### EN2 activates NOTCH signaling in HNSCC

Previous reports have delineated EN2 as an oncogenic TF with both promoter and enhancer binding potentials to drive tumorigenesis [33, 49]. For example, EN2 binds with the CCL2 promoter and activates its transcription, thereby facilitating cell proliferation and migration in colorectal cancer [33]. Our GSEA analyses based on DEGs in TCGA-HNSC samples and GSVA analyses of RNA-seq following EN2 silencing suggested that the NOTCH pathway was critically involved in LINC00973 and EN2 to facilitate HNSCC progression. Indeed, the NOTCH pathway has been well-established to modulate cell fate decisions, differentiation, and stemness during HNSCC development [50–52]. In addition, positive correlations between EN2 and NOTCH1 in primary clinical samples offer further support for NOTCH signaling involved in EN2-driven HNSCC progression. However, we are not able to determine the direct binding of EN2 at the cis-elements of NOTCH pathway members due to the paucity of ChIP-grade EN2 antibody. Much work is needed to pinpoint the precise mechanisms underlying EN2 regulatory effects on the NOTCH pathway in HNSCC.

In conclusion, our results unveil a previously unrecognized SE-driven LINC00973-miR-6756-3p-EN2 regulatory axis which drives aggressive phenotypes and portends unfavorable outcomes in HNSCC. These findings highlight LINC00973-miR-6756-3p-EN2 as potential prognostic biomarkers and therapeutic targets with translational values in HNSCC.

## Supplementary information

Supplementary Figures and Tables-RE

Uncropped Gels and Blots image

Supplementary_Figure 1

Supplementary_Figure 2

Supplementary_Figure 3

Supplementary_Figure 4

Supplementary_Figure 5

Supplementary_Figure 6

Supplementary_Figure 7

Supplementary_Figure 8

Supplementary_Figure 9

Supplementary_Figure 10

Supplementary_Figure 11

Supplementary_Figure 12

Supplementary_Figure 13

## Data Availability

The transcriptomic data of HNSCC patients, single-cell transcriptomics, ChIP-seq and ATAC-seq were sourced from publicly available repositories (TCGA, GEO, and GSA for Human). Additional bulk RNA-seq data generated in this study have been deposited in the GSA for Human database under accession number HRA011140. The materials used are available from the corresponding authors upon reasonable request.
