# Supplementary material for: A novel super-enhancer-driven lncRNA LINC00973 governs head and neck squamous cell carcinoma progression through EN2
Source: Cell Death Dis. 2025 Dec 19;17(1):111. doi: 10.1038/s41419-025-08380-8 (PMC12848070; doi:10.1038/s41419-025-08380-8)
Supplement: Supplementary file 1 — Supplementary Figures and Tables-RE [file 41419_2025_8380_MOESM1_ESM.doc]

**
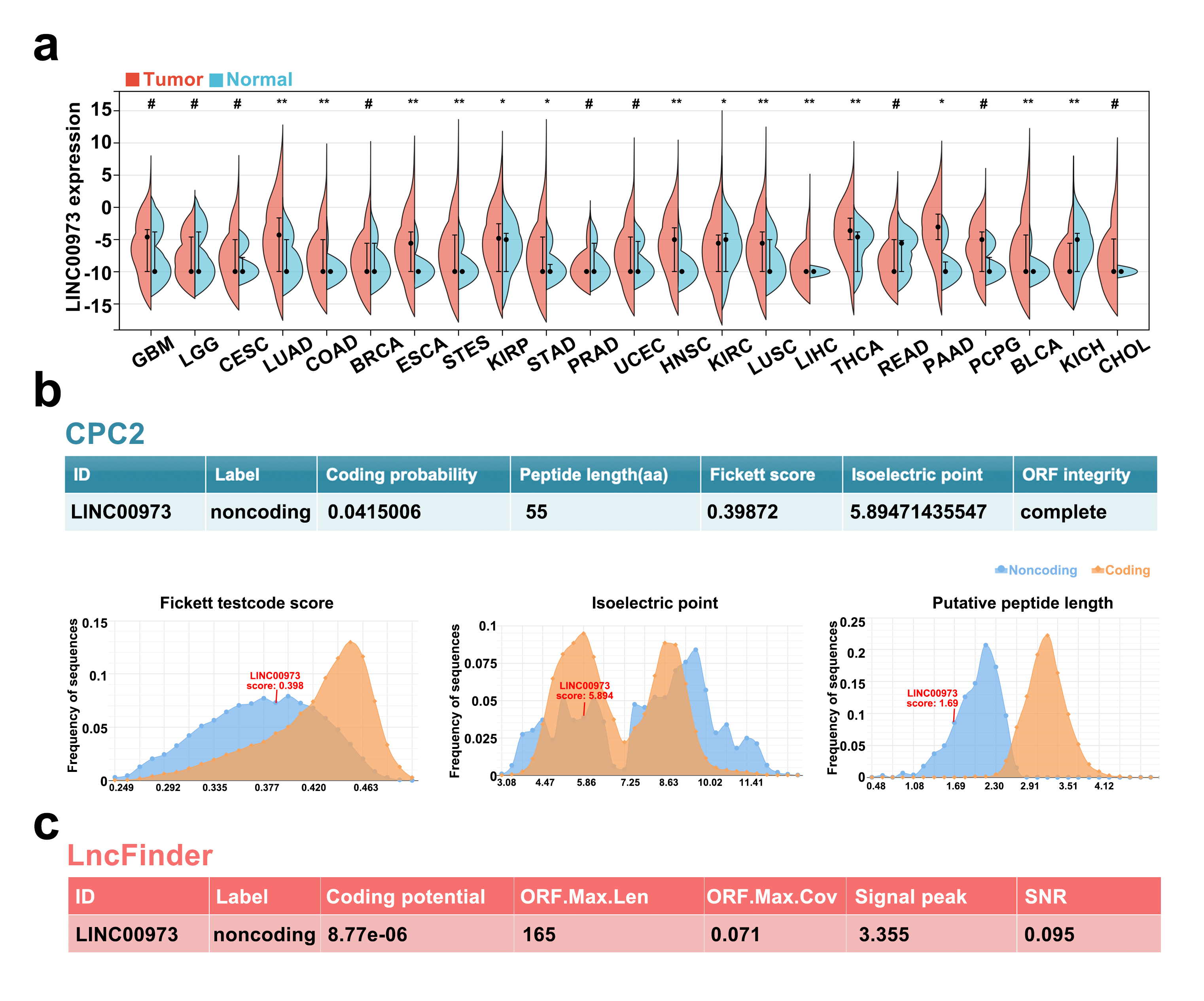
**

**Supplementary Figure S1. LINC00973 is a putative oncogenic lncRNA with limited coding potential across diverse cancer contexts.**

**a.** The expression levels of LINC00973 across various human cancers and corresponding normal tissue were extracted from the TCGA-PanCancer dataset and compared. Wilcoxon rank-sum test.

**b, c** The coding potential of LINC00973 was confirmed by CPC2 (**b**) and LncFinder (**c**).

^#^*P* ≥ 0.05, **P* < 0.05, ***P* < 0.01.

**
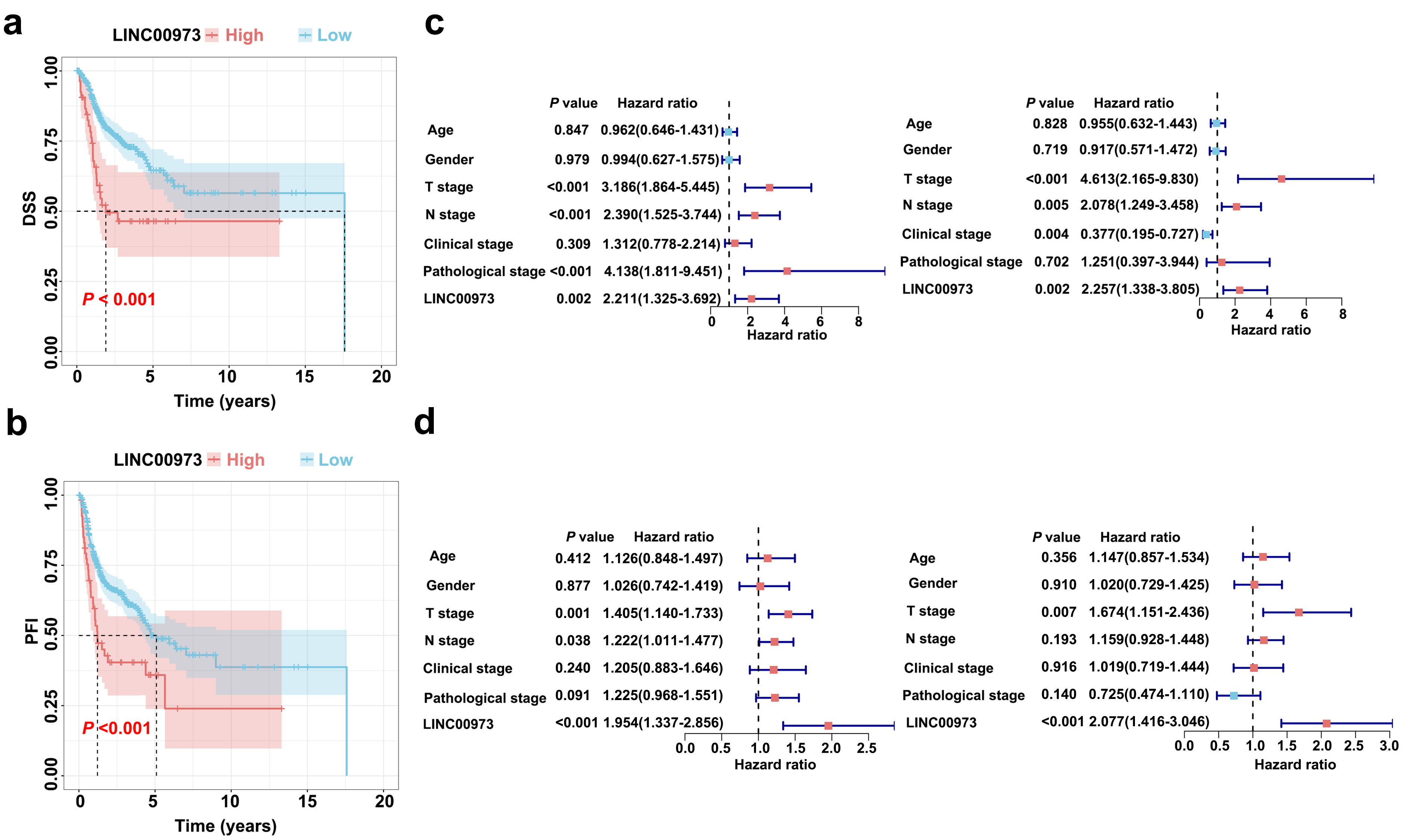
**

**Supplementary Figure S2. The prognostication of LINC00973 in TCGA-HNSC dataset**

**a:** Kaplan-Meier analysis of DSS based on optimal cut-off point of LINC00973 expression in the TCGA-HNSC cohort. Log-rank test.

**b:** Kaplan-Meier analysis of PFI based on optimal cut-off point of LINC00973 expression in the TCGA-HNSC cohort. Log-rank test.

**c:** Forest plot displayed univariate and multivariate Cox regression analyses of multiple clinicopathological factors as well as LINC00973 expression based on DSS in the TCGA-HNSC dataset.

**d:** Forest plot displayed univariate and multivariate Cox regression analyses of multiple clinicopathological factors as well as LINC00973 expression based on PFI in the TCGA-HNSC dataset.

**
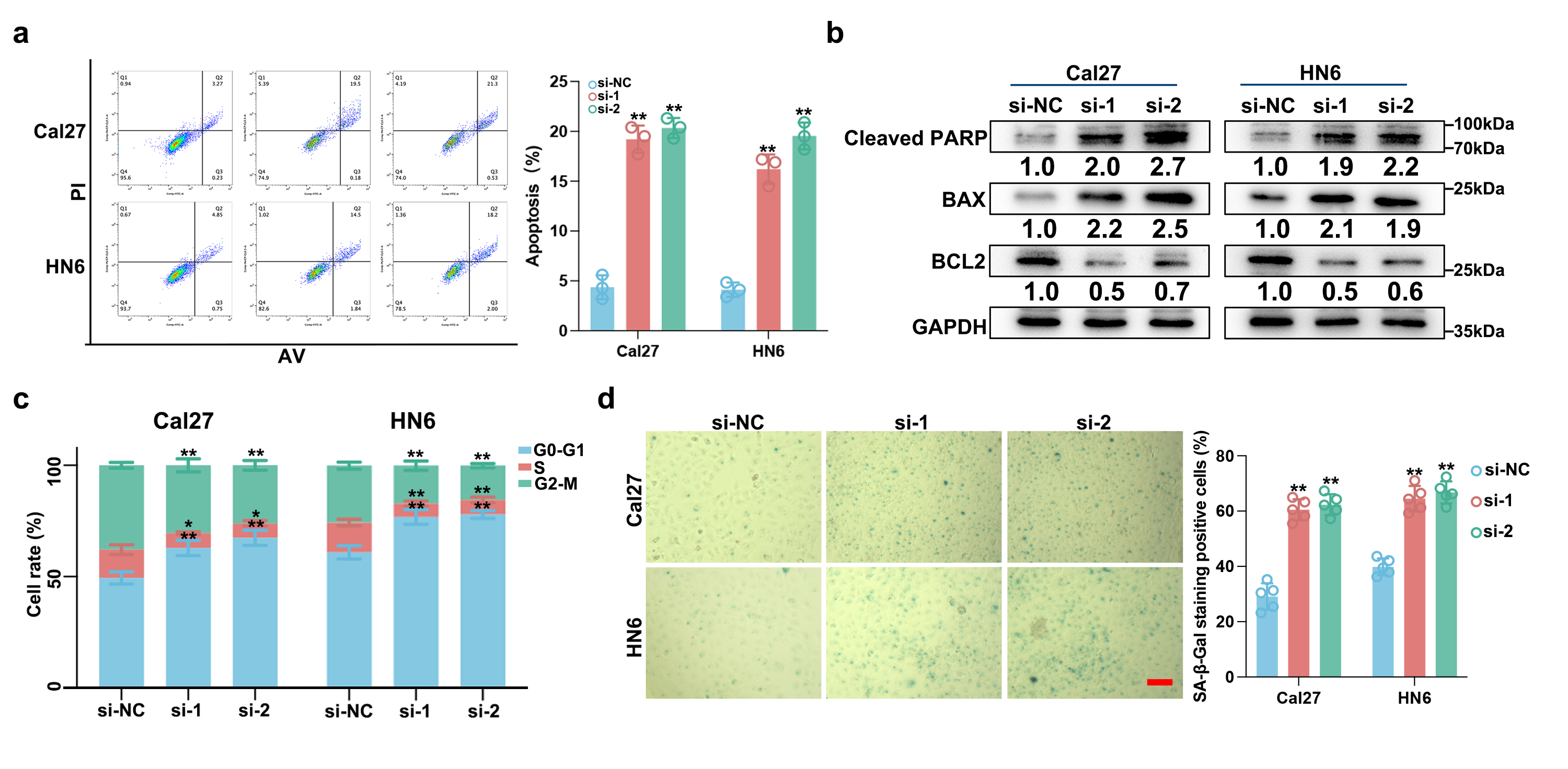
**

**Supplementary Figure S3. LINC00973 knockdown induced HNSCC apoptosis, cell cycle arrest and senescence.**

**a, b:** Increased percentages of apoptotic cells were observed following LINC00973 knockdown as assayed by Annexin V-PI staining and expression changes of relevant markers.

**c:** Flow cytometry analyses of cell cycle distribution after knockdown of LINC00973.

**d:** Senescence cells upon LINC00973 knockdown were determined by SA-β-gal staining and quantified. Scale bar: 50 μm.

^#^*P* ≥ 0.05, **P* < 0.05, ***P* < 0.01.

**
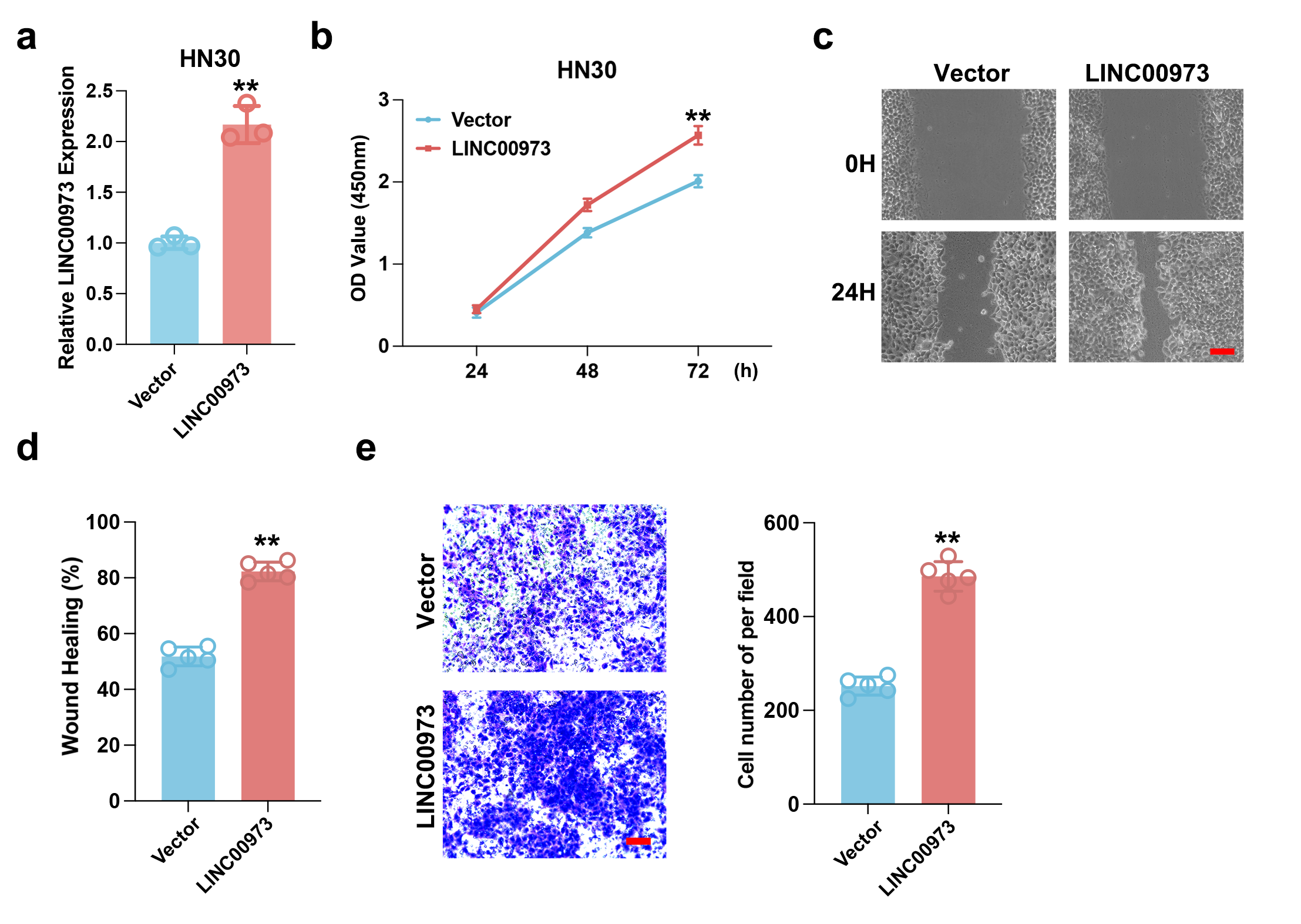
**

**Supplementary Figure S4. LINC00973 overexpression induced HNSCC cells malignant phenotypes.**

**a:** The mRNA abundance of LINC00973 was measured in HN30 cells post-transfected with LINC00973 cDNA plasmid.

**b-e:** Cell proliferation, migration and invasion were significantly induced following LINC00973 overexpression by CCK-8 (b), wound healing (c, d) and Transwell invasion assays (e). Scale bar: 50μm.

^#^*P* ≥ 0.05, **P* < 0.05, ***P* < 0.01.

**
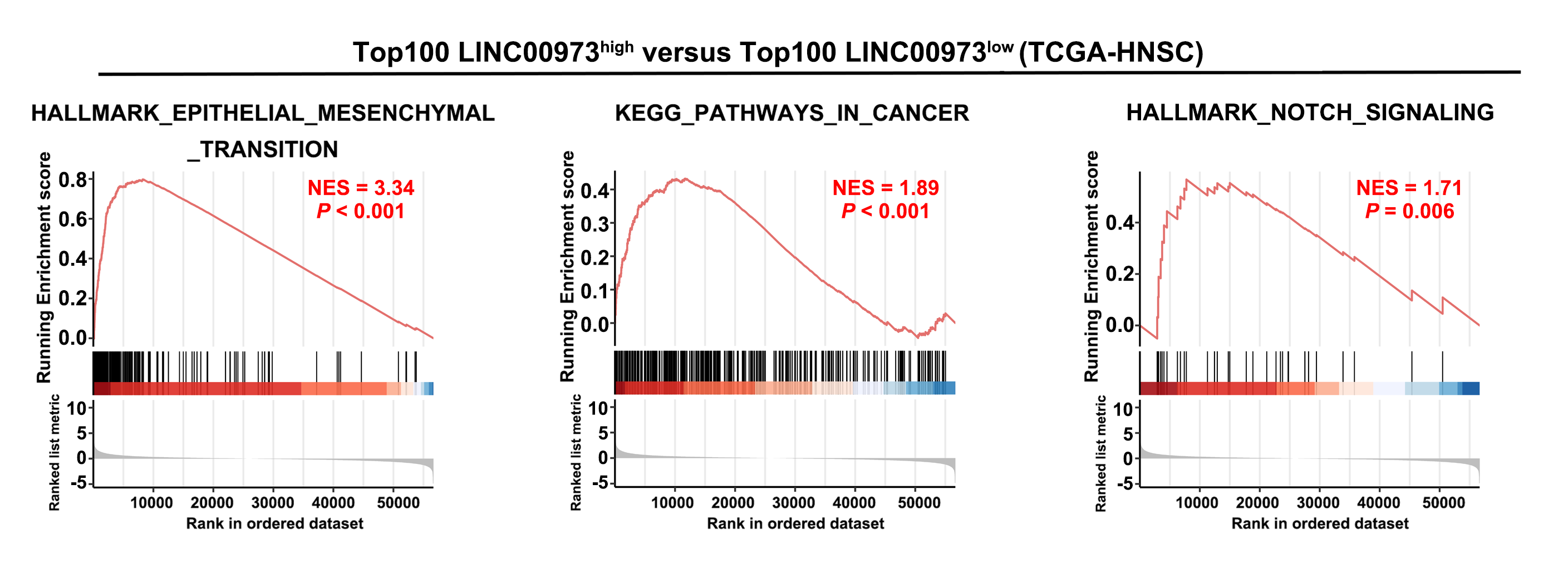
**

**Supplementary Figure S5. DEG and GSEA analyses in patients of TCGA-HNSC dataset with higher expression of LINC00973 (n = 100) compared to those with lower expression of LINC00973 (n = 100).**

**
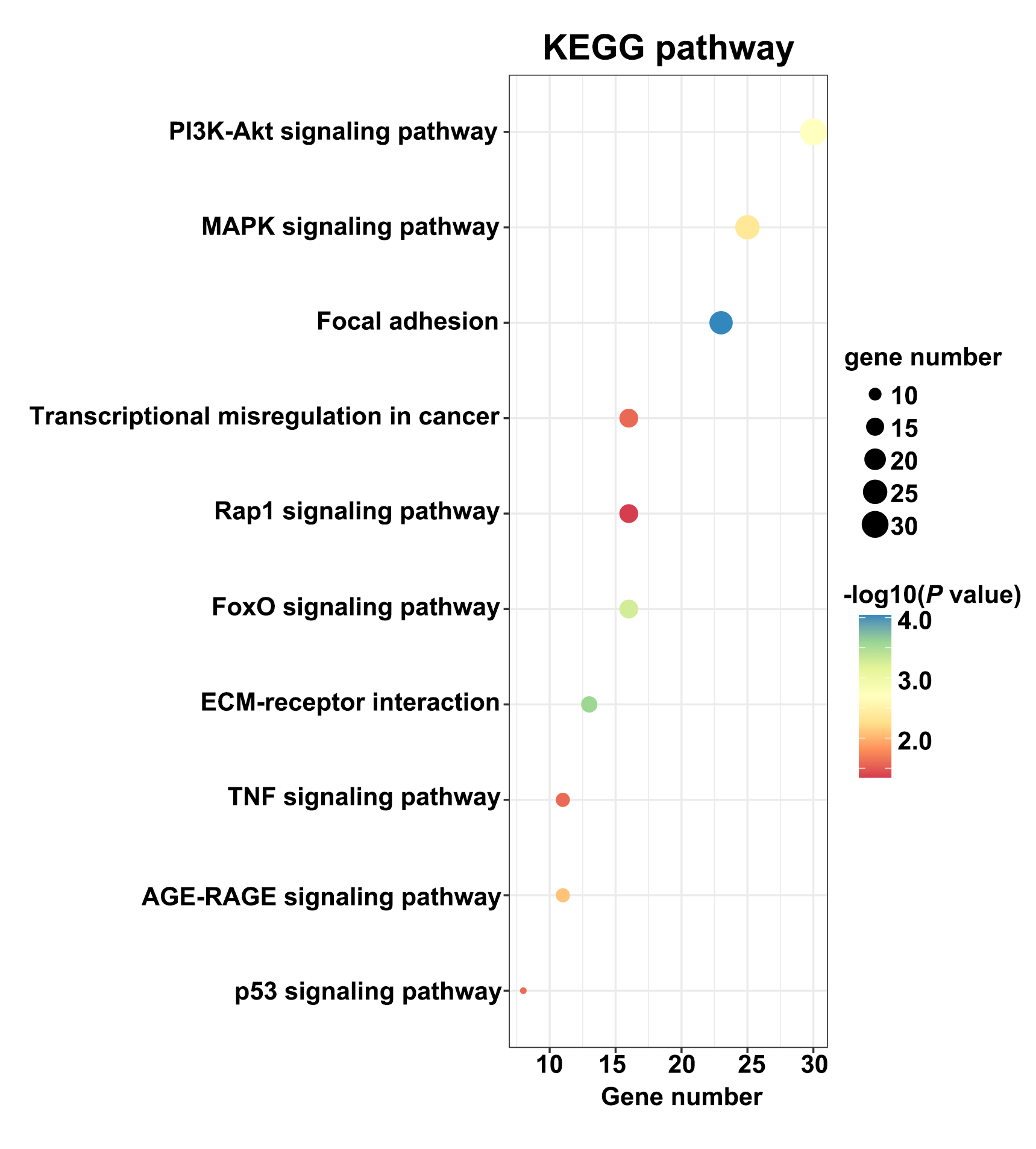
**

**Supplementary Figure S6. KEGG analysis of DEGs following LINC00973 knockdown.**

**
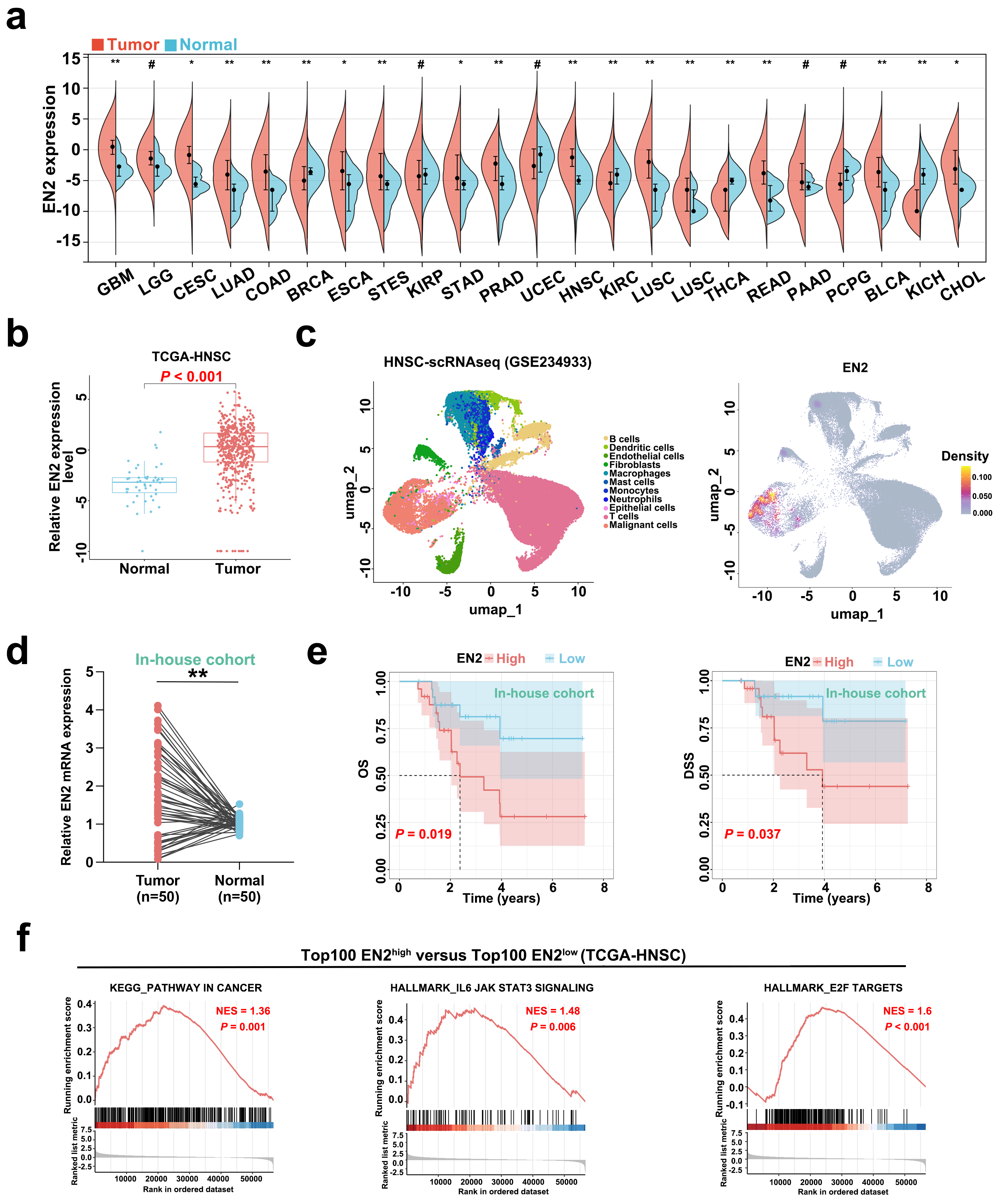
**

**Supplementary Figure S7. EN2 was highly expressed and associated with patients’ prognosis in HNSCC.**

a: The expression pattern of EN2 in TCGA-PanCancer dataset was analyzed and compared.

across various human cancers and corresponding normal tissue were extracted from the TCGA-PanCancer dataset and compared. Wilcoxon rank-sum test.

**b:** EN2 was overexpressed in HNSCC samples in TCGA-HNSC dataset.

**c:** UMAP plots displayed the 11 identified main cell types (left panel) and the distribution of EN2 (right panel) shown.

**d:** qRT-PCR was utilized to measure the expression of EN2 in 50 HNSCC clinical samples. Paired wilcoxon rank-sum test.

**e:** Kaplan-Meier analyses of OS and DSS based on median expression of LINC00973 in the 50 HNSCC clinical samples. Log-rank test.

**f:** DEG and GSEA from patients in TCGA-HNSC with higher expression of LINC00973 (n = 100) compared to those with lower expression of LINC00973 (n = 100).

^#^*P* ≥ 0.05, **P* < 0.05, ***P* < 0.01.


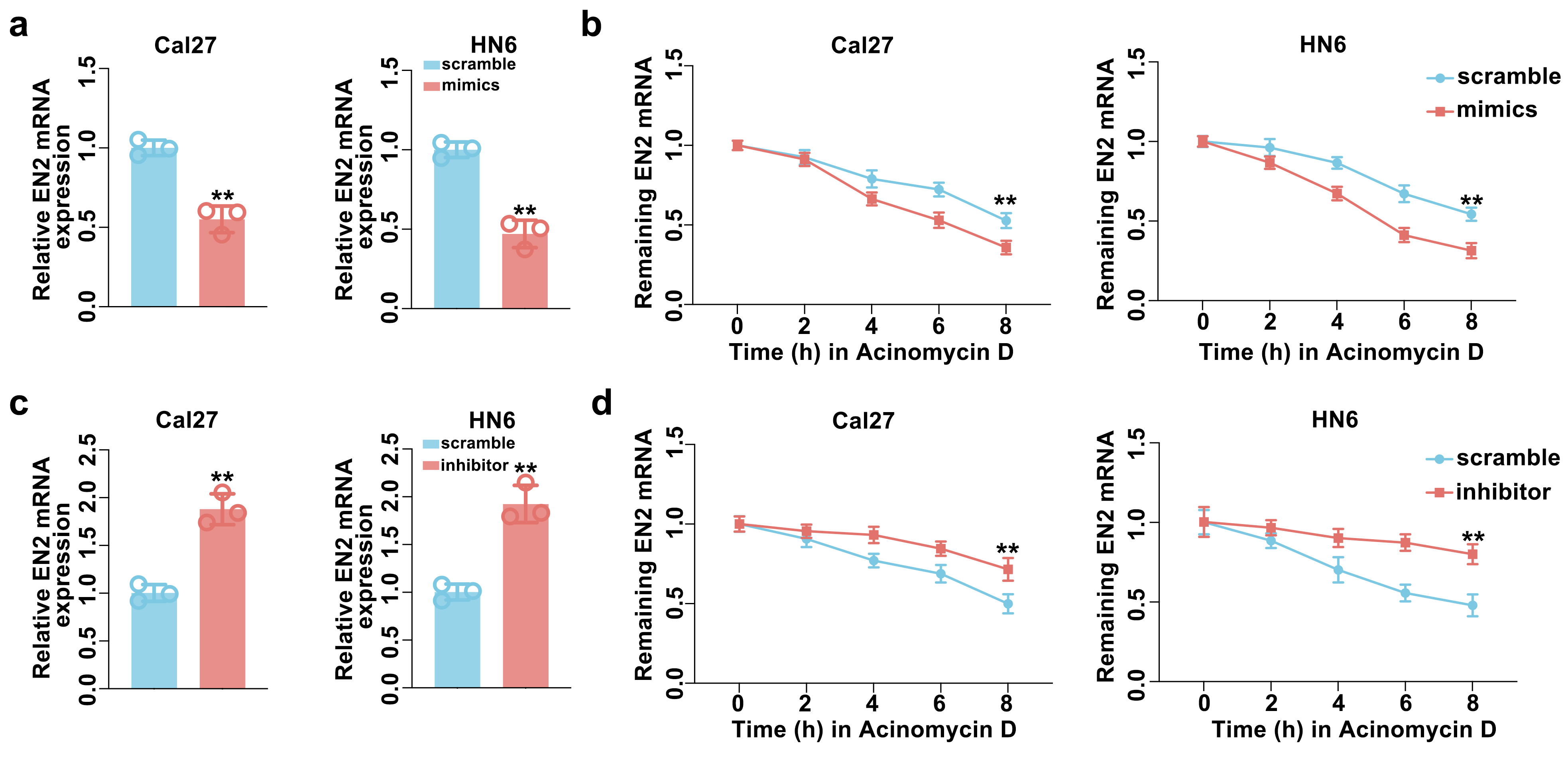


**Supplementary Figure S8. miR-6756-3p induced EN2 mRNA decay.**

**a, b:** EN2 expression (**a**) and mRNA half-time (**b**) was measured by qRT-PCR in Cal27 and HN6 cells transfected with miR-6756-3p mimics.

**c, d:** EN2 expression (**c**) and mRNA half-time (**d**) was determined by qRT-PCR in Cal27 and HN6 cells with miR-6756-3p inhibitor exposure.

Data were presented as mean±SD, ***P* < 0.01, Student’s *t* test.

**
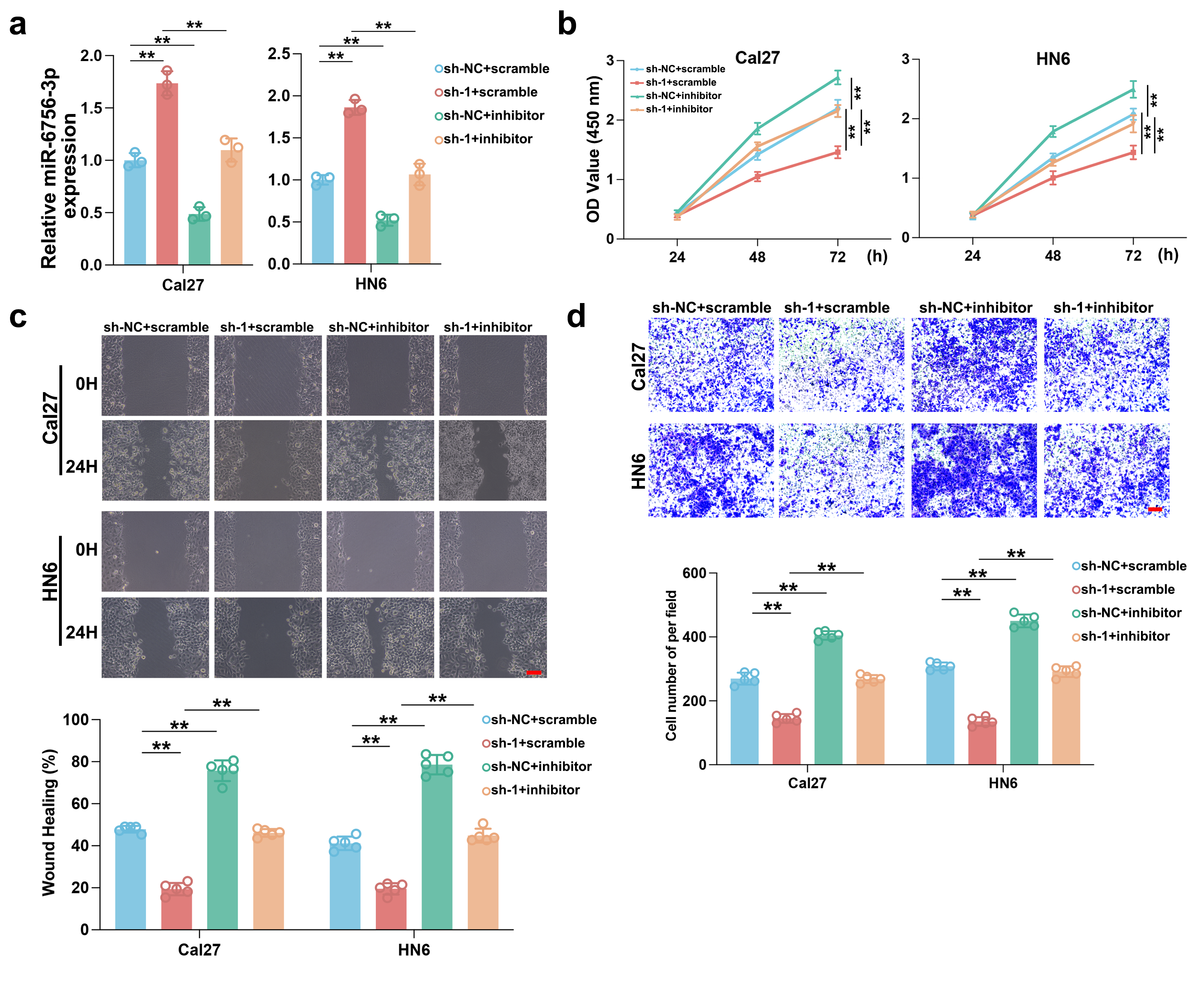
**

**Supplementary Figure S9. LINC00973 regulate miR-6756-3p expression to facilitate HNSCC tumorigenesis.**

**a:** The RNA abundance of miR-6756-3p were measured in Cal27 and HN6 cells under four treatment conditions.

**b-d:** Cell proliferation, migration and invasion were significantly reduced following LINC00973 knockdown but restored by ectopic miR-6756-3p inhibition as gauged by CCK-8 (b), wound healing (c) and Transwell invasion assays (d). Scale bar: 50μm.

^#^*P* ≥ 0.05, **P* < 0.05, ***P* < 0.01.

**
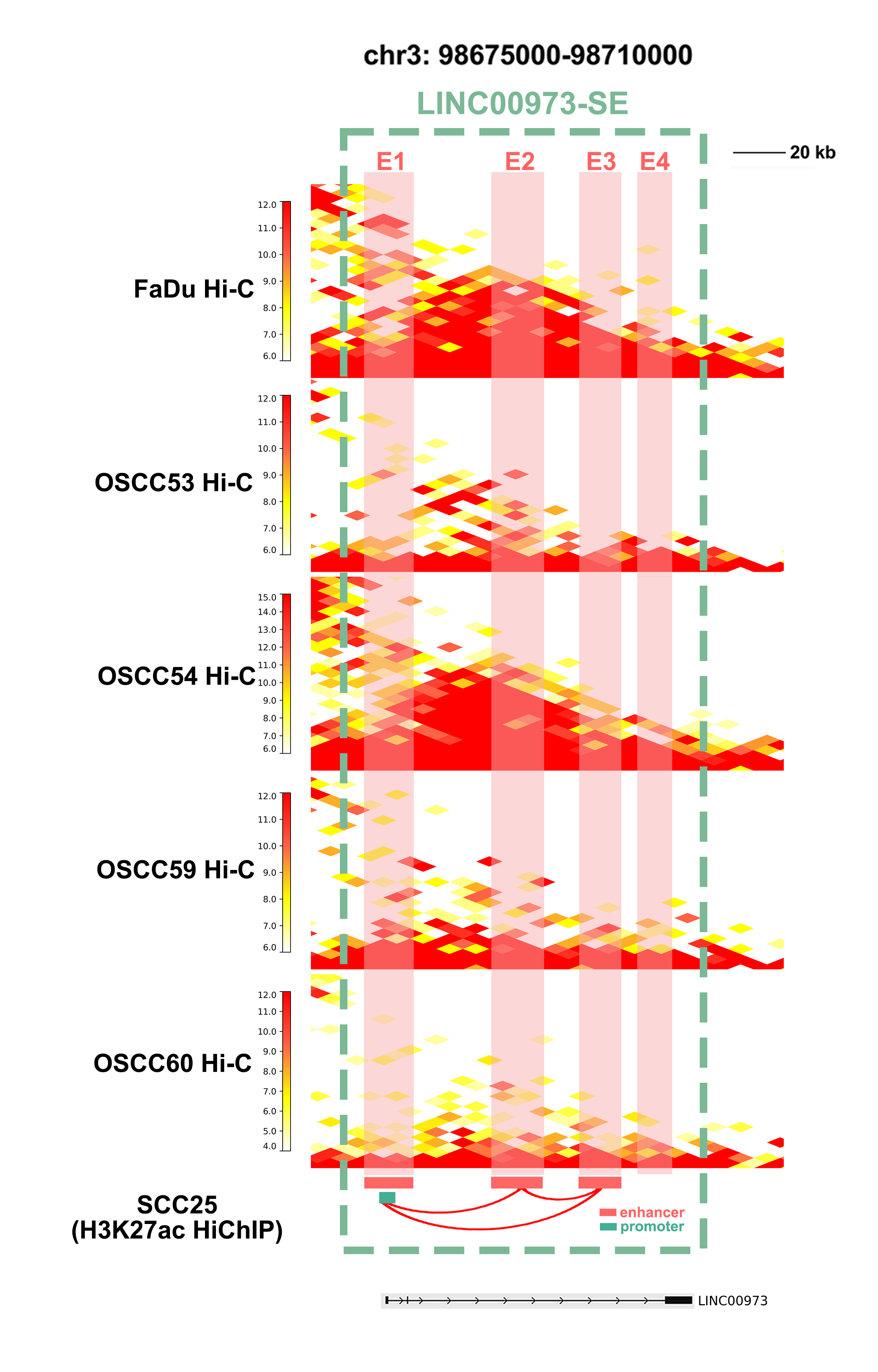
**

**Supplementary Figure S10.** Hi-C contact map sourced from FaDu cells and four primary OSCC samples showed the high 3D gnomic DNA contract frequency within LINC00973-SE/promoter regions. The lowest panel showed H3K27ac HiChIP loop at the same region using SCC25 H3K27ac HiChIP data. LINC00973 promoter (P) was defined from chr3: 98,680,0325 to chr3: 98,681,166 (based on H3K27ac peaks and transcription start site (TSS)). It was 841bp in length, locating from -665bp TSS upstream to downstream +176bp, which was partially overlapped with E1.

**
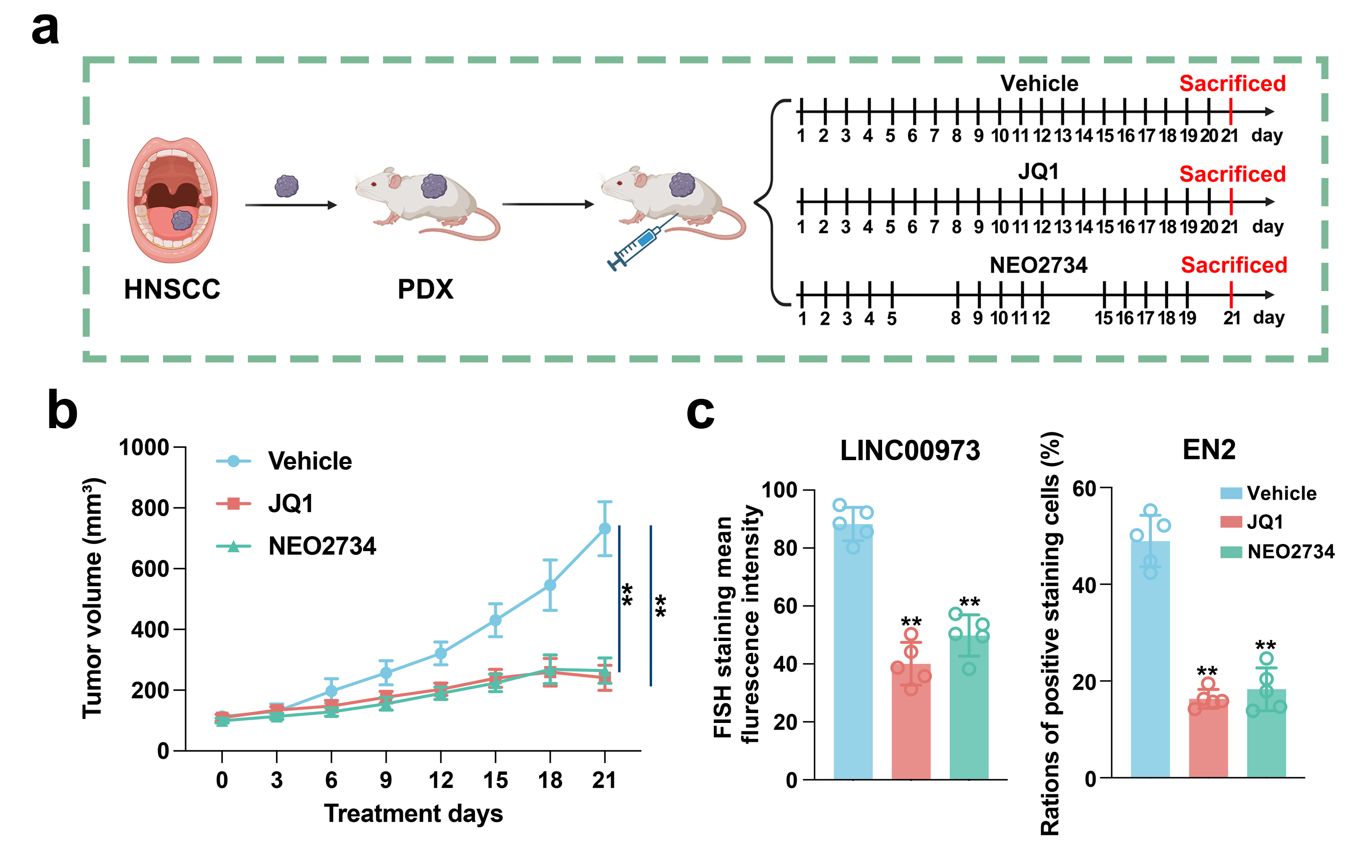
**

**Supplementary Figure S11. JQ1 or NEO2734 treatment impaired the PDX masses growth as well as reduced LINC00973 and EN2 expression *in vivo*.**

**a:** Schematic description of experimental procedures for HNSCC PDX development and the administration of scheduled drugs were illustrated.

**b:** The tumor volume of PDX tumors were monitored after JQ1/NEO2734 treatments.

**c:** Quantification data of representative FISH staining of LINC00973 and IHC staining of EN2 were shown in PDX masses.

Data were presented as mean±SD, ***P* < 0.01, Student’s *t* or one-way ANOVA test.

**
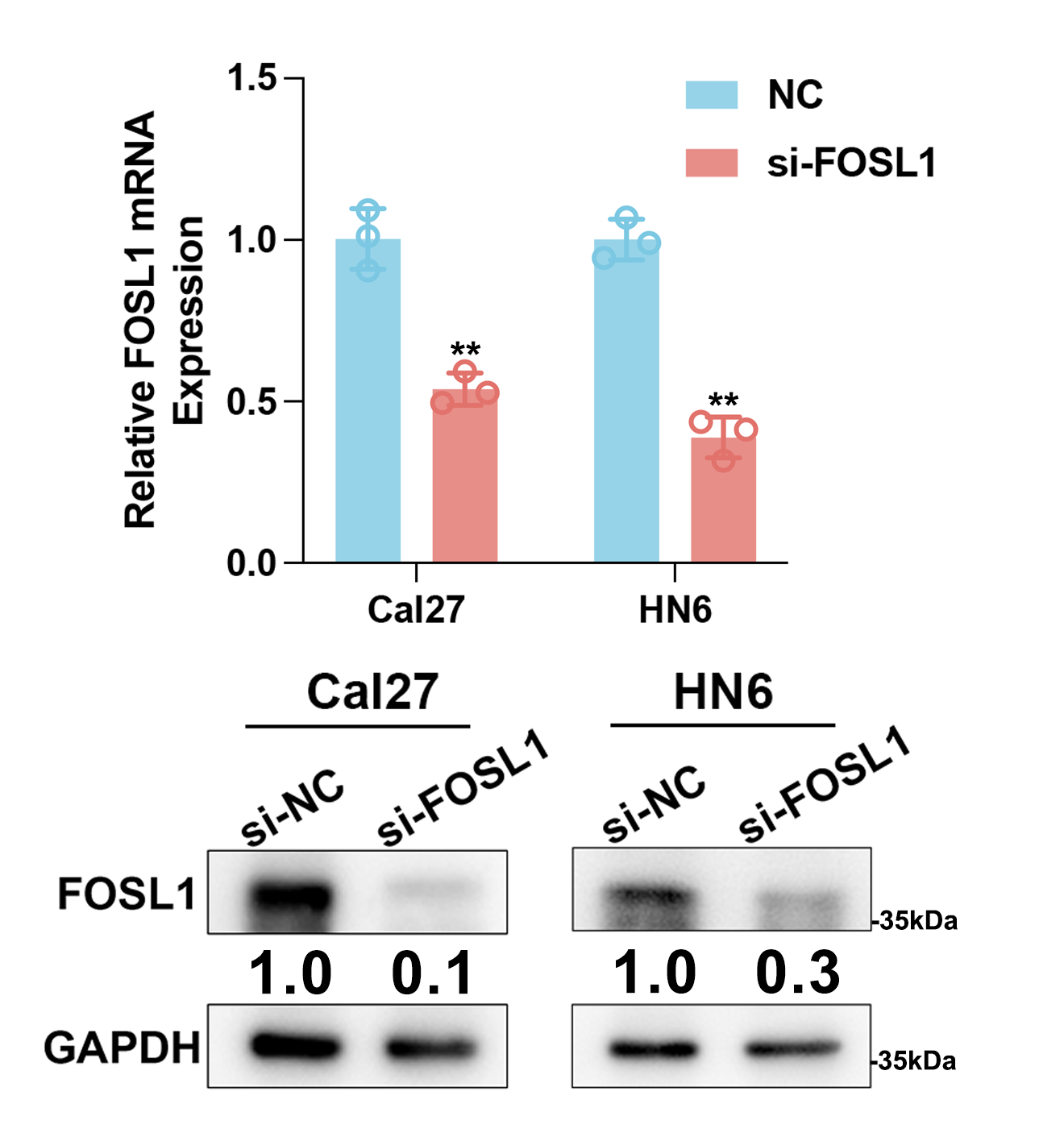
**

**Supplementary Figure S12. The mRNA and protein abundance were remarkably reduced after FOSL1-targeting siRNAs transfection in HNSCC.**

Data were presented as mean±SD, ***P* < 0.01, Student’s *t* test.

**
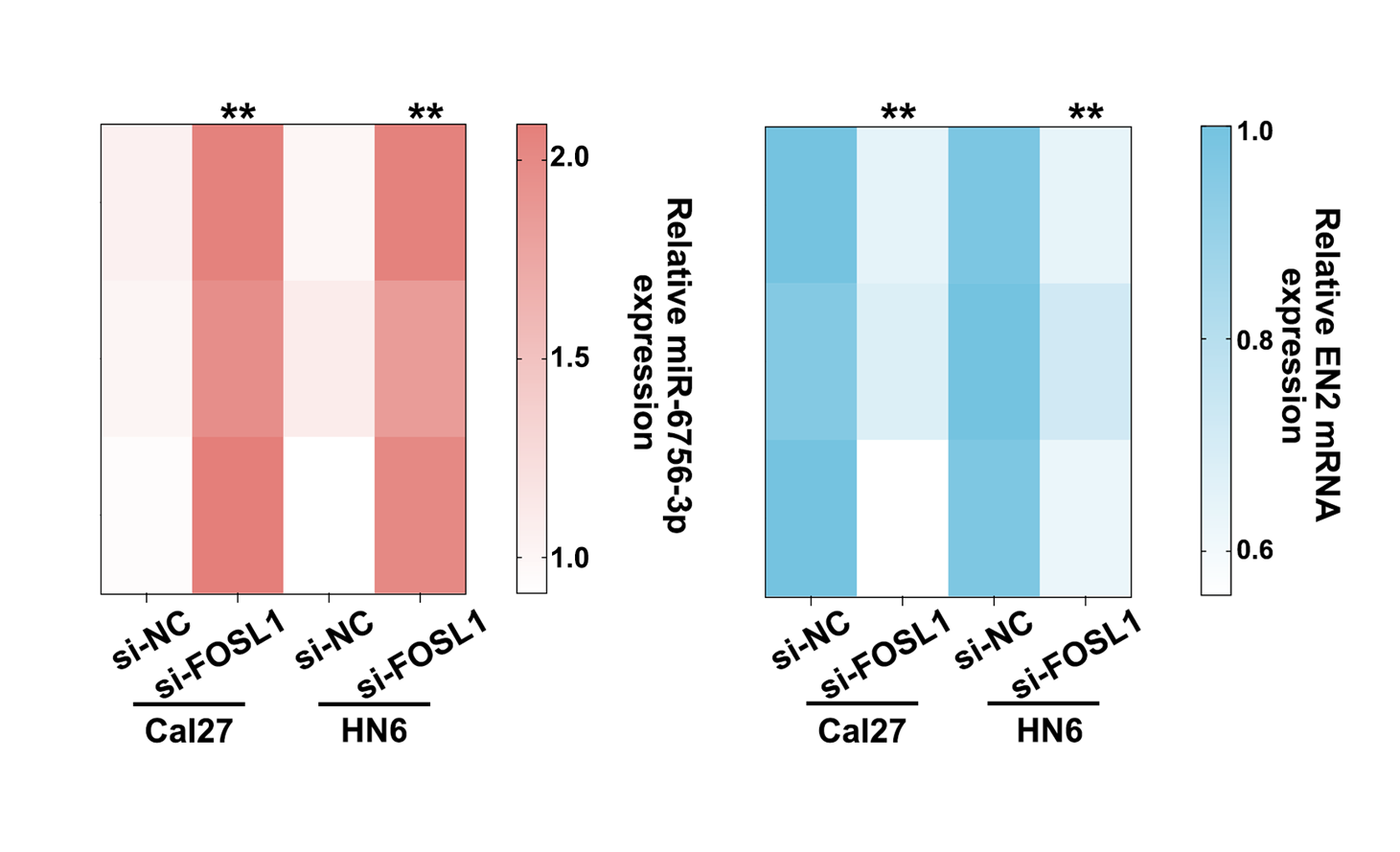
**

**Supplementary Figure S13. EN2 mRNA levels were assessed in Cal27 and HN6 cells with LINC00973/ miR-6756-3p manipulation.**

Data were presented as mean±SD, ***P* < 0.01, Student’s *t* test.

**Supplementary Table S1: Data acquisition:**

| **Data** | **Source** | **Accession number** | **Reference (PMID)** |
| --- | --- | --- | --- |
| HNSCC gene expression array | GEO | GSE41613 | 35464860 |
| HNSCC gene expression array | GEO | GSE42743 | 23319825 |
| HNSCC gene expression array | GEO | GSE65858 | 26095926 |
| HNSCC bulk RNA-seq | GEO | GSE186775 | 37900177 |
| HNSCC scRNA-seq | GEO | GSE234933 | 37535729 |
| Cal27 H3K27ac ChIP-seq | GEO | GSE128275 | 31444232 |
| HN12 H3K27ac ChIP-seq | GEO | GSE128275 | 31444232 |
| HSC3 H3K27ac ChIP-seq | GEO | GSE178613 | 34650128 |
| SAS H3K27ac ChIP-seq | GEO | GSE205455 | 36012427 |
| HIOEC H3K27ac ChIP-seq | GEO | GSE139805 | 32031521 |
| HKC H3K27ac ChIP-seq | GEO | GSE59827 | 26034101 |
| HSC3 BRD4 ChIP-seq | GEO | GSE205455 | 36012427 |
| SAS BRD4 ChIP-seq | GEO | GSE205455 | 36012427 |
| Cal27 ATAC-seq | GEO | GSE128275 | 31444232 |
| SCC25 H3K27ac HiChIP | GEO | GSE185532 | 35927986 |
| HNSCC1 H3K27ac ChIP-seq | GSA for Human | HRA007168 | 39206755  Our previous research |
| HNSCC2 H3K27ac ChIP-seq | GSA for Human | HRA007168 | 39206755  Our previous research |
| NOM1 H3K27ac ChIP-seq | GSA for Human | HRA007168 | 39206755  Our previous research |
| FaDu Hi-C | GEO | GSE211296 | 36650703 |
| OSCC sample Hi-C | GSA for Human | HRA008185 | 39243015 |
| Cal27 si-NC1 rep1 RNA-seq | GSA for Human | HRA011140 | This research |
| Cal27 si-NC1 rep2 RNA-seq | GSA for Human | HRA011140 | This research |
| Cal27 si-Linc00973-1 RNA-seq | GSA for Human | HRA011140 | This research |
| Cal27 si-Linc00973-2 RNA-seq | GSA for Human | HRA011140 | This research |
| Cal27 si-NC2 rep1 RNA-seq | GSA for Human | HRA011140 | This research |
| Cal27 si-NC2 rep2 RNA-seq | GSA for Human | HRA011140 | This research |
| Cal27 si-EN2-1 RNA-seq | GSA for Human | HRA011140 | This research |
| Cal27 si-EN2-2 RNA-seq | GSA for Human | HRA011140 | This research |
| HN6 si-NC2 rep1 RNA-seq | GSA for Human | HRA011140 | This research |
| HN6 si-NC2 rep2 RNA-seq | GSA for Human | HRA011140 | This research |
| HN6 si-EN2-1 RNA-seq | GSA for Human | HRA011140 | This research |
| HN6 si-EN2-2 RNA-seq | GSA for Human | HRA011140 | This research |

**Supplementary Table S2: shRNA and siRNA sequences:**

| **RNAi** | **Target** | **Sequence (5’ to 3’)** |
| --- | --- | --- |
| si-NC/sh-NC | - | GGAUCCTGUCUUGCGUAUCTT |
| si-LINC00973-1  /sh-LINC00973-1 | Human LINC00973 | GUACCAGCUUUCAAAUUUATT |
| si-LINC00973-2  /sh-LINC00973-2 | Human LINC00973 | CACGACUUCUGGUCAUUUATT |
| si-EN2-1 | Human EN2 | GAAAGACCAAUCCAACUUUTT |
| si-EN2-2 | Human EN2 | GCCUCAACGAGUCACAGUUTT |
| si-FOSL1 | Human FOSL1 | GTGGATGGTACAGCCTCATTT |

**Supplementary Table S3. qRT-PCR primer sequences:**

| **Target (human)** | **Forward (5’ to 3’)** | **Reverse (5’ to 3’)** |
| --- | --- | --- |
| LINC00973 | ATGAAGCCACAGAGATTTGCT | AGCCTTCAATTCCAGGGAAAG |
| GAPDH | AGGTGAAGGTCGGAGTCAAC | AGTTGAGGTCAATGAAGGGG |
| U6 | CTCGCTTCGGCAGCACA | AACGCTTCACGAATTTGCGT |
| EN2 | AGGAGCTGAGCCTCAACGAGTC | CTTGGCTGTGGTGGAGTGGTTG |
| FOSL1 | CAGGCGGAGACTGACAAACTG | TCCTTCCGGGATTTTGCAGAT |
| 18S rRNA | ACACGGACAGGATTGACAGA | GGACATCTAAGGGCATCACA |

**Supplementary Table S4. ChIP-qPCR primer sequences:**

| **Target (human)** | **Forward (5’ to 3’)** | **Reverse (5’ to 3’)** |
| --- | --- | --- |
| LINC00973-E1 | ACTTGTCCCAGGGGATCCAT | GCAATGCCCACCATGGAATG |
| LINC00973-E2 | ACACATGTCAGAGTCAGCGG | AACGTGAATTCCCCCATGCT |
| LINC00973-E3 | GGAGGCAGGATGTGGTGTTA | TTGGTGCCAAGTTGAGGCAG |
| LINC00973-E4 | GCTAGGCACGACTTCTGGTC | GCCAGAGATCAGGGTTGACA |
| LINC00973-E-NC | CTCCCTGTTGGTTCTGTCACTT | TTAGGTCCTTGTGTGGTGTCC |

**Supplementary Table S5. sgRNA sequences:**

| **Target (human)** | **Sequence (5’ to 3’)** |
| --- | --- |
| LINC00973-E1 | AGTGGCCGTCAATTCATGCA |
| LINC00973-E2 | TGAATTCCCCCATGCTCAGT |
| LINC00973-E3 | TTAATGACCTAACTAGATCT |
| LINC00973-E4 | GAAAAGACTTGGCTTAAGTT |
