# Supplementary material for: A novel super-enhancer-driven lncRNA LINC00973 governs head and neck squamous cell carcinoma progression through EN2
Source: Cell Death Dis. 2025 Dec 19;17(1):111. doi: 10.1038/s41419-025-08380-8 (PMC12848070; doi:10.1038/s41419-025-08380-8)

Figure 2h

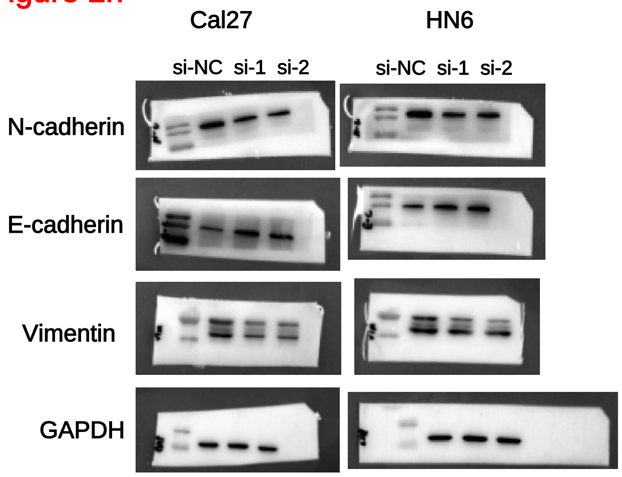

Figure 3d

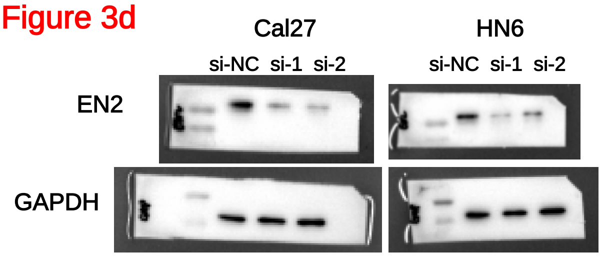

Figure 3h

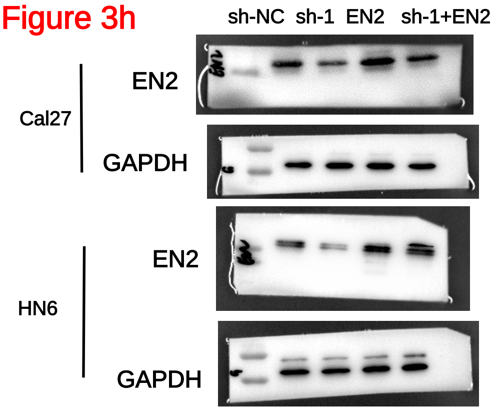

Figure 3i

Cal27 HN6

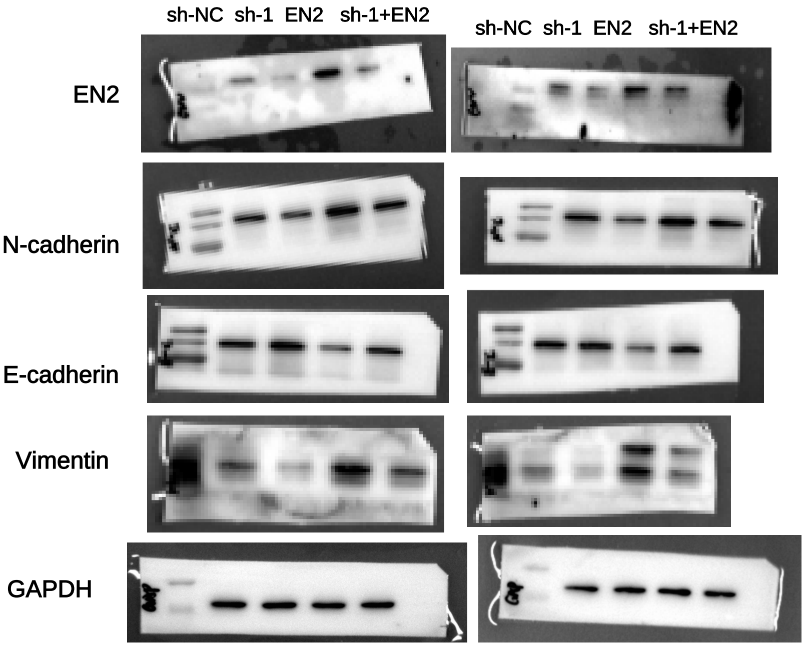

Cal27 HN6

Figure 5i

Input IgG FOSL1 Input IgG FOSL1

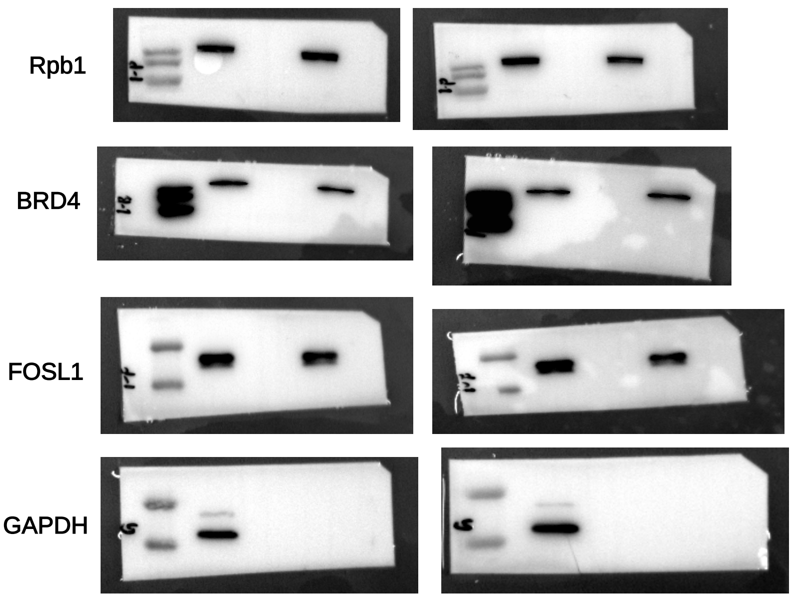

Figure 5j

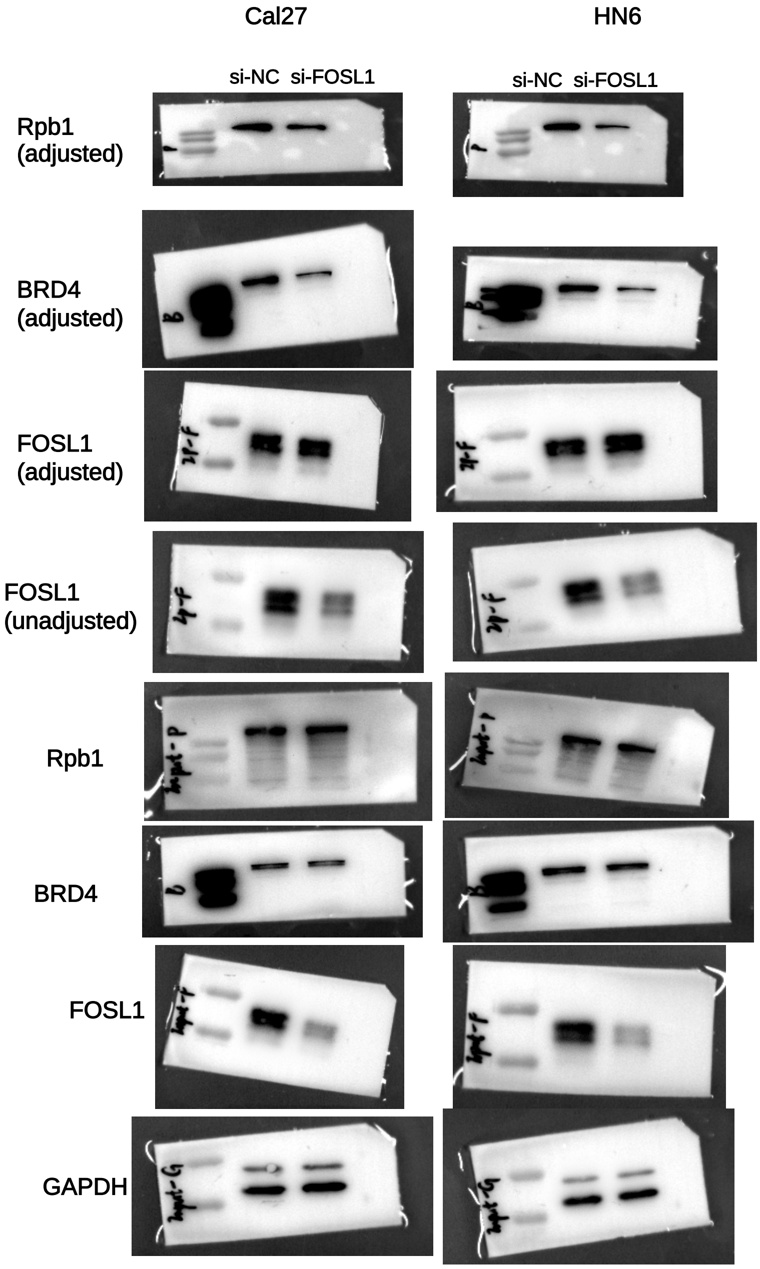

Figure 6e

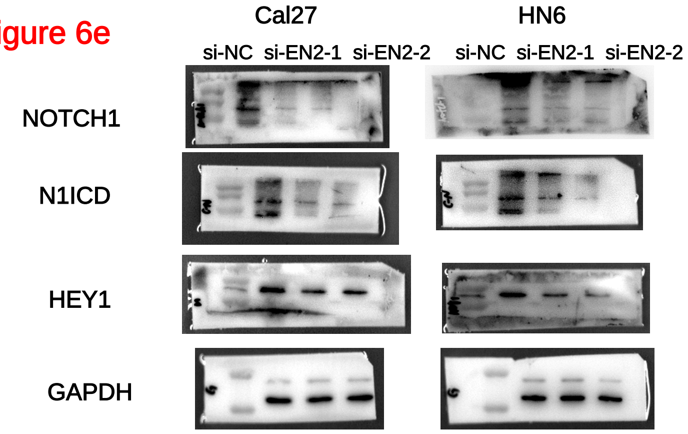

Figure 6f

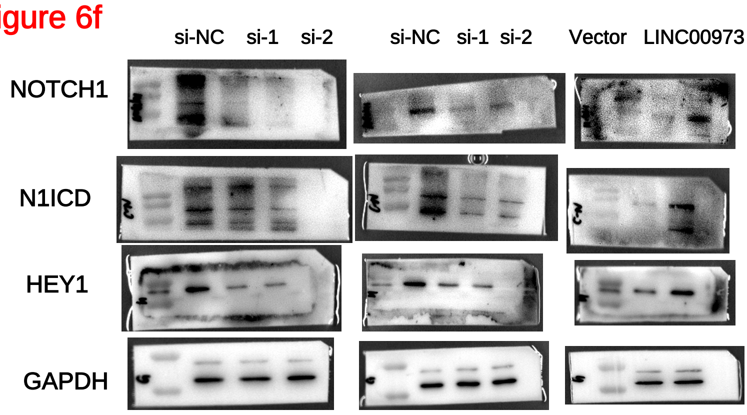

Figure 6g

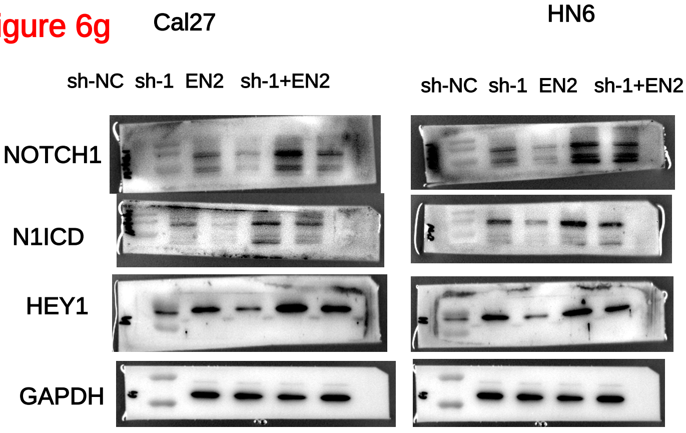

Supplementary_ Figure 3b

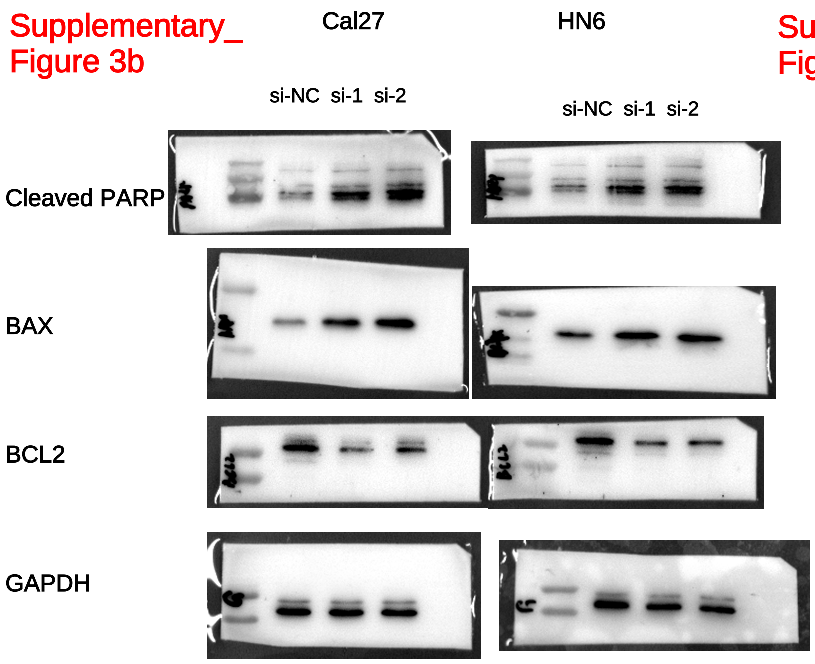

Supplementary_ Figure 11

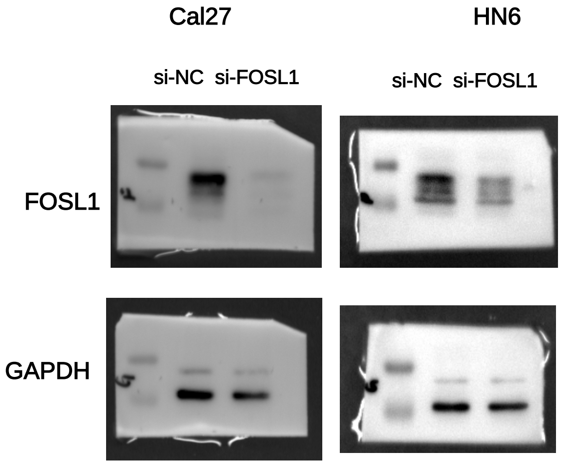

Supplement: Supplementary file 2 — Uncropped Gels and Blots image [file 41419_2025_8380_MOESM2_ESM.pdf]
