# Supplementary figures and images for: A novel super-enhancer-driven lncRNA LINC00973 governs head and neck squamous cell carcinoma progression through EN2
Source: Cell Death Dis. 2025 Dec 19;17(1):111. doi: 10.1038/s41419-025-08380-8 (PMC12848070; doi:10.1038/s41419-025-08380-8)

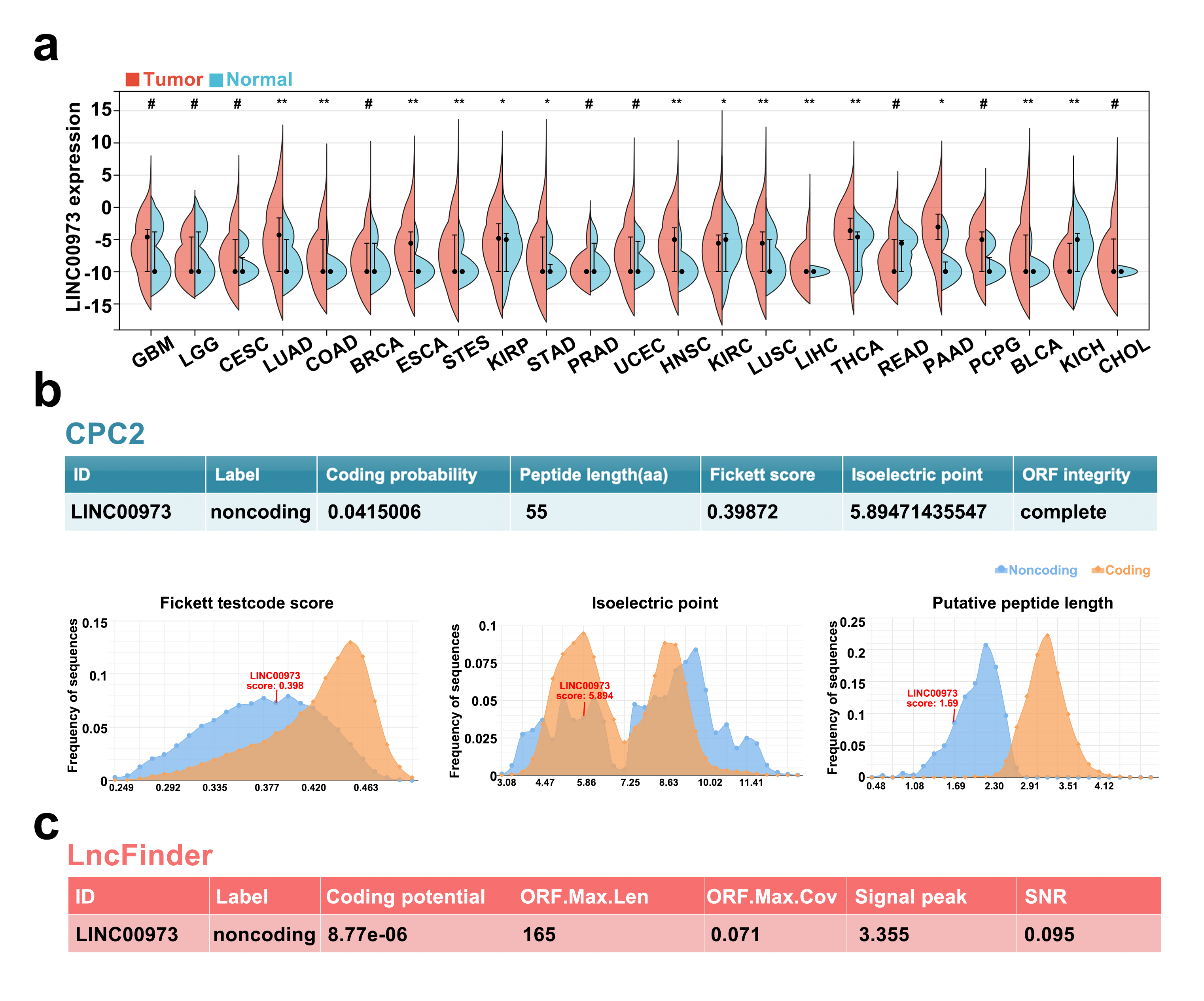

Supplement: Supplementary file 3 — Supplementary_Figure 1 [file 41419_2025_8380_MOESM3_ESM.tif]

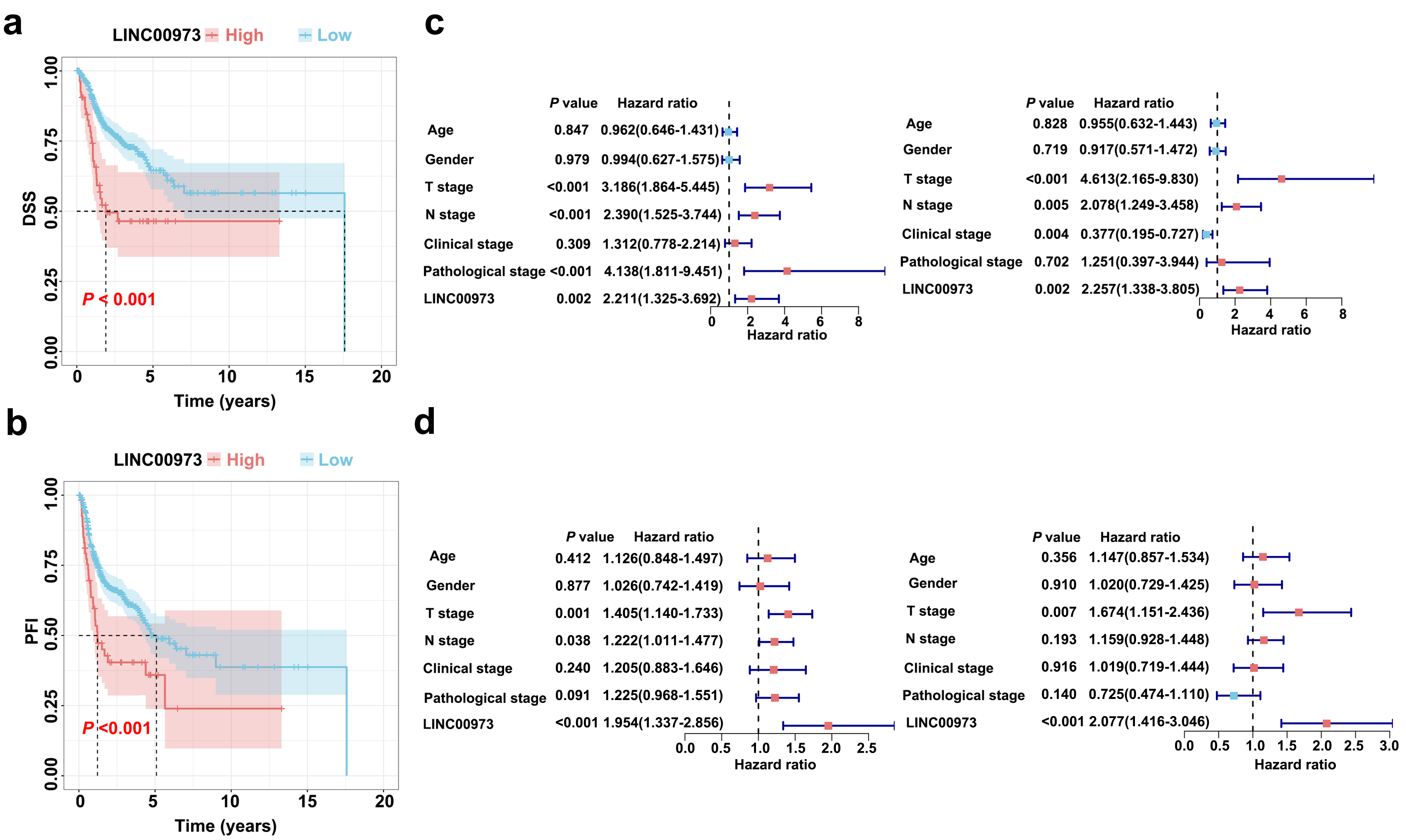

Supplement: Supplementary file 4 — Supplementary_Figure 2 [file 41419_2025_8380_MOESM4_ESM.tif]

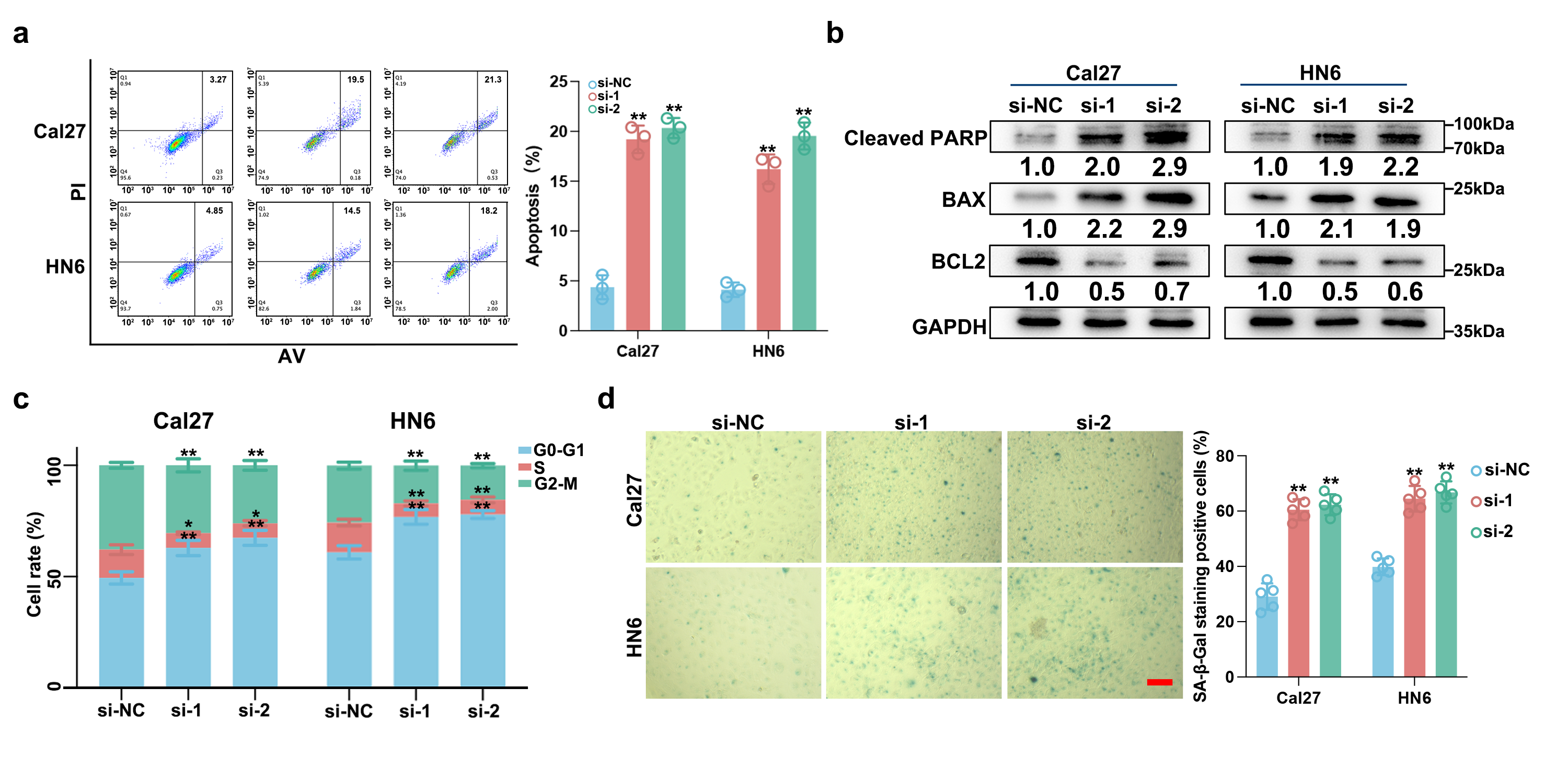

Supplement: Supplementary file 5 — Supplementary_Figure 3 [file 41419_2025_8380_MOESM5_ESM.tif]

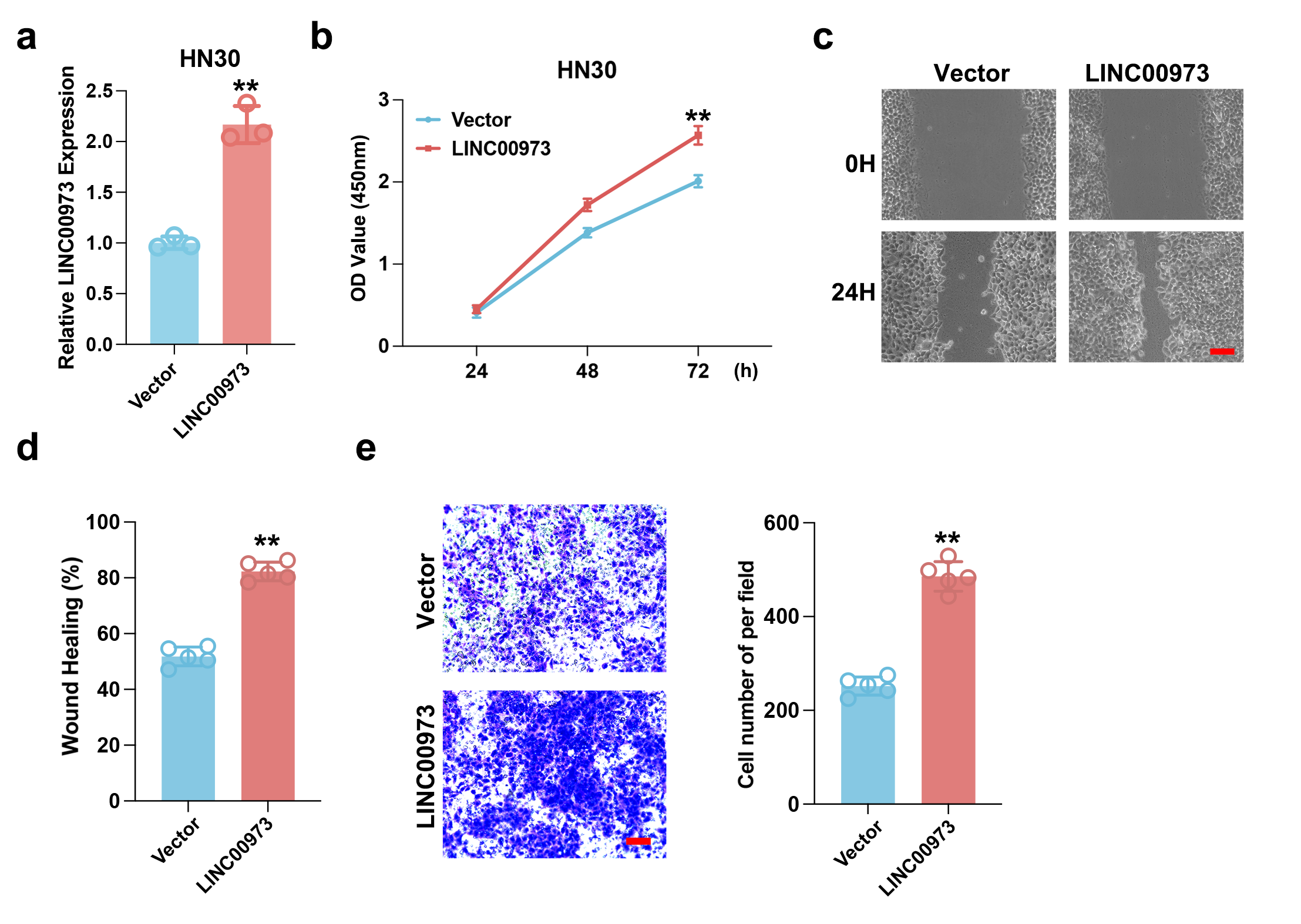

Supplement: Supplementary file 6 — Supplementary_Figure 4 [file 41419_2025_8380_MOESM6_ESM.tif]

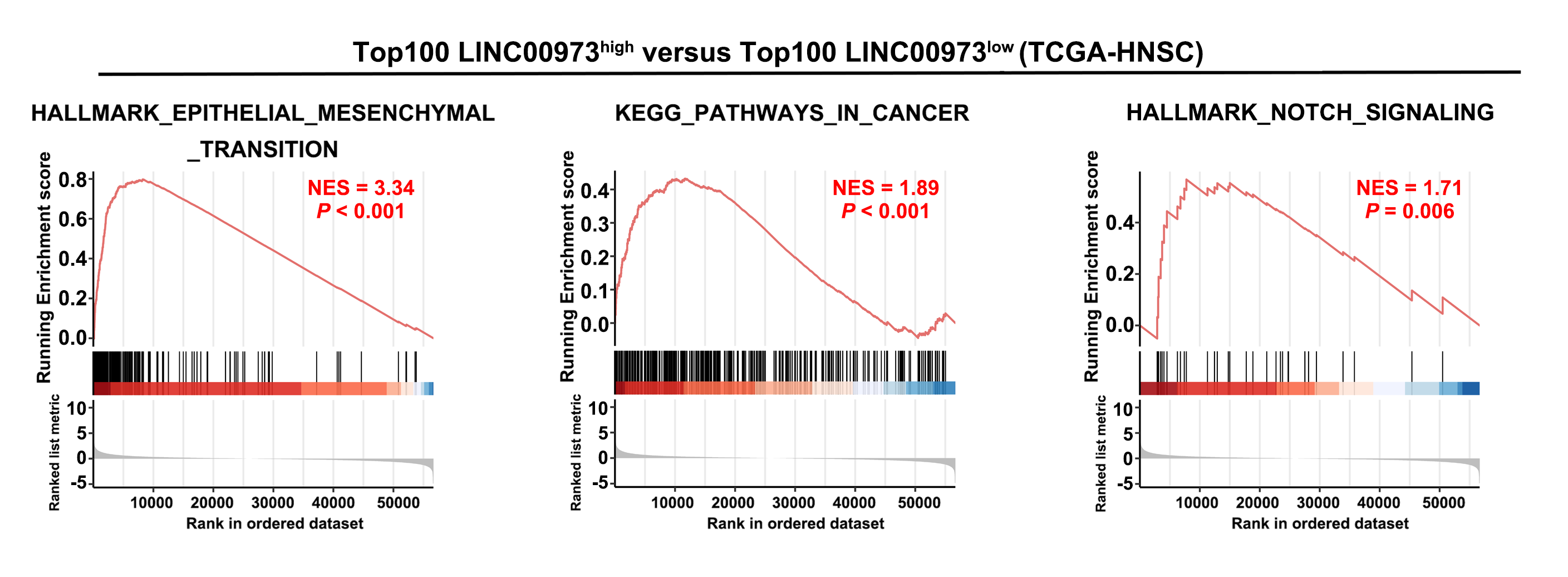

Supplement: Supplementary file 7 — Supplementary_Figure 5 [file 41419_2025_8380_MOESM7_ESM.tif]

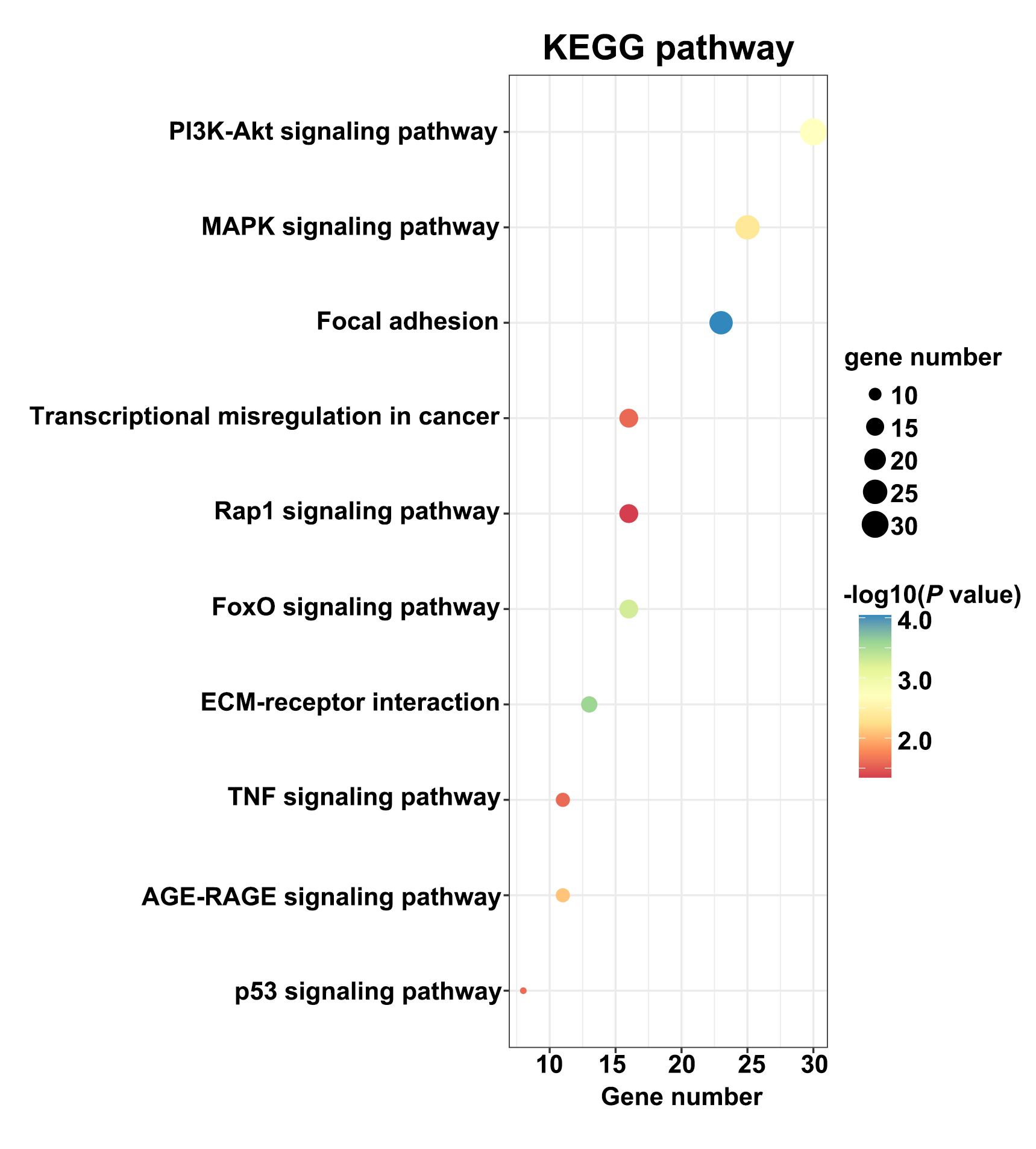

Supplement: Supplementary file 8 — Supplementary_Figure 6 [file 41419_2025_8380_MOESM8_ESM.tif]

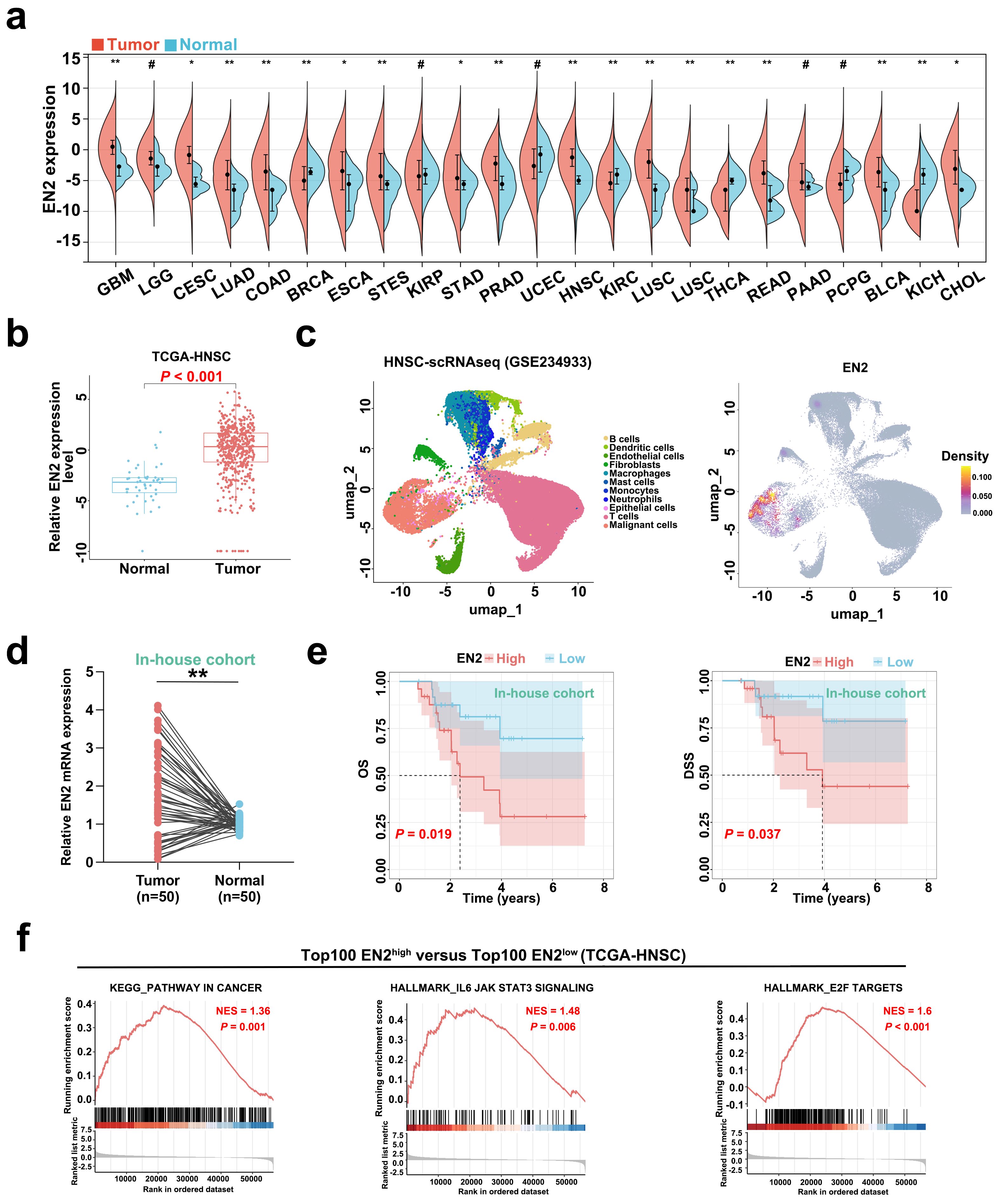

Supplement: Supplementary file 9 — Supplementary_Figure 7 [file 41419_2025_8380_MOESM9_ESM.tif]

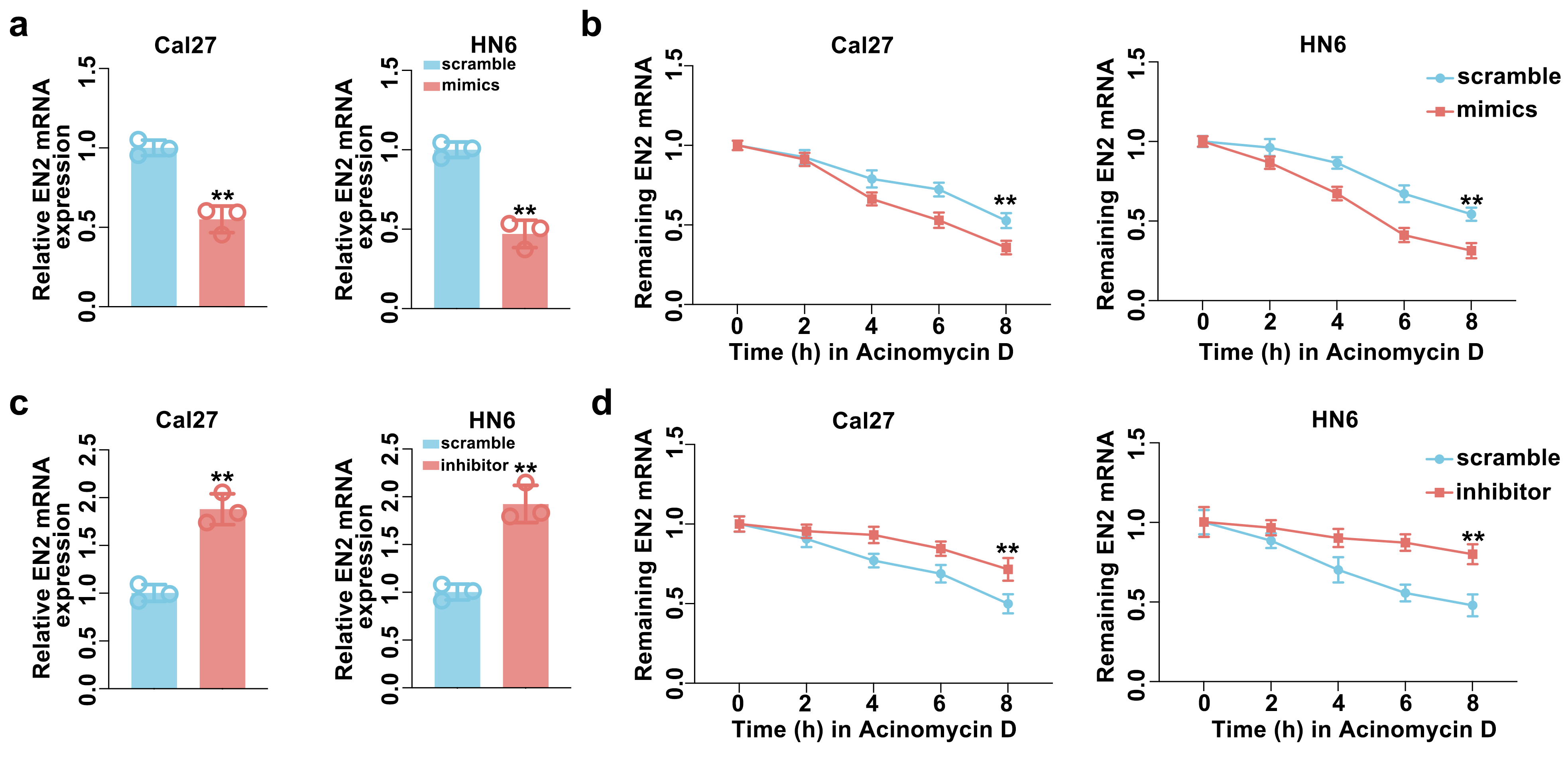

Supplement: Supplementary file 10 — Supplementary_Figure 8 [file 41419_2025_8380_MOESM10_ESM.tif]

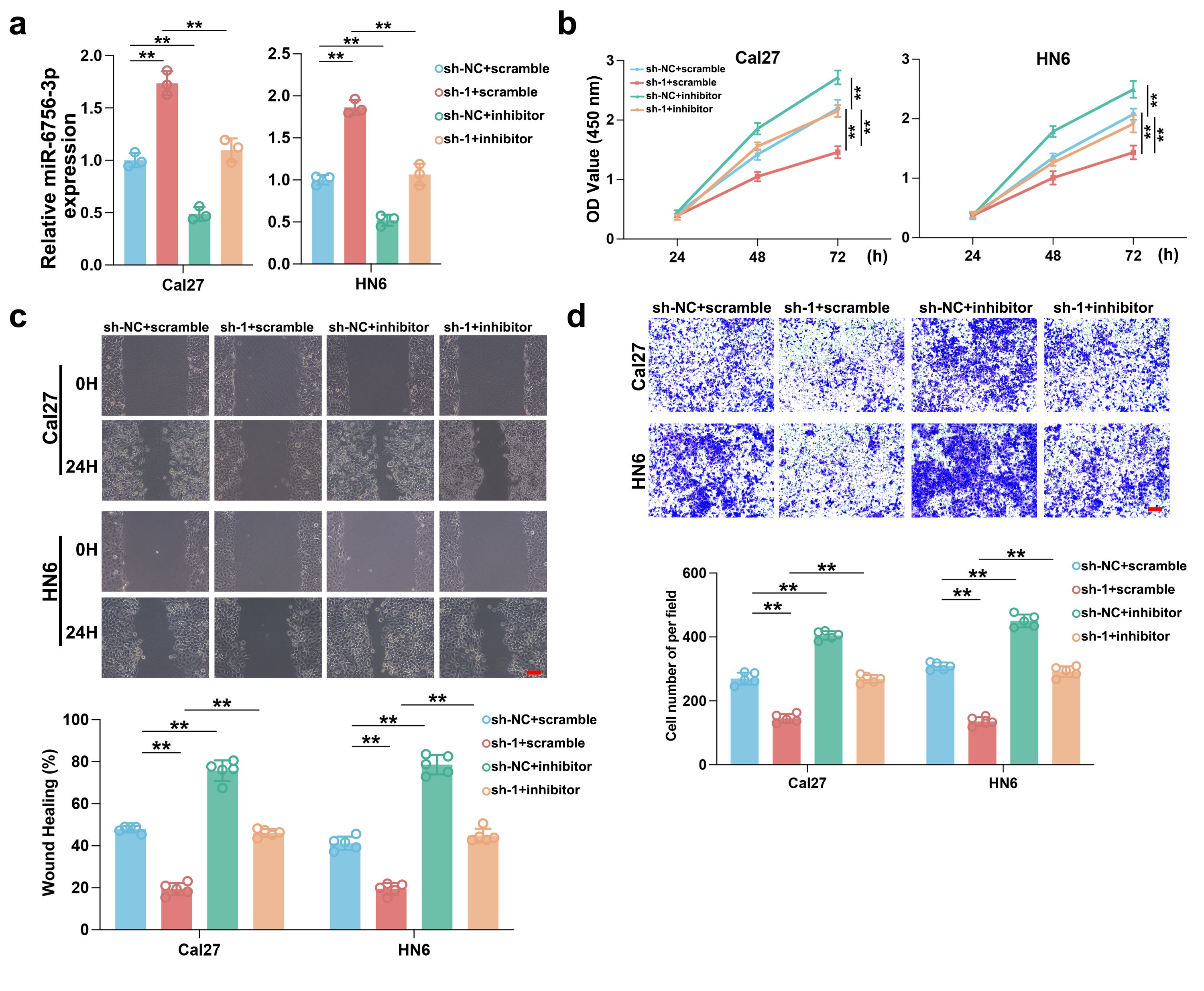

Supplement: Supplementary file 11 — Supplementary_Figure 9 [file 41419_2025_8380_MOESM11_ESM.tif]

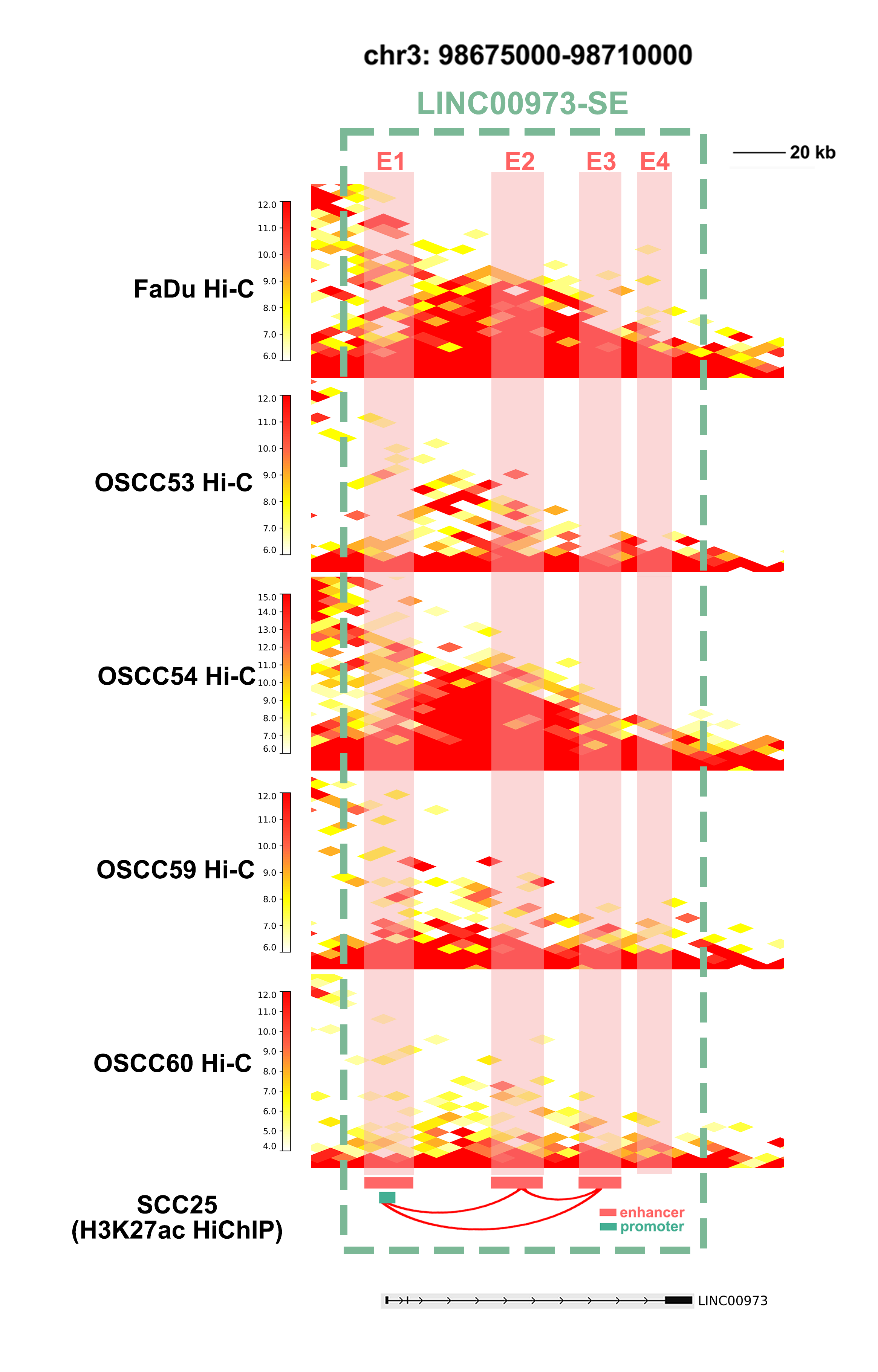

Supplement: Supplementary file 12 — Supplementary_Figure 10 [file 41419_2025_8380_MOESM12_ESM.tif]

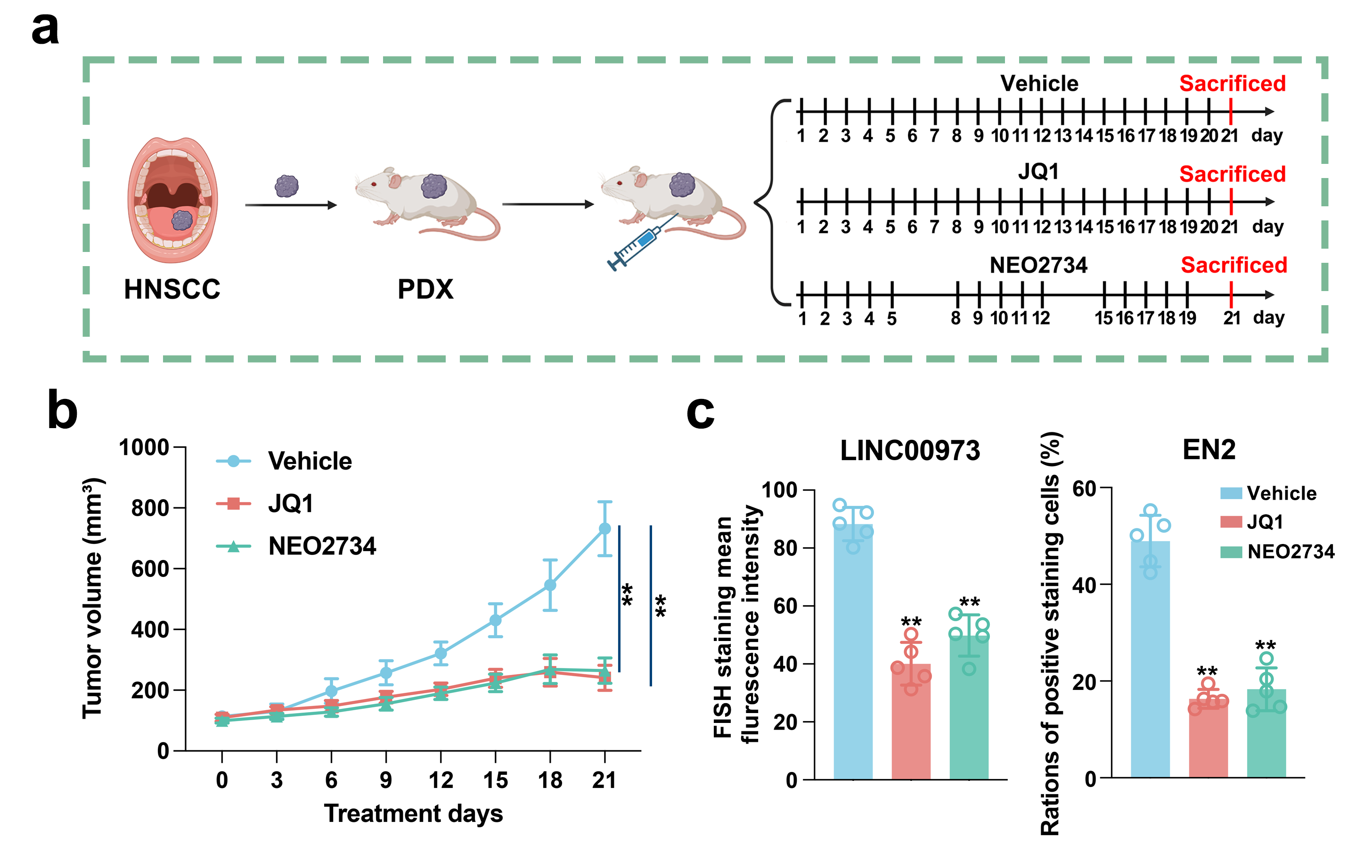

Supplement: Supplementary file 13 — Supplementary_Figure 11 [file 41419_2025_8380_MOESM13_ESM.tif]

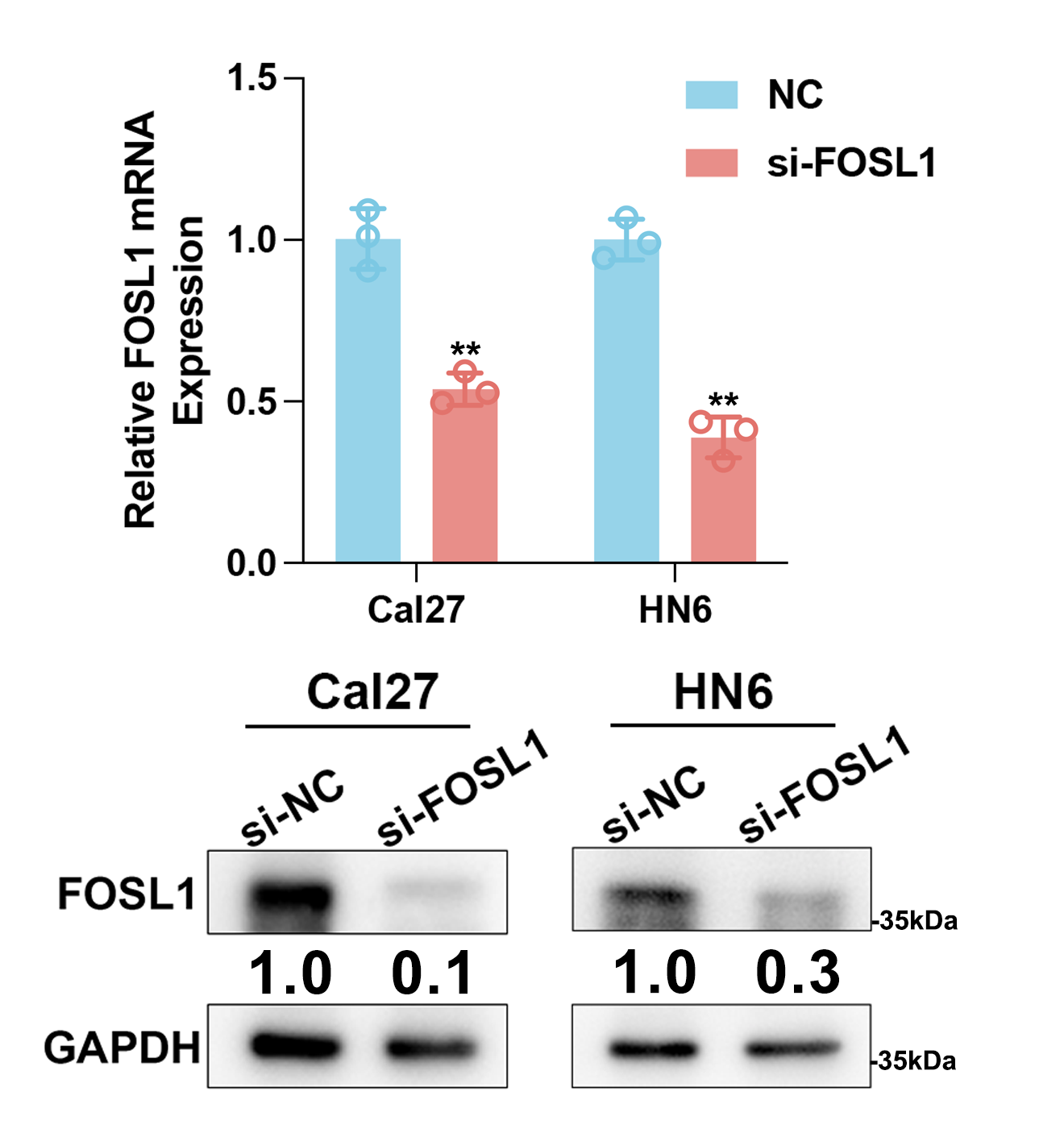

Supplement: Supplementary file 14 — Supplementary_Figure 12 [file 41419_2025_8380_MOESM14_ESM.tif]

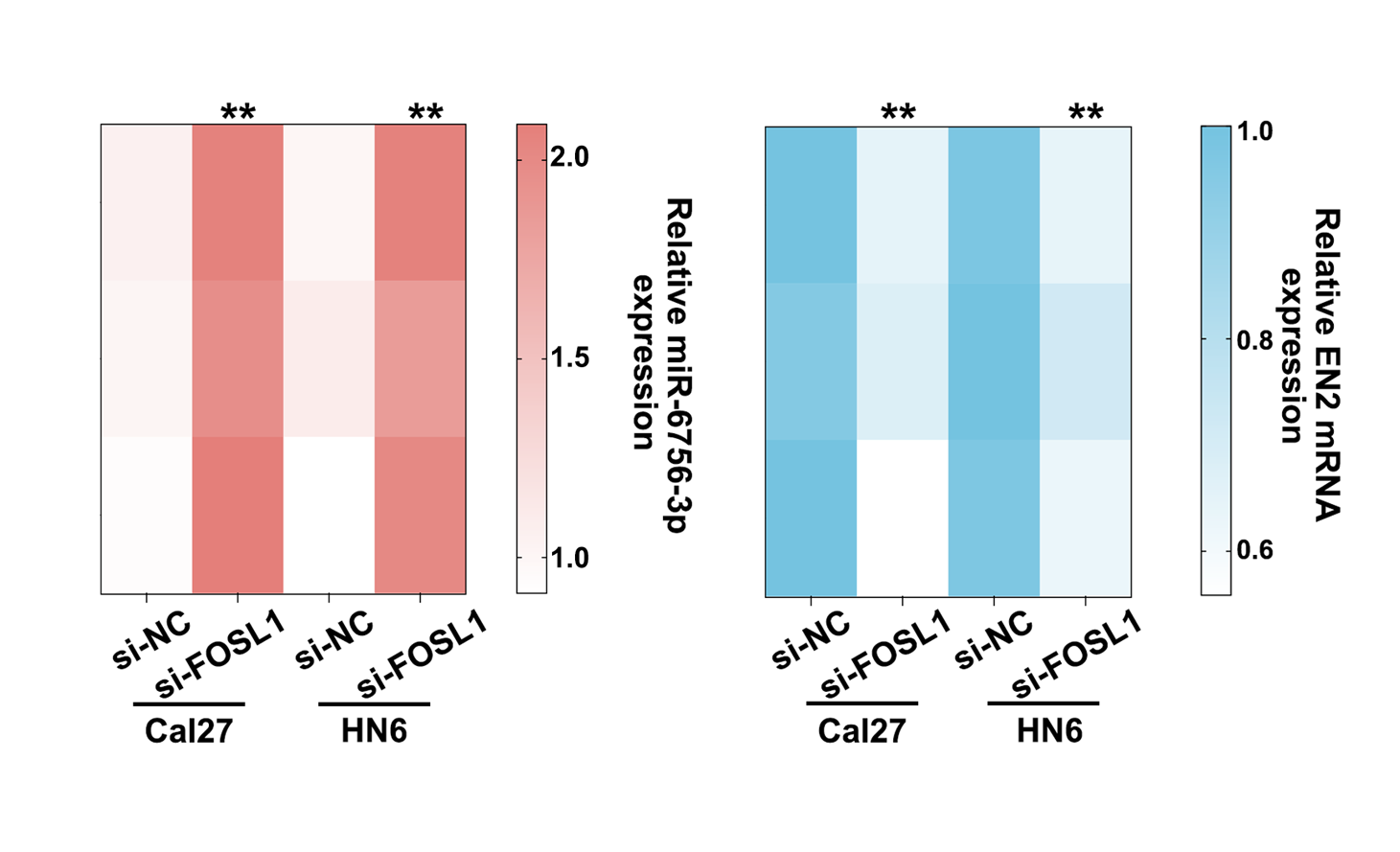

Supplement: Supplementary file 15 — Supplementary_Figure 13 [file 41419_2025_8380_MOESM15_ESM.tif]
